# Urinary Proteomic Biomarkers for Diagnosis and Risk Stratification of Autosomal Dominant Polycystic Kidney Disease: A Multicentric Study

**DOI:** 10.1371/journal.pone.0053016

**Published:** 2013-01-10

**Authors:** Andreas D. Kistler, Andreas L. Serra, Justyna Siwy, Diane Poster, Fabienne Krauer, Vicente E. Torres, Michal Mrug, Jared J. Grantham, Kyongtae T. Bae, James E. Bost, William Mullen, Rudolf P. Wüthrich, Harald Mischak, Arlene B. Chapman

**Affiliations:** 1 Division of Nephrology, University Hospital, Zürich, Switzerland; 2 Mosaiques Diagnostics and Therapeutics AG, Hannover, Germany; 3 Division of Nephrology and Hypertension, Department of Medicine, Mayo Clinic, Rochester, Minnesota, United States of America; 4 Division of Nephrology, University of Alabama at Birmingham, Birmingham, Alabama, United States of America; 5 Department of Veterans Affairs Medical Center, Birmingham, Alabama, United States of America; 6 Kidney Institute and Department of Internal Medicine, Kansas University Medical Center, Kansas City, Missouri, United States of America; 7 Department of Radiology, University of Pittsburgh, Pittsburgh, Pennsylvania, United States of America; 8 Department of Medicine, University of Pittsburgh, Pittsburgh, Pennsylvania, United States of America; 9 Booz Allen Hamilton, Rockville, Maryland, United States of America; 10 BHF Glasgow Cardiovascular Research Centre, University of Glasgow, Glasgow, United Kingdom; 11 Division of Nephrology, Department of Medicine, Emory University School of Medicine, Atlanta, Georgia, United States of America; Moffitt Cancer Center, United States of America

## Abstract

Treatment options for autosomal dominant polycystic kidney disease (ADPKD) will likely become available in the near future, hence reliable diagnostic and prognostic biomarkers for the disease are strongly needed. Here, we aimed to define urinary proteomic patterns in ADPKD patients, which aid diagnosis and risk stratification. By capillary electrophoresis online coupled to mass spectrometry (CE-MS), we compared the urinary peptidome of 41 ADPKD patients to 189 healthy controls and identified 657 peptides with significantly altered excretion, of which 209 could be sequenced using tandem mass spectrometry. A support-vector-machine based diagnostic biomarker model based on the 142 most consistent peptide markers achieved a diagnostic sensitivity of 84.5% and specificity of 94.2% in an independent validation cohort, consisting of 251 ADPKD patients from five different centers and 86 healthy controls. The proteomic alterations in ADPKD included, but were not limited to markers previously associated with acute kidney injury (AKI). The diagnostic biomarker model was highly specific for ADPKD when tested in a cohort consisting of 481 patients with a variety of renal and extrarenal diseases, including AKI. Similar to ultrasound, sensitivity and specificity of the diagnostic score depended on patient age and genotype. We were furthermore able to identify biomarkers for disease severity and progression. A proteomic severity score was developed to predict height adjusted total kidney volume (htTKV) based on proteomic analysis of 134 ADPKD patients and showed a correlation of r = 0.415 (p<0.0001) with htTKV in an independent validation cohort consisting of 158 ADPKD patients. In conclusion, the performance of peptidomic biomarker scores is superior to any other biochemical markers of ADPKD and the proteomic biomarker patterns are a promising tool for prognostic evaluation of ADPKD.

## Introduction

Autosomal dominant polycystic kidney disease (ADPKD) is the most frequent hereditary kidney disease, affecting between 1 in 400 and 1 in 1000 individuals of the general population [1], [2]. The growth of innumerable cysts in both kidneys causes progressive kidney dysfunction leading to end stage renal disease (ESRD) by the sixth decade in 50% of affected patients [3]. The disease is caused by mutations in the PKD1 (85% of cases) or the PKD2 gene (15% of cases).

The disease course of ADPKD is characterized by high inter- and intra-familial variability that hampers the prediction of disease progression [4]. Affected individuals may retain adequate renal function until their 9th decade, whereas others progress to ESRD by their 3rd decade. Genetic modifiers as well as environmental factors are likely to influence the disease course, although information on these factors is sparse and the currently known factors only account for a small proportion of the predictive power for prognosis [5], [6], [7]. In particular, glomerular filtration rate (GFR) remains stable for many decades in the early disease stages, when predicting disease progression would be most valuable for counseling ADPKD patients [8]. During the last decade, several pathways involved in the generation and growth of cysts in ADPKD have been unraveled and several of these pathways have led to the development of targeted medical therapies [9]. Specific treatment options, such as the vasopressin antagonist tolvaptan, somatostatin analogues, and angiotensin converting enzyme inhibitors or angiotensin receptor blockers are currently being evaluated in large clinical trials that await completion or publication and may become available in the near future, whereas other therapeutic options, such as the cyclin dependent kinase inhibitor roscovitine, are in preclinical development. Since these treatments will most likely need to be given over long periods of time, prognostic evaluation of patients will gain further importance, particularly since the potential therapeutic benefits need to be balanced against side effects and costs.

The diagnosis of ADPKD is usually based on the observation of kidney cysts by ultrasound in patients with positive family history for ADPKD [10]. However, ultrasound imaging has limited sensitivity in children and young adults, particularly those with PKD2 mutations, and thus ADPKD cannot be reliably excluded by ultrasound before the age of 30 years [10]. Furthermore molecular diagnosis by genetic testing has been hampered by the genetic complexity of ADPKD, and only 65% of ADPKD patients exhibit definitive pathogenic (i.e. truncating) mutations [11].

Proteomic analysis of urine offers a noninvasive means to simultaneously detect changes in the expression and processing of multiple proteins [12]. In contrast to other body fluids, such as serum or plasma, the urinary proteome does not undergo detectable degradation by endogenous proteases after voiding, thus minimizing the bias introduced by preanalytical sample handling [13]. CE-MS analysis of over 10,000 individual urine samples demonstrated high stability and consistency of the urinary low molecular weight proteome [14]. Through the simultaneous measurement of hundreds of polypeptides followed by appropriate statistical analysis, a combination of distinct biomarkers in a classifier, rather than single biomarkers, can be developed, which largely increases sensitivity and specificity in comparison to the singla markers. Urinary biomarkers and biomarker-based classifiers could be validated in several independent studies [15], [16], [17], further supporting the validity of the approach and demonstrating the stability of the human urinary proteome/peptidome.

We have previously identified a urinary polypeptide pattern characteristic of ADPKD using capillary electrophoresis coupled online to mass spectrometry (CE-MS) [18]. Here, we sought to validate these findings in the large prospective ADPKD cohort of the Consortium for Radiologic Imaging Studies in Polycystic Kidney Disease (CRISP) and to develop a biomarker model for disease severity that may aid prognostic evaluation.

## Results

**Figure 1:**
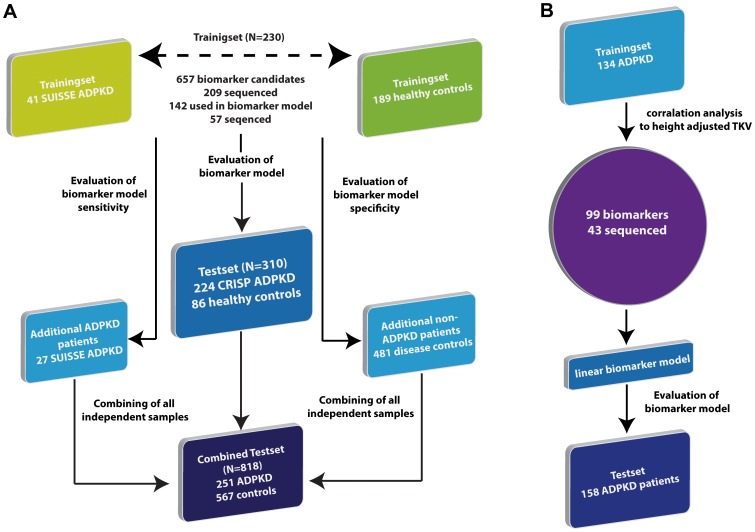
Usage of samples and flow of information. A, Identification and validation of diagnostic biomarkers and biomarker models. 41 cases of ADPKD were compared to 189 healthy controls, which resulted in the definition of 657 potential biomarkers. Of these, 142 were employed in an SVM-driven biomarker model, ADPKD_142. All potential biomarkers and the biomarker model were evaluated in a test set of 310 blinded samples that consisted of 224 samples from patients with ADPKD and 86 healthy controls. The ADPKD_142 model was further validated using additional ADPKD samples from the SUISSE ADPKD study (n = 27) and using controls samples of patients with a variety of different renal and systemic diseases. B, Identification and validation of biomarkers and biomarker model for disease severity. CE-MS data from 135 urine samples from patients with ADPKD were correlated with height adjusted TKV (htTKV), resulting in the identification of 99 potential biomarkers associated with htTKV. Employing linear combination, a biomarker models indicative of disease severity was established. This biomarker model was subsequently tested in a validation set consisting of 153 ADPKD samples.

The design of the study, samples used and the flow of the data are graphically depicted in **Figure 1**. In total, spot urine samples from 224 CRISP patients [19], 68 patients of the SUISSE ADPKD study [20],275 healthy controls (mean age 37±15 years, 49% females, all caucasians) and from 481 patients suffering from a variety of non-cystic renal and systemic diseases were analyzed. The demographic data, kidney volume, GFR and clinical characteristics were similar among patients of the CRISP and SUISSE ADPKD cohorts (**Table 1**). The mean available follow-up time after collection of urine for proteomic analysis was 2.99±0.46 (range: 0.98–4.23) years in the CRISP cohort and 2.18±0.49 (range: 1.46–3.37) years in the SUISSE ADPKD cohort.

**Table 1:** Clinical characteristics of all CRISP and SUISSE ADPKD study patients included in the proteomic analysis.

Cohort	SUISSE ADPKD	CRISP
N	68	224
Age	31.4±6.3	32.4±8.7
Sex (% female)	35.8	59.4
Hypertension (%)	70.8	61.6
eGFR	86.4±15.5	89.1±27.8
TKV	1023±592	1078±647
GenotypePKD1PKD2no detectable mutation	not available	78.1%13.8%7.1%

eGFR, estimated glomerular filtration rate according to the MDRD study formula; TKV, total kidney volume. Values are mean ± SD unless otherwise specified.

**Figure 2:**
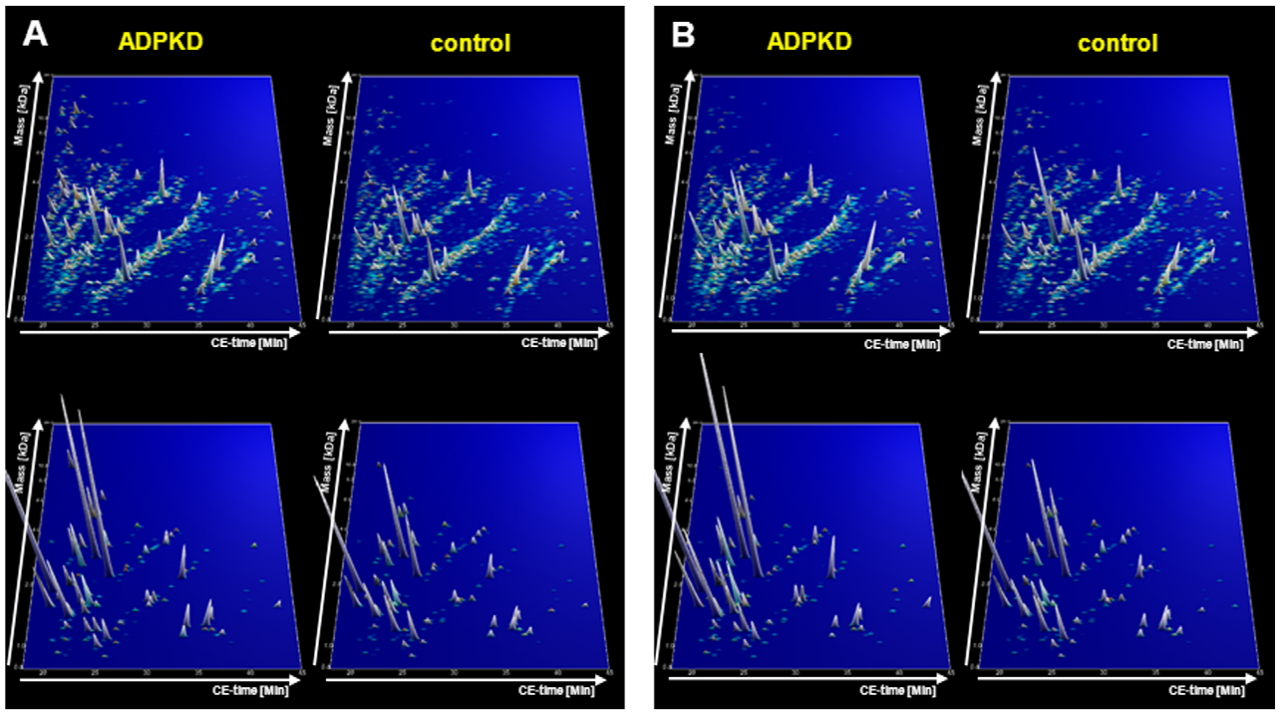
Compiled urinary protein profiles of ADPKD patients and healthy controls. Proteomic profiles for the training cohort (41 patients of the SUISSE ADPKD study vs. 189 controls, panel A) and the validation cohort (224 CRISP study samples vs. 86 controls, panel B) are depicted separately. Normalized MS molecular weight (800–20,000 Da) in logarithmic scale is plotted against normalized CE migration time (18–45 min). The mean signal intensity of polypeptides is given as peak height. In the lower panels, only the 142 biomarkers that were included in the diagnostic biomarker model are depicted, and their amplitude is shown with 5× zoom compared to the upper panels.

Since the previously published biomarker model for ADPKD [18] was based on a relatively small number of patients (n = 17), we now based our analysis on a larger number of urine samples, aiming to identify additional urinary peptides that are altered in ADPKD and to assure an adequate number of individuals to develop a robust biomarker score. We compared peptidome data of 41 SUISSE ADPKD patients to 189 healthy controls (mean age 37±15 years, 49% females). Compiled urinary proteomic patterns of ADPKD and control patients are given in **Figure 2**. Statistical comparison of cases and controls resulted in the identification of 657 peptides that were significantly different between the two groups after adjustment for multiple testing. Of these, 209 could be sequenced using high-resolution tandem mass spectrometry. Most biomarker candidates were collagen fragments, possibly reflecting substantial alteration in extracellular matrix (ECM) turnover. The CE-MS characteristics of all differentially excreted peptides, their regulation in ADPKD, and where applicable their sequence are given in **Table S1**.

**Figure 3:**
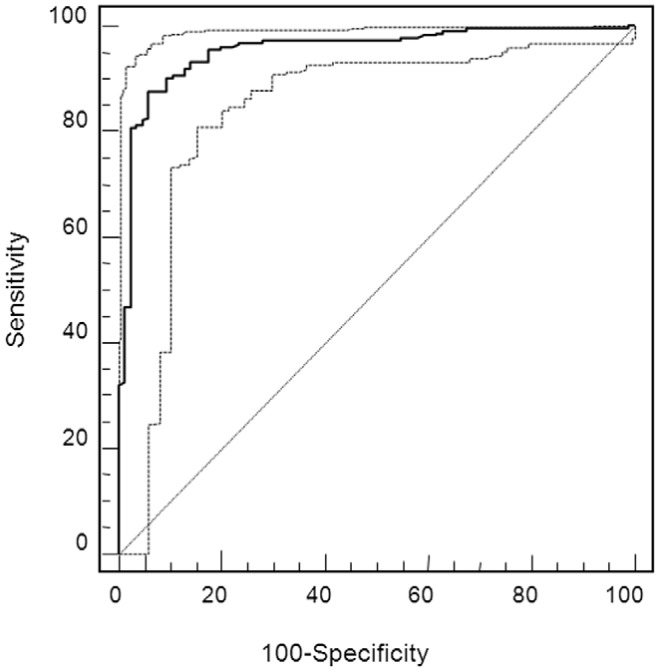
ROC curve with 95% CI for the differentiation of ADPKD patients from healthy controls by the biomarker model ADPKD_142 applied to the CRISP validation cohort and 86 healthy individuals.

Based on these peptides we next established a support-vector-machine (SVM)-based diagnostic score. Because the number of potential biomarkers substantially exceeded the number of samples in the study, we reduced the number of variables for the biomarker model to the most consistently altered 142 peptides using a “take-one-out” procedure in the total cross-validation of the training data. Of these 142 peptides, 57 could be identified by means of their peptide sequence (**Table 2**). The SVM-based model combines the amplitude of all 142 markers for a given urine sample into a score, which denotes the distance of that sample in a 142-dimensional space (every dimension representing the abundance of one peptide) from a hyperplane that is designed to separate the cases from controls. The parameters of the kernel function for the 141-dimensional hyperplane were: cost (C) of 640 and kernel width (γ) of 0.000003. Of these 142 markers, 23 had been among the markers used in the previously published ADPKD_38 model [18]. The SVM-based diagnostic model, ADPKD_142, yielded an area under the receiver operator characteristics curve (AUC) of 0.98 in the training cohort using total take-one-out cross validation. Upon validation in the independent CRISP cohort and 86 healthy controls, the model achieved an AUC of 0.95 (95% confidence interval [CI] 0.92–0.98), corresponding to a sensitivity of 84.4% and a specificity of 94.2% when using a predefined cutoff value that yielded optimal sensitivity and specificity in the cross-validated training data (**Figure 3**). A sensitivity analysis for potential center bias was performed by applying the biomarker model to 27 SUISSE ADPKD patients that were not used to generate the model, yielding similar sensitivity (85.2%) as for the CRISP cohort. Combination of CRISP patients and these 27 SUISSE ADPKD patients to validate the model resulted in an overall sensitivity of 84.5%.

**Table 2:** Sequenced biomarkers used in the SVM-based model.

		Training set		Test set			
Mass (Da)	CE-Time (Min)	p-value (BH)	Fold change	p-value (BH)	Fold change	Sequence	Protein name
1613.82	23.99	1.13E-02	0.62963	4.57E-01	0.926	VGGGEQPPPAPAPRRE	Xylosyltransferase 1
1580.88	23.87	1.18E-03	0.36086	2.45E-08	0.5575	IDQSRVLNLGPITR	Uromodulin
1588.71	30.15	3.75E-07	7.16036	1.18E-07	1.5048	TGLSMDGGGSPKGDVDP	Sodium/potassium-transporting ATPase subunit gamma
1715.98	20.93	1.58E-02	1.67142	5.18E-01	0.8368	VRYTKKVPQVSTPTL	Serum albumin
3202.43	30.6	4.39E-05	12.30155	4.78E-03	1.3896	SSQGGSLPSEEKGHPQEESEESNVSMASLGE	Secretogranin-1
1140.52	25.39	6.78E-06	0.05484	2.60E-02	0.6513	YNKYPDAVAT	Osteopontin
3318.55	30.99	2.27E-03	0.55513	3.16E-02	0.6907	GTSLSPPPESSGSPQQPGLSAPHSRQIPAPQGAV	Metastasis-suppressor KiSS-1
2445.1	28.24	2.67E-03	5.02023	1.99E-01	2.4947	mASDASHALEAALEQMDGIIAGTK	Liprin-beta-2
1580.89	24.85	6.58E-03	3.37586	3.80E-10	3.101	LEIELQSLLATKHS	Keratin, type I cytoskeletal 25
1635.76	30.34	7.97E-05	3.39305	3.90E-01	0.9845	FIFPPSDEQLKSGTA	Ig kappa chain C region
1142.56	21.89	2.78E-02	0.28994	3.93E-19	6.056	VSVNERVMPI	Haptoglobin
1882.8	20.24	2.22E-03	2.05957	1.64E-04	1.3966	DEAGSEADHEGTHSTKRG	Fibrinogen alpha chain
984.46	24.92	6.56E-05	6.15717	1.03E-12	2.2448	LAADDPEVR	Ephrin-A1
2889.35	24.08	3.04E-02	3.77113	4.68E-02	1.2247	NGEAGSAGPpGppGLRGSpGSRGLPGADGRAG	Collagen alpha-2(I) chain
3092.44	36.3	1.01E-02	0.44086	1.13E-03	0.6362	TGEVGAVGPpGFAGEKGPSGEAGTAGPpGTpGPQG	Collagen alpha-2(I) chain
1173.53	37.49	3.30E-02	0.54615	8.89E-14	0.096	GPpGPpGPpGPVT	Collagen alpha-1(XVII) chain
1339.6	27.49	1.17E-02	0.67239	6.10E-11	0.2467	SpGERGETGPpGPA	Collagen alpha-1(III) chain
1324.59	28.7	6.56E-05	0.17368	1.38E-01	0.7682	TGPGGDKGDTGPpGP	Collagen alpha-1(III) chain
1623.73	24.12	5.64E-03	1.34979	2.30E-11	1.6404	DGApGKNGERGGpGGpGP	Collagen alpha-1(III) chain
1794.8	23.92	1.11E-03	1.60456	3.71E-17	2.0447	GNDGApGKNGERGGpGGpGP	Collagen alpha-1(III) chain
1989.88	32.44	1.81E-02	0.59974	1.12E-03	0.7419	SNGNpGPpGPSGSpGKDGPpGP	Collagen alpha-1(III) chain
2137.94	21.79	4.48E-03	1.69213	9.63E-09	1.7463	NGEpGGKGERGApGEKGEGGpPG	Collagen alpha-1(III) chain
2264.03	22.67	1.62E-02	2.63884	3.24E-02	1.436	KGDAGApGApGGKGDAGApGERGPpG	Collagen alpha-1(III) chain
2525.2	27.74	2.14E-02	0.45892	9.41E-06	0.5216	LRGGAGPpGPEGGKGAAGPpGPpGAAGTpG	Collagen alpha-1(III) chain
2564.15	22.98	1.11E-03	2.13239	6.52E-08	1.8085	GApGQNGEpGGKGERGApGEKGEGGPpG	Collagen alpha-1(III) chain
2580.14	22.98	8.95E-03	1.83953	3.43E-12	2.0512	GApGQNGEpGGKGERGApGEkGEGGPpG	Collagen alpha-1(III) chain
2823.33	29.12	3.19E-02	0.48201	1.08E-03	0.6638	LRGGAGpPGPEGGKGAAGpPGppGAAGTPGLQG	Collagen alpha-1(III) chain
2825.27	24.49	3.60E-04	1.44938	1.53E-12	1.7677	ERGEAGIpGVpGAKGEDGKDGSpGEpGANG	Collagen alpha-1(III) chain
3255.49	30.78	1.71E-03	3.79654	2.60E-02	1.7139	NTGApGSpGVSGPKGDAGQpGEKGSpGAQGPPGAPGP	Collagen alpha-1(III) chain
3258.46	22.92	6.22E-03	1.88038	1.05E-01	1.5147	ENGKPGEpGpKGDAGApGApGGKGDAGApGERGpPG	Collagen alpha-1(III) chain
911.43	25.88	1.42E-04	0.38645	1.33E-09	0.3484	DGKTGPpGPA	Collagen alpha-1(I) chain
1050.48	26.92	5.68E-03	0.54258	1.73E-11	0.3488	MGPRGPpGPpG	Collagen alpha-1(I) chain
1080.5	25.69	4.18E-03	0.35998	7.44E-02	0.4841	ApGDRGEpGPP	Collagen alpha-1(I) chain
1096.48	26.08	3.79E-04	0.54362	4.00E-10	0.6097	ApGDRGEpGpP	Collagen alpha-1(I) chain
1143.52	36.97	6.47E-04	0.40166	0.00E+00	0.1049	GLPGPpGPpGPpG	Collagen alpha-1(I) chain
1157.54	37.44	2.34E-02	0.67572	0.00E+00	0.1177	GPPGPpGppGPPS	Collagen alpha-1(I) chain
1247.52	22	5.32E-04	1.99799	3.77E-12	2.3055	DKGETGEQGDRG	Collagen alpha-1(I) chain
1297.58	27.36	4.84E-03	0.51815	6.18E-03	0.6989	SpGSpGPDGKTGPp	Collagen alpha-1(I) chain
1458.63	27.94	2.96E-04	0.31301	2.66E-04	0.3525	SpGENGApGQmGPRG	Collagen alpha-1(I) chain
1469.67	23.69	3.04E-02	1.35379	1.50E-12	1.9438	DGQPGAKGEpGDAGAK	Collagen alpha-1(I) chain
1491.74	39.83	1.45E-05	0.31846	2.70E-10	0.3511	VGPpGpPGPPGPPGPPS	Collagen alpha-1(I) chain
1680.75	30.03	3.60E-04	1.67867	1.02E-04	1.3047	TGSpGSpGPDGKTGPpGPA	Collagen alpha-1(I) chain
1684.67	31.75	8.38E-04	0.69637	0.00E+00	0.2026	EpGSpGENGApGQMGPR	Collagen alpha-1(I) chain
2014.9	21.91	1.42E-04	2.00095	5.82E-23	2.384	EGSpGRDGSpGAKGDRGETGP	Collagen alpha-1(I) chain
2128.98	26.97	8.32E-05	0.28973	2.51E-02	0.5359	DGKTGpPGPAGQDGRPGPpGppG	Collagen alpha-1(I) chain
2210.95	33.61	3.36E-02	1.52269	2.69E-09	1.4605	NGApGNDGAKGDAGApGApGSQGApG	Collagen alpha-1(I) chain
2407.09	27.67	1.44E-03	0.63697	5.06E-02	0.9479	LDGAKGDAGPAGPKGEpGSpGENGApG	Collagen alpha-1(I) chain
2471.16	34.77	1.18E-02	0.50314	1.01E-09	0.5201	TGPIGPpGPAGApGDKGESGPSGPAGPTG	Collagen alpha-1(I) chain
2639.29	21.42	5.69E-03	0.41876	4.26E-01	0.8895	KEGGKGPRGETGPAGRpGEVGpPGPpGP	Collagen alpha-1(I) chain
2713.23	29.22	1.37E-02	0.42363	7.24E-01	0.6891	PpGADGQpGAKGEpGDAGAKGDAGPpGPAGP	Collagen alpha-1(I) chain
2767.32	21.67	2.51E-02	0.51467	9.78E-02	0.7323	KEGGKGPRGETGPAGRpGEVGpPGPpGPAG	Collagen alpha-1(I) chain
2942.3	22.23	3.52E-03	1.46282	2.46E-04	1.3039	ESGREGApGAEGSpGRDGSpGAKGDRGETGP	Collagen alpha-1(I) chain
3011.39	29.75	2.63E-04	0.59664	1.18E-11	0.5752	LTGSpGSpGpDGKTGPPGPAGQDGRPGPpGppG	Collagen alpha-1(I) chain
3264.56	25.75	4.90E-02	0.66949	2.30E-04	0.7512	AAGEPGkAGERGVpGPpGAVGPAGKDGEAGAQGPPGP	Collagen alpha-1(I) chain
3295.53	25.45	5.57E-05	0.2414	1.60E-04	0.3386	DRGETGPAGPpGApGAPGAPGPVGpAGKSGDRGETGP	Collagen alpha-1(I) chain
1128.39	33.59	2.29E-03	2.54104	4.36E-08	0.4147	DFDDFNLED	CD99 antigen-like protein 2
2256.97	33.55	6.15E-03	0.49781	3.08E-03	0.8933	ATNSTAGYSIYGVGSmSRYEQ	Calsyntenin-2

Given are molecular mass (in Da), normalized migration time (in min), adjusted p-value (Benjamini and Hochberg) and regulation factor (mean signal intensity of ADPKD samples divided by mean signal intensity of control samples) for training- and test set, amino acid sequence (modified amino acids: p = hydroxyproline; k = hydroxylysine; m = oxidized methionine) and parental protein name.

It has been suggested that in ADPKD, signaling pathways of tubular cell injury and repair are inadequately activated [21]. Several acute kidney injury (AKI) and tubular injury markers, such as NGAL [22] and KIM-1 [23], [24] have been found to be elevated in ADPKD. We therefore tested whether urinary proteomic changes in ADPKD overlap with changes found in AKI. In fact, of the 209 urinary peptides that were altered in ADPKD and have been sequenced, 40 overlapped with peptide fragments that were altered in acute kidney injury (AKI) patients [25] and in 17 of these, one of the two (N- or C-terminal) cleavage sites was identical to the AKI peptides: 13 collagen alpha-1(I), 1 albumin and 3 fibrinogen alpha fragments. When testing the ADPKD urines with a CE-MS based biomarker model that has been developed to detect AKI [25], 112 of all 292 ADPKD patients (38.4%) scored positive, hence ADPKD patients show considerable signs of acute kidney injury in their urinary peptidome. In contrast, when applying the ADPKD_142 biomarker model to 38 urine samples of 16 patients with AKI, none of the AKI urines scored positive for ADPKD. This suggests that the ADPKD_142 biomarker model contains additional markers that are specific for ADPKD vs. AKI. To further evaluate the specificity of the ADPKD_142 model, we tested a total of 481 patients suffering from a variety of non-cystic renal and systemic diseases. **Table 3** depicts the diagnostic groups and their rates of false positive tests; overall specificity of the model was 90.2%. Hence, the detected proteomic alterations are specific for ADPKD and do not simply reflect renal damage. Finally, combining all validation cohorts described above (i.e. all patients that were not used for biomarker discovery: 224 CRISP patients, 27 SUISSE ADPKD patients, 86 healthy controls and 481 diseased controls, total n = 918), yielded an overall sensitivity and specificity for ADPKD of 84.5% and 90.8%, respectively.

**Table 3:** Demographic characteristics and numbers of false positive results in controls with other renal and non-renal diseases.

Diagnosis	N	Number of false positive results	Age (mean ± SD)	Sex (% female)
FSGS	31	2	38.8±11.6	35.4
IgAN	70	9	36.7±12.8	32.9
MN	46	2	44.6±7.9	19.6
MCD	29	2	35.6±12.3	41.4
DNP	83	8	48.6±6.7	26.5
AKI	16	0	61.7±13.3	50.0
Fanconi	11	0	13.4±9.4	36.4
Renal diseases, others	10	0	48.9±7.7	40.0
DM type 1 without DNP	42	7	40.9±10.3	45.2
DM type 2 without DNP	12	0	49.4±9.1	25.0
SLE	45	6	38.7±8.8	71.1
Vasculitis	12	1	42.6±15.2	41.7
Bladder cancer	22	1	51.1±6.2	9.1
Liver transplantation	6	0	45.7±13.2	0
Stem cell transplantation	46	9	50.6±13.5	37.0
All diseased controls combined	481	47	42.7±11.7	35.6

FSGS, focal and segmental glomerulosclerosis; IgAN, IgA nephropathy; MN, membranous nephropathy; MCD, minimal change disease; DNP, diabetic nephropathy; AKI, acute kidney injury; DM, diabetes mellitus; SLE, systemic lupus erythematosus.

The sensitivity and specificity of the diagnostic ultrasound criteria depend on age and genotype, with sensitivity being reduced in young patients and patients with PKD2 genotype [10]. The accuracy of the ADPKD_142 urinary biomarker model exhibited a similar dependence on age and genotype (**Table 4**): sensitivity was lower in young patients and in PKD2 genotype. In the subgroup of patients with PKD1 genotype aged ≥20 years, the model achieved a sensitivity of 91.9% and specificity of 93.0%.

**Table 4:** Sensitivity and specificity of the ADPKD biomarker model according to age and genotype subgroup and using three different diagnostic cut off values.

	all patients		age<30		age>30		PKD1		PKD2	
cut off	sens	spec	sens	spec	sens	spec	sens	spec	sens	spec
−0.169	0.844	0.942	0.720	0.975	0.920	0.913	0.863	0.942	0.774	0.942
−0.250	0.875	0.907	0.768	0.950	0.937	0.870	0.891	0.907	0.806	0.907
−0.400	0.906	0.895	0.817	0.925	0.958	0.870	0.914	0.895	0.903	0.895

**Figure 4:**
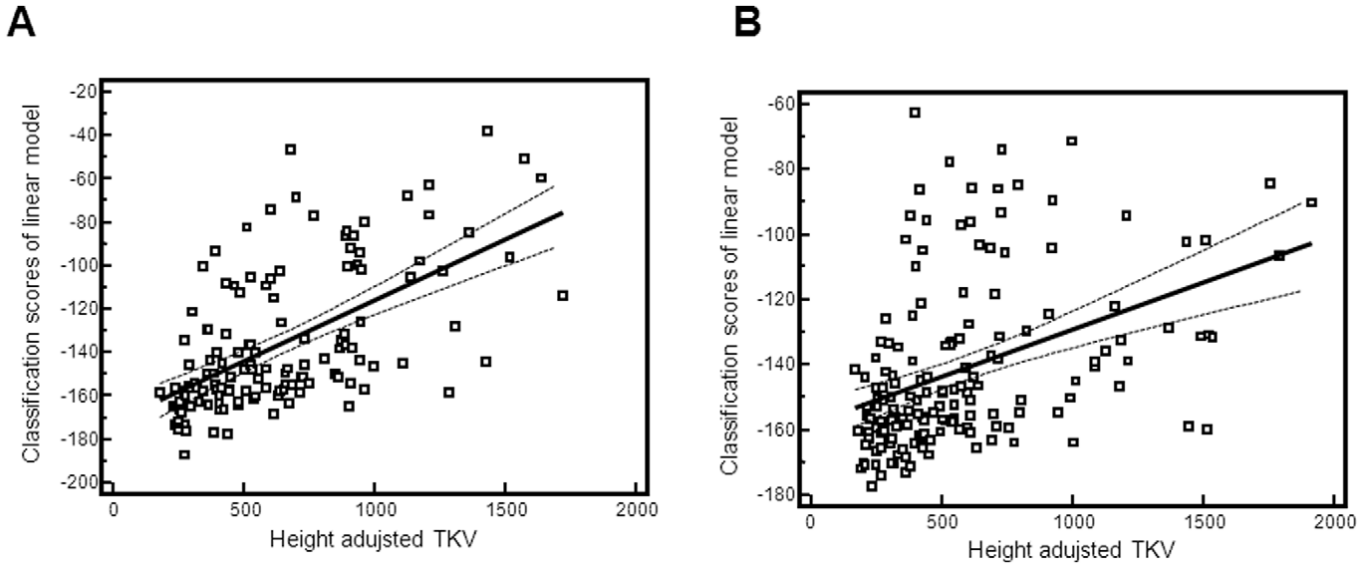
Scatter plots for correlation between classification scores of linear model for disease progression and the height adjusted TKV: Depicted are also the regression line and 95% confidences. In A training set data are showed and in B test set data.

Given the lack of prognostic markers for ADPKD, we next tested whether the urinary proteome of ADPKD patients might reflect disease severity and progression. Since the ADPKD_142 model was generated to distinguish ADPKD from healthy controls with optimal accuracy, the diagnostic score is not expected to correlate well with disease severity. Nevertheless, the ADPKD_142 score correlated positively with total kidney volume (TKV), height adjusted total kidney volume (htTKV) and absolute annual TKV growth (ml per year) and negatively with GFR (**Table 5**), but these correlations were weak. No correlation was found with proteinuria and albuminuria. Since proteomic markers that correlate highly with disease severity may have been excluded from the diagnostic model due to their large variability within ADPKD patients, we next tested the abundance of all 5352 urinary peptides detectable in ADPKD samples for correlation with htTKV, which has been shown to be predictive of future GFR decline and the development of CKD stage III [26]. The analysis was done in a randomly chosen set of 134 patients and validated in a set of 158 patients derived from both ADPKD cohorts. 99 peptides showed a correlation (Spearman's r) of >0.25/<−0.25 with htTKV (**Table S2**). Aiming at a classifier that has superior value in comparison to a single biomarker, we combined all 99 peptides in a linear model. When examining this linear model, the correlation with htTKV was 0.590 (p<0.0001) in the dataset that was used to identify these biomarkers and 0.415 (p<0.0001) in the independent validation set of 158 patients (**Figure 4**). 43 of the 99 peptides could be identified by tandem MS sequencing (**Table 6**). Clearly prominent is the negative correlation of urinary collagen fragments with htTKV.

**Table 5:** Correlation of the biomarker score with clinical markers of disease severity and progression.

Clinical parameter	Spearman's rho	p-value
TKV	0.308	<0.001
TKV/height	0.310	<0.001
TKV change (ml per year)	0.225	0.001
TKV change (% per year)	0.098	0.134
MDRD GFR	−0.284	<0.001
iothalamate GFR	−0.188	0.005
Proteinuria	−0.029	0.698
Albuminuria	0.060	0.389

TKV, total kidney volume.

**Table 6:** Identified 54 biomarkers of the 99 biomarkers that correlated with height adjusted TKV.

Mass (Da)	CE-Time (min)	Spearman's rho	p-values	Sequence	Protein name
840.41	23.17	−0.258	1.76E-06	KGDTGPpGP	Collagen alpha-1(III) chain
858.39	23.24	−0.273	9.08E-06	SpGEAGRpG	Collagen alpha-1(I) chain
935.45	23.68	−0.253	2.83E-05	GRpGPpGPpG	Collagen alpha-1(I) chain
1040.48	25.05	−0.312	7.92E-05	SpGPDGKTGPp	Collagen alpha-1(I) chain
1050.48	26.92	−0.280	1.34E-04	MGPRGPpGPpG	Collagen alpha-1(I) chain
1058.48	24.89	0.324	1.46E-04	TISRLEPED	Ig kappa chain V-III region NG9
1096.48	26.08	−0.325	1.66E-04	ApGDRGEpGpP	Collagen alpha-1(I) chain
1097.50	21.00	0.301	1.67E-04	AHVDDmPNAL	Hemoglobin subunit alpha
1114.49	25.55	−0.288	2.09E-04	SpGERGETGPp	Collagen alpha-1(III) chain
1189.60	21.18	0.271	2.60E-04	YGRAPQLRET	Alpha-1-microglobulin
1223.57	19.39	0.287	2.66E-04	DHEGTHSTKRG	Fibrinogen alpha chain
1251.62	22.53	−0.267	3.10E-04	DGVPGKDGPRGPT	Collagen alpha-1(III) chain
1257.64	19.92	0.346	3.15E-04	TISEKTSDQIH	Antithrombin-III
1265.59	27.09	−0.281	3.33E-04	SpGPDGKTGPpGPA	Collagen alpha-1(I) chain
1268.57	27.25	0.321	3.66E-04	SpGERGETGPpGP	Collagen alpha-1(III) chain
1378.61	28.82	−0.373	7.31E-04	ApGEDGRpGPpGPQ	Collagen alpha-1(II) chain
1430.65	29.24	0.267	9.27E-04	DSEETRAAAPQAW	Drebrin
1447.70	19.47	−0.254	9.49E-04	DTDRFSSHVGGTLG	Inter-alpha-trypsin inhibitor heavy chain H4
1467.66	29.07	0.275	9.97E-04	SpGSpGPDGKTGPpGp	Collagen alpha-1(I) chain
1525.67	30.39	−0.316	1.04E-03	YKTTPPVLDSDGSF	Ig gamma-1 chain C region
1591.74	30.39	0.307	1.07E-03	IGPpGPAGApGDKGESGP	Collagen alpha-1(I) chain
1630.74	20.65	−0.266	1.25E-03	EGSpGRDGSpGAKGDRG	Collagen alpha-1(I) chain
1636.86	23.18	0.313	1.36E-03	LSALEEYTKKLNTQ	Apolipoprotein A-I
1680.75	30.03	−0.257	1.46E-03	TGSpGSpGPDGKTGPpGPA	Collagen alpha-1(I) chain
1692.80	30.89	−0.277	1.48E-03	PpGEAGKpGEQGVPGDLG	Collagen alpha-1(I) chain
1734.79	23.58	−0.282	1.57E-03	GppGPPGKNGDDGEAGKPG	Collagen alpha-1(I) chain
1767.00	24.11	0.321	1.59E-03	SVIDQSRVLNLGPITR	Uromodulin
1796.75	29.45	−0.272	1.60E-03	GEpGApGSKGDTGAKGEpGP	Collagen alpha-1(I) chain
1837.80	30.56	−0.274	1.63E-03	AVAHVDDMPNALSALSDL	Hemoglobin subunit alpha
1847.89	43.67	−0.253	1.65E-03	DAGPVGPpGPpGPpGPPGPPS	Collagen alpha-1(I) chain
1859.83	24.41	−0.266	1.68E-03	NSGEpGApGSKGDTGAKGEp	Collagen alpha-1(I) chain
1860.83	21.40	−0.284	1.84E-03	EGSpGRDGSpGAKGDRGET	Collagen alpha-1(I) chain
1916.85	24.63	−0.258	1.94E-03	GNSGEPGApGSkGDTGAKGEp	Collagen alpha-1(I) chain
1954.97	25.36	0.333	2.25E-03	SHTSDSDVPSGVTEVVVKL	Clusterin
2042.07	25.14	0.295	2.27E-03	EAIPMSIPPEVKFNKPFV	Alpha-1-antitrypsin
2059.01	33.08	0.281	2.31E-03	ELTETGVEAAAASAISVARTL	Plasma protease C1 inhibitor
2080.94	20.20	−0.266	2.36E-03	DAHKSEVAHRFKDLGEEN	Serum albumin
2389.24	22.40	0.260	2.63E-03	MIEQNTKSPLFMGKVVNPTQK	Alpha-1-antitrypsin
2391.20	22.62	0.262	2.70E-03	AAHLPAEFTPAVHASLDKFLASV	Hemoglobin subunit alpha
2405.22	22.47	0.273	2.70E-03	MIEQNTKSPLFmGKVVNPTQK	Alpha-1-antitrypsin
3092.46	31.25	−0.363	2.93E-03	ADGQPGAkGEPGDAGAKGDAGPPGPAGpAGpPGPIG	Collagen alpha-1(I) chain
3108.45	31.28	−0.312	2.95E-03	ADGQpGAKGEpGDAGAKGDAGpPGPAGPAGPPGpIG	Collagen alpha-1(I) chain
3149.46	31.25	−0.259	3.00E-03	GADGQPGAKGEpGDAGAKGDAGPpGPAGpAGPPGPIG	Collagen alpha-1(I) chain

Given are molecular mass (in Da), normalized migration time (in min), the Spearman's coefficient of rank correlation and the significance level (p-values). In addition, amino acid sequence (modified amino acids: p = hydroxyproline; k = hydroxylysine; m = oxidized methionine) and parent protein names are given.

## Discussion

This to the best of our knowledge the largest clinical proteomic study reported so far. We analyzed urine samples from a total of 1,048 patients to characterize the urinary peptidomic pattern of patients with relatively early disease stages of ADPKD. Compared to our initial report [18], we have identified a large number of additional peptides altered specifically in ADPKD and now provide extensive validation in an independent, large and well characterized ADPKD cohort (the CRISP cohort). Insights into the pathways of the proteomic patterns are now becoming clearer and specific proteomic markers appear to associate with disease severity.

Sequencing of naturally occurring peptides still represents a major challenge that frequently cannot be solved successfully [27], [28]. Nevertheless, we were able to identify over 200 peptides associated with ADPKD in the training cohort. This vast number of potential biomarkers is certainly to some degree representative of the disease, enabling the generation of initial hypotheses linking these biomarkers to pathophysiology. Interestingly, the proteomic pattern of ADPKD showed some overlap with proteomic changes during AKI, supporting the hypothesis that some of the pathways driving cyst growth in ADPKD are mechanisms normally active during acute kidney injury repair [21]. Even though individual peptides demonstrated overlap between ADPKD and AKI, the biomarker model was highly specific for ADPKD, as compared to other renal diseases, including AKI, and the overall pattern of peptidomic alterations confers specificity for ADPKD, hence underscoring the advantage of the SVM-based approach to integrate a high number of individual markers with low specificity into a highly specific multidimensional model.

We observed the most prominent proteomic changes in collagen-derived peptides, which represent the majority of the identified biomarkers for ADPKD in this study. The formation of cysts mandates reorganisation of ECM and the increase in tissue collagen required for cyst growth may result in reductions in collagen degradation products. In a recent manuscript, regulation of collagen expression by PKD1 and PKD2 was described, arguing for a negative feedback provided by the polycystin proteins [29]. This is exactly what we observed: a large number of urinary collagen fragments are altered in ADPKD and most of these (about 80%) are in fact down-regulated. In addition, with one exception, all collagen fragments that significantly associated with htTKV are negatively correlated: increasing htTKV (hence severity of disease) is reflected by reduced excretion of specific urinary collagen fragments. We also observed consistent upregulation of peptide fragments from a specific region of fibrinogen alpha chain and of keratin in ADPKD. While the pathophysiological relevance of these findings are not obvious yet, over-expression of genes encoding keratin 19 and fibronectin has been associated with accelerated renal cystogenesis in a mouse PKD model [30] and upregulation of keratin 19 and 2 was associated with ADPKD in a gene profiling study [31]. We further observed consistent downregulation of c-terminal fragments of uromodulin associated with ADPKD, which may be a result of reduced uromodulin degradation. Uromodulin staining was reported to be clearly present in cysts of ADPKD patients [32], indicating reduced degradation, in line with our findings. Osteopontin was reported to be increased in animal models of ADPKD [33] and the reduced excretion of an osteopontin fragment in urine in this study may indicate reduced degradation leading to tissue accumulation.

From a pathophysiological point of view, it is remarkable that a model derived from a cohort primarily consisting of PKD1 patients (although not genotyped, most patients of the SUISSE ADPKD study are expected to have the PKD1 genotype) still positively diagnosed most (77.4%) of the PKD2 patients. This suggest that the majority of biomarkers identified and utilized in the classifier reflect ongoing tissue remodeling that occurs in ADPKD independent of genotype. Importantly, the model did not merely reflect any kind of renal damage, given its remarkable specificity for ADPKD vs. other renal diseases. The direct comparison of PKD1 and PKD2 patients as well as patients with other cystic renal diseases may allow the identification of genotype-specific markers that might be more closely linked to early disease-initiating processes. However, such studies will require substantially larger cohorts, as these likely more subtle changes mandate larger number of samples to be included.

In the majority of cases, the diagnosis of ADPKD is relatively straight forward using ultrasound imaging. Renal ultrasound reaches a very high accuracy in patients with PKD1 genotype aged >30 years [10], and is therefore unlikely to be outreached by alternate diagnostic methods. However, imaging-based diagnosis of ADPKD has limited sensitivity in young patients, particularly those with a PKD2 genotype [10]. We therefore wondered whether urinary proteomics might be useful for ADPKD diagnosis in this patient group. However, similar to the accuracy of ultrasound diagnostic criteria [10] the diagnostic biomarker model exhibited a reduced sensitivity in young patients and in patients with PKD2 genotype and a slightly reduced specificity in older patients. Since for all patients in the validation cohort (the CRISP cohort) ADPKD diagnosis was based on ultrasound imaging, the sensitivity of our proteomic biomarker model might be somewhat lower when applied to an at-risk population, including patients very early in the course with genetically proven disease but negative imaging results. Hence, despite the very high overall accuracy of our diagnostic biomarker model, it will need further refinement before providing benefit over ultrasound based diagnosis in clinical practice. Urine proteome analysis of very young, mutation positive ADPKD patients with no detectable cysts yet might allow the identification of very early and subtle proteomic alterations that may have gone undetected in our study.

A major challenge in the management of patients with ADPKD is to predict prognosis. Even within a family the disease course exhibits a high variability [4]. Disease prediction will gain further importance with the development of specific treatment options. Such treatments will most likely need to be started early during disease course to affect outcome, before the majority of functioning kidney tissue has been replaced by cysts. One focus of our studies was therefore the evaluation of urine proteome utility in predicting severity and progression of ADPKD. We anticipated that the diagnostic biomarker score would not exhibit strong associations with disease severity and progression, since it was designed to discriminate ADPKD patients from controls with high accuracy, but not to detect differences among ADPKD patients. Urinary peptides with highly variable excretion among ADPKD patients that might correlate with disease severity may have been excluded from the diagnostic model since they are less useful to differentiate ADPKD versus controls. Nevertheless, ADPKD_142 correlated with several measures of disease severity and progression, including the annual TKV growth, although these correlations were moderate. We therefore developed a linear model that was specifically designed to correlate with ADPKD severity. A shortcoming of such efforts is the absence of a clear measure for disease progression. Future development of ESRD would likely be the best variable, but this was not available for most patients, as it would require an unfeasibly long observation time for patients with early disease. We therefore chose as a surrogate marker htTKV, which has recently been shown to be a strong predictor of the development of KDOQI CKD Stage 3 and 4 within 8 years in ADPKD patients [26]. A linear model to predict htTKV achieved a high accuracy. This clearly shows, that a subset of proteomic markers different from the diagnostic peptides reflect disease severity. The CRISP and SUISSE studies continue to follow-up data on these patients, including GFR, which will, in the future, serve to validate the current model as a predictive tool and may allow the derivation of a biomarker model that directly predicts TKV growth and GFR decline over time.

Several potential urinary and plasma biomarkers for ADPKD have recently been reported, including NGAL [22], MCP-1 [24], [34], KIM-1 [23], [24], CD-14 and copeptin [35]. These markers, however, are all unspecific for ADPKD and mostly show considerable overlap with healthy controls. Copeptin, CD14 and NGAL correlated with disease severity in the initial reports, however, in the case of NGAL, this could not be confirmed in a subsequent study [36]. The other markers mostly still lack independent validation. Gronwald et al. [37] recently used a metabolomic approach based on NMR spectroscopy of urine and, similar to our approach, combined multiple markers through an SVM algorithm. Although lacking validation in an independent cohort, their model achieved an AUC of 0.91 for the discrimination of ADPKD from normal controls upon nested cross-validation. Like our study, this report demonstrates the potential usefulness of multidimensional profiling of biological fluids to detect biomarker patterns rather than individual markers. On the other hand, the application of “omic” approaches to biomarker discovery is inherently susceptible to overestimating the significance of the findings due to multiple testing, and to model over-fitting when combining biomarkers to classifiers. We have therefore extensively validated our proteomic biomarker model for ADPKD by testing it in the CRISP cohort, a large prospective ongoing ADPKD registry where information on the PKD genotype was available, and in a large group of healthy and diseased controls.

In summary, our study demonstrates that the urine proteome is profoundly altered in young ADPKD patients and that proteomic profiling can be used to derive diagnostic and prognostic models for ADPKD. Further refinement of the presented models will be necessary for future clinical application.

## Methods

### Patients and Procedures

All analyzed urines were morning spot urine samples drawn after the first morning void. ADPKD samples were from baseline visits of two clinical studies: the SUISSE ADPKD study (68 urine samples) and the CRISP cohort (224 urine samples; since all urine samples from the SUISSE ADPKD study were from Caucasian patients, we excluded African American CRISP participants from analysis). Both studies were described in detail elsewhere [38], [39], [40], [41], [42]. Shortly, the SUISSE ADPKD study was an open-label randomized, controlled trial evaluating the effect of sirolimus treatment on kidney volume growth in ADPKD patients aged 18 to 40 years with a creatinine clearance ≥70 ml/min. Patients underwent magnetic resonance imaging (MRI) of their kidneys at 6 months intervals and kidney volumes were determined by a manual segmentation method. The CRISP study was an observational longitudinal study including ADPKD patients aged 16 to 45 years with a creatinine clearance ≥70 ml/min. All patients underwent MRI of their kidneys at annual intervals and kidney volumes were determined by stereology. The total follow up time was 3 years. TKV growth rate was calculated for all patients from both studies as absolute progression rate in ml per year, and as relative growth rate in percent per year by regressing either TKV or log-transformed TKV over time. From the SUISSE ADPKD study, only patients which did not receive sirolimus treatment with at least 4 sequential MRI kidney volume measurements available (N = 48) were used to calculate TKV progression. We used urine samples from the first 41 patients that had been enrolled in the SUISSE ADPKD study and that have been previously analyzed in our first report on urine proteomics in ADPKD [18] as training samples for the refined diagnostic biomarker model and the remaining SUISSE ADPKD urine samples as a second validation cohort (in addition to the CRISP cohort, to test for center bias). Control urine samples have been previously collected as part of several clinical studies (refs [13], [18], [25], [43], [44], [45], [46], [47] and as yet unpublished studies). Demographic characteristics of controls with other renal and non-renal diseases are given in **Table 3**. Healthy control urine samples were collected from volunteers that did not report any history of renal or chronic extrarenal diseases. Mean age of healthy controls was 37±15 years, 49% were females. Out of the healthy control urine samples we randomly chose 2/3 of all samples for biomarker identification and model generation and used the remaining samples as part of the independent validation cohort. Informed consent was obtained from all patients and healthy controls after local ethics committee approval. These studies were performed in accordance with the Helsinki Declaration.

### Sample preparation and CE-MS analysis

All urine samples for CE-MS analyses were stored at −80°C until analysis and underwent a maximum of 2 freeze/thaw cycles. CE-MS analysis was performed exactly as described previously [48]. Briefly, an aliquot was thawed immediately before use, 1∶1 diluted with 2 M urea, 10 mM NH_4_OH, 0.02% SDS, filtered using Centrisart ultracentrifugation filter devices (20 kDa MWCO; Sartorius, Goettingen, Germany) to remove higher molecular weight proteins, desalted on a PD-10 desalting column (Amersham Bioscience, Uppsala, Sweden), equilibrated in 0.01% NH_4_OH in HPLC-grade H_2_O, lyophilized, stored at 4°C, and resuspended in HPLC-grade H_2_O shortly before CE-MS analysis. CE-MS analysis was performed using a P/ACE MDQ capillary electrophoresis system (Beckman Coulter, Fullerton, USA) on-line coupled to a Micro-TOF MS (Bruker Daltonic, Bremen, Germany) as described [48].

### Proteomic data processing and cluster analysis

MosaiquesVisu software [49] was used to deconvolve mass spectral ion peaks representing identical molecules at different charge states into single masses. Migration time and ion signal intensity were normalized using internal polypeptide standards [50] that are unaffected by any disease state studied to date [51]. All detected polypeptides were deposited in a Microsoft SQL database, allowing comparison of multiple samples (patient groups).

### Statistical methods, definition of biomarkers and sample classification

Statistical calculations were carried out in MedCalc version 8.1.1.0 (MedCalc Software, Mariakerke, Belgium, http://www.medcalc.be). Confidence intervals (95% CI) were estimated based on exact binomial calculations. The reported unadjusted p-values were calculated using the natural logarithm-transformed intensities of the CE-MS spectra and the Gaussian approximation to the t-distribution. Statistical adjustment for multiple testing was performed by the method described by Benjamini and Hochberg [52].

Disease-specific polypeptide patterns were generated using SVM based MosaCluster software [53]. The algorithm has been recently described [54]. Briefly, MosaCluster uses Gaussian basis radial functions (RBF) as kernel function to map the data into the high dimensional feature space, where the separating hyperplane can be defined. Ideally, the hyperplane should separate the subjects into two non-overlapping groups, what is often impossible in reality. The accuracy of an SVM model is largely dependent of the selection of model parameters like cost (C) and kernel width (γ). C controls the trade off between allowing training errors and forcing rigid margins and γ controls the width of SVM kernel. To optimize this parameters gird search method was used: the model was evaluated via cross validation at many points within the gird for each parameter to destine the best possible parameter combination. The calculated scores, based on the amplitude of a set of markers, denote the distance of that sample in an n-dimensional space (every dimension representing the amplitude of one marker and n being the number of markers combined to a model) from an (n-1)-dimensional hyperplane that is designed to separate the cases from controls.

### Sequencing of polypeptides

The urine samples were analysed on a Dionex Ultimate 3000 RSLS nano flow system (Dionex, Camberly UK). The samples (5 µl) were loaded onto a Dionex 100 µm×2 cm×5 µm C18 nano trap column at a flow rate of 5 µl/min in 0.1% formic acid and acetonitrile (98∶2). Once loaded onto the trap column the sample was washed off into an Acclaim PepMap C18 nano column 75 µm×15 cm, at a flowrate of 0.3 µl/min. The trap and nano flow column were maintained at 35 C. The samples were eluted with a gradient of solvent A: 0.1% formic acid versus solvent B: acetonitrile starting at 5% B rising to 50% B over 100 min. The eluant from the column was directed to a Proxeon nano spray ESI source (Thermo Fisher Hemel UK) operating in positive ion mode then into an Orbitrap Velos FTMS. The ionisation voltage was 2.5 kV and the capillary temperature was 200°C. The mass spectrometer was operated in MS/MS mode scanning from 380 to 2000 amu. The top 10 multiply charged ions were selected from each full scan for MS/MS analysis, the fragmentation method was HCD at 35% collision energy. The ions were selected for MS2 using a data dependent method with a repeat count of 1 and repeat and exclusion time of 15 s. Precursor ions with a charge state of 1 were rejected. The resolution of ions in MS1 was 60,000 and 7,500 for HCD MS2. Data files were searched against the IPI human non-redundant database using the Open Mass Spectrometry Search Algorithm (OMSSA, http://pubchem.ncbi.nlm.nih.gov/omssa) and Proteome Discoverer (Thermo), without any enzyme specificity. No fixed modification was selected, and oxidation of methionine and proline were set as variable modifications. Mass error window of 10 ppm and 0.05 Da were allowed for MS and MS/MS, respectively. For further validation of obtained peptide identifications, the strict correlation between peptide charge at pH of 2 and CE-migration time was utilized to minimize false-positive identification rates [55]. Calculated CE-migration time of the sequence candidate based on its peptide sequence was compared to the experimental migration time. Accepted were peptides which were found with both search algorithms (OMSSA and Proteome Discoverer), and a CE-migration time deviation below ±1 min.

## Supporting Information

Table S1 **Characteristics of the 657 peptides with altered excretion in ADPKD.** The peptide identification number in the dataset (Peptid ID), molecular mass (in Da) and normalized migration time (in min) are shown along with the AUC-values, p-values adjusted according to Benjamini-Hochberg and the regulation factor for the comparison of cases with controls for both, the training and the validation cohort. In addition, amino acid sequence (modified amino acids: p = hydroxyproline; k = hydroxylysine; m = oxidized methionine), parent protein name with the position of the first (start) and last (stop) amino acid of the identified peptide within the parent protein, the SwissProt/TrEMBLEentry numbers and accession numbers are given. The first 142 peptides were employed in the diagnostic SVM model.
(PDF)

Table S2 **Characteristics of the 99 biomarkers correlated with height adjusted TKV.** Shown are the peptide identification number in the dataset (Peptid ID), molecular mass (in Da) and normalized migration time (in min). Given are the Sperman's coefficient of rank correlation and the significance level (p-values). In addition, amino acid sequence (modified amino acids: p = hydroxyproline; k = hydroxylysine; m = oxidized methionine), parent protein name with the position of the first (start) and last (stop) amino acid, the SwissProt/TrEMBLEentry numbers and accession numbers are given.
(PDF)
