# Correction: Urinary Proteomic Biomarkers for Diagnosis and Risk Stratification of Autosomal Dominant Polycystic Kidney Disease: A Multicentric Study

**DOI:** 10.1371/annotation/9281c713-d253-4a1a-8255-92e691e77a24

**Published:** 2013-08-06

**Authors:** Andreas D. Kistler, Andreas L. Serra, Justyna Siwy, Diane Poster, Fabienne Krauer, Vicente E. Torres, Michal Mrug, Jared J. Grantham, Kyongtae T. Bae, James E. Bost, William Mullen, Rudolf P. Wüthrich, Harald Mischak, Arlene B. Chapman

The legend for Table 6 should read: "Table 6. Identified 43 biomarkers of the 99 biomarkers that correlated with height adjusted TKV."

There were errors in Table 6 and Supplementary Table 2. Correct versions of these tables are available below:

Table 6: 

**Figure:**
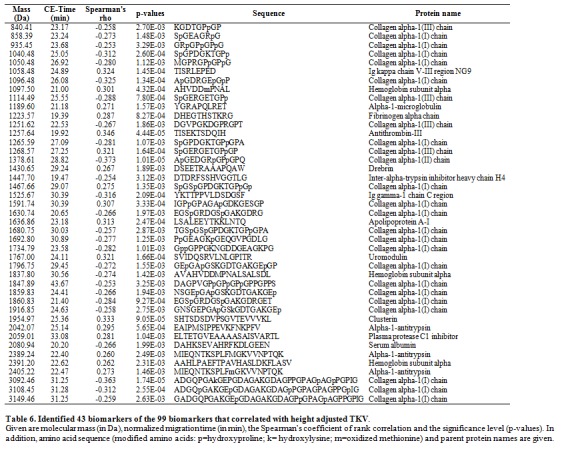


Supplementary Table 2: 
