# Supplementary material for: Urinary Proteomic Biomarkers for Diagnosis and Risk Stratification of Autosomal Dominant Polycystic Kidney Disease: A Multicentric Study
Source: PLoS One. 2013 Jan 10;8(1):e53016. doi: 10.1371/journal.pone.0053016 (PMC3542378; doi:10.1371/journal.pone.0053016)
Supplement: Table S1 — Characteristics of the 657 peptides with altered excretion in ADPKD. The peptide identification number in the dataset (Peptid ID), molecular mass (in Da) and normalized migration time (in min) are shown along with the AUC-values, p-values adjusted according to Benjamini-Hochberg and the regulation factor for the comparison of cases with controls for both, the training and the validation cohort. In addition, amino acid sequence (modified amino acids: p = hydroxyproline; k = hydroxylysine; m = oxidized methionine), parent protein name with the position of the first (start) and last (stop) amino acid of the identified peptide within the parent protein, the SwissProt/TrEMBLEentry numbers and accession numbers are given. The first 142 peptides were employed in the diagnostic SVM model. (PDF) [file pone.0053016.s001.pdf]

Supplementary Table 1:

		Trainingset					Testset										
Mass (Da)	CE-Time	AUC	p-value (BH)	Mean ampl. ADPKD	Mean ampl. Control	fold change	AUC	BH	Mean ADPKD	Mean_NK	fold change	Sequence	Protein name	Start AA	Stop AA	UniProt entry name	Accession nr
911.43	25.88	0.8013937	1.42E-04	500.211	1294.38	0.3864	0.7400332	1.33E-09	256.7726	737.0372	0.3484	DGKTGPpGPA	Collagen alpha-1(I) chain	550	559	CO1A1_HUMAN	gi124056487
923.42	22.01	0.7059233	3.92E-03	74.2014	27.638	2.6848	0.8632683	5.64E-22	134.7256	29.6992	4.5363						
944.51	21.25	0.7996516	1.22E-04	529.2496	138.9	3.8103	0.8046615	6.06E-15	609.1106	174.78	3.4850						
980.5	22.41	0.7595819	5.38E-04	26.8509	137.4204	0.1954	0.572311	7.75E-02	55.9818	50.0898	1.1176						
984.46	24.92	0.8020906	6.56E-05	119.6696	19.4358	6.1572	0.7755918	1.03E-12	109.0746	48.5894	2.2448	LAADDPEVR	Ephrin-A1	166	174	EFNA1_HUMAN	gi73920206
1013.37	25.17	0.769338	4.51E-04	3418.89	1381.9978	2.4739	0.554973	2.31E-01	2074.1357	2463.6496	0.8419						
1050.48	26.92	0.7174216	5.68E-03	574.5168	1058.852	0.5426	0.7656769	1.73E-11	318.15	912.0882	0.3488	MGPRGPpGPpG	Collagen alpha-1(I) chain	217	227	CO1A1_HUMAN	gi124056487
1060.38	38.31	0.6432056	1.70E-02	47.9002	2.3386	20.4824	0.7168293	1.49E-08	104.3392	34.9656	2.9841						
1075.49	20.61	0.6797909	1.79E-02	171.598	51.037	3.3622	0.6769622	1.92E-06	127.842	42.4925	3.0086						
1080.5	25.69	0.7163763	4.18E-03	79.7676	221.591	0.3600	0.5722591	7.44E-02	63.531	131.2281	0.4841	ApGDRGEpGPP	Collagen alpha-1(I) chain	798	808	CO1A1_HUMAN	gi124056487
1082.5	23.91	0.7073171	6.22E-03	387.4328	138.32	2.8010	0.6410403	5.15E-04	335.5096	175.1724	1.9153						
1085.47	21.94	0.7703833	6.10E-04	528.6678	276.8766	1.9094	0.8456188	5.71E-19	934.7308	264.6475	3.5320						
1091.48	20.51	0.6348432	4.29E-02	88.8756	19.494	4.5591	0.5823038	2.51E-02	49.732	38.5892	1.2888						
1096.48	26.08	0.7815331	3.79E-04	5057.316	9302.9887	0.5436	0.7480274	4.00E-10	4450.0671	7298.4934	0.6097	ApGDRGEpGpP	Collagen alpha-1(I) chain	798	808	CO1A1_HUMAN	gi124056487
1101.47	22.27	0.7034843	8.59E-04	65.2168	1.7766	36.7088	0.8143688	4.09E-17	164.4252	23.4634	7.0077						
1114.48	24.21	0.830662	1.45E-05	298.6589	45.2478	6.6005	0.7394103	5.90E-10	190.6725	71.8542	2.6536						
1115.5	21.57	0.6912892	9.56E-03	131.2876	213.8165	0.6140	0.5425664	3.07E-01	210.8384	109.803	1.9202						
1128.39	33.59	0.7400697	2.29E-03	2827.741	1112.8271	2.5410	0.7170889	4.36E-08	910.0543	2194.2505	0.4147	DFDDFNLED	CD99 antigen-like protein 2	26	34	C99L2_HUMAN	gi74730601
1140.52	25.39	0.8111498	6.78E-06	9.4152	171.6858	0.0548	0.5831603	2.60E-02	94.5064	145.1112	0.6513	YNKYPDAVAT	Osteopontin	33	42	OSTP_HUMAN	gi129260
1142.55	24.67	0.6857143	3.60E-04	0	49.1693	0.0000	0.5163777	6.44E-01	10.5126	34.1107	0.3082						
1142.56	21.89	0.6397213	2.78E-02	21.7022	74.8501	0.2899	0.8414659	3.93E-19	317.2428	52.3852	6.0560	VSVNERVMPI	Haptoglobin	256	265	HPT_HUMAN	gi:123508
1143.52	36.97	0.7675958	6.47E-04	179.5289	446.9678	0.4017	0.8008461	0.00E+00	56.4529	538.1454	0.1049	GLPGPpGPpGPpG	Collagen alpha-1(I) chain	139	151	CO1A1_HUMAN	gi124056487
1152.48	27.85	0.6794425	2.75E-03	43.3616	3.7386	11.5984	0.7512458	3.60E-12	120.4839	11.756	10.2487						
1157.54	37.44	0.6794425	2.34E-02	1281.192	1896.04	0.6757	0.9173069	0.00E+00	231.2112	1964.5134	0.1177	GPPGPpGppGPPS	Collagen alpha-1(I) chain	1181	1193	CO1A1_HUMAN	gi124056487
1173.53	37.49	0.6665505	3.30E-02	167.1012	305.9604	0.5462	0.7448609	8.89E-14	25.5134	265.8432	0.0960	GPpGPpGPpGPVT	Collagen alpha-1(XVII) chain	1036	1048	COHA1_HUMAN	gi146345399
1200.54	25.03	0.7616725	3.41E-04	3493.3866	465.6994	7.5014	0.7966674	3.54E-15	6537.0816	643.0425	10.1659						
1200.54	24.14	0.8188153	4.49E-05	3412.348	8871.2124	0.3847	0.8111503	0.00E+00	2386.9814	8788.14	0.2716						
1210.39	36.48	0.6721254	2.79E-02	635.319	214.0104	2.9686	0.783586	5.35E-13	1361.4177	408.845	3.3299						
1215.45	26.88	0.766899	7.36E-05	567.2555	40.224	14.1024	0.6376402	1.93E-04	404.86	166.9017	2.4257						
1217.53	35.78	0.9041812	3.75E-07	576.4848	3844.983	0.1499	0.8471761	0.00E+00	553.3028	4106.9581	0.1347						
1231.42	21.51	0.6794425	5.02E-03	196.6086	39.4955	4.9780	0.7156354	6.93E-10	229.0206	78.3552	2.9229						
1247.52	22	0.7735192	5.32E-04	1669.4988	835.59	1.9980	0.7741642	3.77E-12	2056.3488	891.949	2.3055	DKGETGEQGDRG	Collagen alpha-1(I) chain	1095	1106	CO1A1_HUMAN	gi124056487
1257.44	33.92	0.856446	6.78E-06	5316.53	1816.6382	2.9266	0.6273879	2.34E-03	2871.85	3802.2957	0.7553						
1272.23	35.61	0.6341463	1.43E-02	125.6736	4.9716	25.2783	0.6532911	1.30E-06	228.6036	70.773	3.2301						
1276.4	35.92	0.7066202	8.60E-03	5969.895	3329.05	1.7933	0.6134759	7.45E-03	5099.3773	5320.1396	0.9585						
1297.58	27.36	0.7188153	4.84E-03	942.053	1818.0916	0.5182	0.6134759	6.18E-03	1120.5074	1603.2695	0.6989	SpGSpGPDGKTGPp	Collagen alpha-1(I) chain	543	556	CO1A1_HUMAN	gi124056487
1324.59	28.7	0.7853659	6.56E-05	51.901	298.839	0.1737	0.5611503	1.38E-01	151.6524	197.4141	0.7682	TGPGGDKGDTGPpGP	Collagen alpha-1(III) chain	623	637	CO3A1_HUMAN	gi124056490
1326.56	27.11	0.869338	1.03E-06	107.1585	533.624	0.2008	0.6960133	2.13E-09	209.9823	460.2312	0.4563						
1339.6	27.49	0.697561	1.17E-02	164.7683	245.0486	0.6724	0.7150125	6.10E-11	43.1445	174.8834	0.2467	SpGERGETGPpGPA	Collagen alpha-1(III) chain	796	809	CO3A1_HUMAN	gi124056490
1350.57	21.27	0.7219512	1.01E-03	91.5624	9.3436	9.7995	0.5845619	8.66E-03	36.2181	19.581	1.8497						
1354.64	22.14	0.6696864	6.11E-03	71.6516	5.9301	12.0827	0.7102367	1.19E-09	70.7707	19.078	3.7095						
1363.43	36.34	0.6724739	2.96E-02	3038.22	1627.05	1.8673	0.6015885	1.80E-02	2796.6708	2646.87	1.0566						
1364.61	28.76	0.6529617	6.27E-03	2.989	41.2772	0.0724	0.7519985	2.26E-11	327.096	65.113	5.0235						
1409.58	22.04	0.8494774	3.78E-06	11259.62	5228.46	2.1535	0.6673328	4.26E-05	15879.5	8903.8719	1.7834						
1425.59	22.32	0.7986063	9.53E-05	3003.7686	1635.84	1.8362	0.5903499	3.80E-02	4059.3239	2951.7363	1.3752						
1458.63	27.94	0.7662021	2.96E-04	66.3864	212.0914	0.3130	0.6057413	2.66E-04	44.928	127.4625	0.3525	SpGENGApGQmGPRG	Collagen alpha-1(I) chain	291	305	CO1A1_HUMAN	gi124056487
1467.8	23.89	0.6648084	1.90E-02	197.1971	1061.1648	0.1858	0.6235984	2.54E-04	455.6775	939.2963	0.4851						
1469.67	23.69	0.671777	3.04E-02	3165.02	2337.9037	1.3538	0.7790957	1.50E-12	5772.6702	2969.8515	1.9438	DGQPGAKGEpGDAGAK	Collagen alpha-1(I) chain	820	835	CO1A1_HUMAN	gi124056487
1473.63	22.21	0.7351916	3.79E-04	426.6432	41.0283	10.3988	0.6335652	4.25E-04	738.4104	222.3804	3.3205						
1491.74	39.83	0.8442509	1.45E-05	215.6301	677.0988	0.3185	0.7468335	2.70E-10	319.3785	909.5744	0.3511	VGPpGpPGPPGPPGPPS	Collagen alpha-1(I) chain	1177	1193	CO1A1_HUMAN	gi124056487
1496.63	22.34	0.7087108	1.72E-03	99.8529	10.1052	9.8813	0.6633617	1.61E-06	139.6164	32.317	4.3202						
1505.61	28.77	0.71777	3.83E-04	358.4826	9.2418	38.7893	0.6026267	5.74E-03	182.7258	166.6346	1.0966						
1525.58	22.09	0.6494774	4.39E-02	267.4293	79.8608	3.3487	0.778343	1.08E-12	604.5195	247.2454	2.4450						
1560.58	21.77	0.728223	2.48E-03	357.0022	94.396	3.7820	0.7636784	1.57E-11	497.8705	239.3681	2.0799						
1576.67	43.3	0.6839721	3.59E-03	1448.2512	37.7476	38.3667	0.6832953	6.15E-07	2295.837	685.1799	3.3507						
1579.68	20.06	0.7247387	4.33E-03	8018.68	4802.9982	1.6695	0.6930804	1.90E-06	10791.63	7507.033	1.4375						
1580.89	24.85	0.7090592	6.58E-03	1286.7036	381.1485	3.3759	0.747664	3.80E-10	3152.241	1016.5176	3.1010	LEIELQSLLATKHS	Keratin, type I cytoskeletal 25	309	322	K1C25_HUMAN	gi:74723316
1580.88	23.87	0.7404181	1.18E-03	286.076	792.762	0.3609	0.7114306	2.45E-08	492.8044	883.89	0.5575	IDQSRVLNLGPITR	Uromodulin	593	606	UROM_HUMAN	gi137116
1588.71	30.15	0.910453	3.75E-07	965.7508	134.8746	7.1604	0.7120276	1.18E-07	662.013	439.933	1.5048	TGLSMDGGGSPKGDVDP	Sodium/potassium-transporting ATPase subunit gamma	2	18	ATNG_HUMAN	gi20141251
1613.82	23.99	0.6989547	1.13E-02	190.392	302.3886	0.6296	0.53587	4.57E-01	207.7875	224.399	0.9260	VGGGEQPPPAPAPRRE	Xylosyltransferase 1	51	66	XYLT1_HUMAN	gi71164803
1623.73	24.12	0.7142857	5.64E-03	6272.55	4647.0663	1.3498	0.7643532	2.30E-11	8744.7556	5330.7999	1.6404	DGApGKNGERGGpGGpGP	Collagen alpha-1(III) chain	587	604	CO3A1_HUMAN	gi124056490
1635.76	30.34	0.8020906	7.97E-05	1695.053	499.5666	3.3930	0.5408534	3.90E-01	1268.3156	1288.2651	0.9845	FIFPPSDEQLKSGTA	Ig kappa chain C region	8	22	IGKC_HUMAN	gi125145
1640.58	23.24	0.7714286	5.90E-04	6567.32	3492.6836	1.8803	0.6787012	1.15E-05	8639.7201	5945.5859	1.4531						

1680.75	30.03	0.7836237	3.60E-04	2645.8726	1576.176	1.6787	0.6593906	1.02E-04	3631.2687	2783.3212	1.3047	TGSpGSpGPDGKTGPpGPA	Collagen alpha-1(I) chain	541	559	CO1A1_HUMAN	gi124056487
1684.67	31.75	0.7404181	8.38E-04	512.048	735.3084	0.6964	0.8110465	0.00E+00	321.0601	1584.4584	0.2026	EpGSpGENGApGQMGPR	Collagen alpha-1(I) chain	288	304	CO1A1_HUMAN	gi124056487
1684.67	30.66	0.8602787	2.34E-06	2190.0214	304.4122	7.1943	0.8587002	6.48E-21	3429.052	729.4386	4.7009						
1684.71	29.65	0.8648084	1.11E-06	36.7285	317.8474	0.1156	0.7519207	0.00E+00	66.0252	314.6472	0.2098						
1705.73	40.44	0.6585366	2.22E-03	33.9552	0	∞	0.6374066	1.39E-04	149.155	62.8383	2.3736						
1715.98	20.93	0.6662021	1.58E-02	1564.1494	935.8203	1.6714	0.5249689	5.18E-01	443.0098	529.437	0.8368	VRYTKKVPQVSTPTL	Serum albumin	433	447	ALBU_HUMAN	gi113576
1767	24.94	0.6578397	1.81E-02	146.3122	27.67	5.2878	0.730326	2.46E-09	492.3193	89.8188	5.4813						
1769.78	30.98	0.7233449	2.94E-04	252.3836	0.6012	419.7997	0.7061877	4.04E-09	250.07	19.8126	12.6218						
1794.8	23.92	0.7557491	1.11E-03	2572.9287	1603.507	1.6046	0.828696	3.71E-17	3870.5337	1892.9376	2.0447	GNDGApGKNGERGGpGGpGP	Collagen alpha-1(III) chain	585	604	CO3A1_HUMAN	gi124056490
1882.8	20.24	0.7414634	2.22E-03	29549.17	14347.2693	2.0596	0.6550561	1.64E-04	33360.93	23886.4024	1.3966	DEAGSEADHEGTHSTKRG	Fibrinogen alpha chain	605	622	FIBA_HUMAN	gi1706799
1892.77	40.25	0.6264808	2.51E-02	150.3392	6.3342	23.7345	0.5974356	2.03E-03	570.7113	95.0924	6.0016						
1989.88	32.44	0.6864111	1.81E-02	249.1577	415.4424	0.5997	0.6349408	1.12E-03	233.9922	315.3792	0.7419	SNGNpGPpGPSGSpGKDGPpGP	Collagen alpha-1(III) chain	886	907	CO3A1_HUMAN	gi124056490
2014.9	21.91	0.802439	1.42E-04	4236.0108	2116.9959	2.0010	0.8816964	5.82E-23	5610.3498	2353.2879	2.3840	EGSpGRDGSpGAKGDRGETGP	Collagen alpha-1(I) chain	1021	1041	CO1A1_HUMAN	gi124056487
2067.98	32.49	0.656446	4.32E-03	10.5985	32.5748	0.3254	0.5119913	7.09E-01	38.7056	16.982	2.2792						
2112.92	24.35	0.7229965	4.32E-04	44.9673	2.577	17.4495	0.7917359	7.64E-16	119.574	6.9318	17.2501						
2117.03	42	0.6529617	4.25E-02	166.0725	57.9081	2.8679	0.7446532	4.72E-10	486.3159	124.3116	3.9121						
2128.98	26.97	0.8097561	8.32E-05	63.588	219.4738	0.2897	0.5890262	2.51E-02	51.304	95.7376	0.5359	DGKTGpPGPAGQDGRPGPpGppG	Collagen alpha-1(I) chain	550	572	CO1A1_HUMAN	gi124056487
2137.94	21.79	0.7198606	4.48E-03	1330.0462	786.02	1.6921	0.7290542	9.63E-09	1446.1993	828.1273	1.7463	NGEpGGKGERGApGEKGEGGpPG	Collagen alpha-1(III) chain	818	840	CO3A1_HUMAN	gi124056490
2210.95	33.61	0.6675958	3.36E-02	6592.4012	4329.45	1.5227	0.7368667	2.69E-09	8085.343	5536.02	1.4605	NGApGNDGAKGDAGApGApGSQGApG	Collagen alpha-1(I) chain	700	725	CO1A1_HUMAN	gi124056487
2248.99	25.99	0.702439	9.38E-03	22619.528	9949.5102	2.2734	0.6781821	1.09E-05	24676.488	13205.4792	1.8687						
2256.97	33.55	0.7146341	6.15E-03	613.8862	1233.1804	0.4978	0.6200945	3.08E-03	616.602	690.2496	0.8933	ATNSTAGYSIYGVGSmSRYEQ	Calsyntenin-2	727	747	CSTN2_HUMAN	gi296434469
2264.03	22.67	0.6891986	1.62E-02	572.0028	216.7628	2.6388	0.5921927	3.24E-02	445.5837	310.2918	1.4360	KGDAGApGApGGKGDAGApGERGPpG	Collagen alpha-1(III) chain	662	687	CO3A1_HUMAN	gi124056490
2298.99	27.08	0.641115	1.87E-02	217.0383	12.5972	17.2291	0.6485154	7.61E-05	682.8536	171.346	3.9852						
2335.96	33.7	0.6675958	2.86E-03	407.2738	30.5673	13.3238	0.627336	7.79E-05	129.2952	59.052	2.1895						
2383.66	35.56	0.7651568	1.68E-05	0.4144	100.8234	0.0041	0.6715635	8.17E-12	5.6742	54.9644	0.1032						
2385.05	33.95	0.8139373	7.97E-05	621.612	1259.05	0.4937	0.6746003	1.73E-05	696.345	877.8352	0.7933						
2407.09	27.67	0.7505226	1.44E-03	620.6024	974.3	0.6370	0.585652	5.06E-02	735.165	775.5926	0.9479	LDGAKGDAGPAGPKGEpGSpGENGApG	Collagen alpha-1(I) chain	273	299	CO1A1_HUMAN	gi124056487
2418.16	34.43	0.6759582	8.86E-03	13.954	45.1062	0.3094	0.5443833	2.30E-01	24.5295	27.9356	0.8781						
2445.1	28.24	0.6832753	2.67E-03	124.7356	24.8466	5.0202	0.5471346	1.99E-01	136.0544	54.537	2.4947	mASDASHALEAALEQMDGIIAGTK	Liprin-beta-2	1	24	LIPB2_HUMAN	gi42558987
2471.16	34.77	0.697561	1.18E-02	401.895	798.7661	0.5031	0.7408119	1.01E-09	316.3941	608.328	0.5201	TGPIGPpGPAGApGDKGESGPSGPAGPTG	Collagen alpha-1(I) chain	766	794	CO1A1_HUMAN	gi124056487
2525.2	27.74	0.6815331	2.14E-02	530.248	1155.4347	0.4589	0.6744186	9.41E-06	342.9	657.433	0.5216	LRGGAGPpGPEGGKGAAGPpGPpGAAGTpG	Collagen alpha-1(III) chain	694	723	CO3A1_HUMAN	gi124056490
2564.15	22.98	0.7498258	1.11E-03	6531.44	3062.96	2.1324	0.7166736	6.52E-08	7497.24	4145.6636	1.8085	GApGQNGEpGGKGERGApGEKGEGGPpG	Collagen alpha-1(III) chain	813	840	CO3A1_HUMAN	gi124056490
2574.01	32.81	0.6439024	3.21E-02	16.4912	45.8101	0.3600	0.5909988	9.97E-03	28.3098	65.752	0.4306						
2580.14	22.98	0.7052265	8.95E-03	1322.86	719.1282	1.8395	0.7746833	3.43E-12	1461.7094	712.621	2.0512	GApGQNGEpGGKGERGApGEkGEGGPpG	Collagen alpha-1(III) chain	813	840	CO3A1_HUMAN	gi124056490
2639.29	21.42	0.715331	5.69E-03	234.0891	559.0086	0.4188	0.5367784	4.26E-01	255.472	287.224	0.8895	KEGGKGPRGETGPAGRpGEVGpPGPpGP	Collagen alpha-1(I) chain	903	930	CO1A1_HUMAN	gi124056487
2666.18	35.03	0.6585366	3.86E-03	180.6658	3.1443	57.4582	0.6397166	4.85E-05	269.083	43.0456	6.2511						
2686.34	29.34	0.7470383	4.98E-04	205.4358	18.768	10.9461	0.6494498	7.66E-05	150.12	49.753	3.0173						
2713.23	29.22	0.6891986	1.37E-02	87.5224	206.6029	0.4236	0.5164556	7.24E-01	65.605	95.197	0.6891	PpGADGQpGAKGEpGDAGAKGDAGPpGPAGP	Collagen alpha-1(I) chain	816	846	CO1A1_HUMAN	gi124056487
2767.32	21.67	0.6759582	2.51E-02	228.106	443.2117	0.5147	0.5700789	9.78E-02	201.8187	275.5928	0.7323	KEGGKGPRGETGPAGRpGEVGpPGPpGPAG	Collagen alpha-1(I) chain	903	932	CO1A1_HUMAN	gi124056487
2823.33	29.12	0.6689895	3.19E-02	444.0955	921.3435	0.4820	0.6329423	1.08E-03	469.86	707.799	0.6638	LRGGAGPpGPEGGKGAAGPpGPpGAAGTPGLQG	Collagen alpha-1(III) chain	694	726	CO3A1_HUMAN	gi124056490
2825.27	24.49	0.774216	3.60E-04	22018.26	15191.47	1.4494	0.7789659	1.53E-12	27478.9944	15545.4852	1.7677	ERGEAGIpGVpGAKGEDGKDGSpGEpGANG	Collagen alpha-1(III) chain	448	477	CO3A1_HUMAN	gi124056490
2889.35	24.08	0.6376307	3.04E-02	124.3448	32.9728	3.7711	0.5795525	4.68E-02	142.67	116.4905	1.2247	NGEAGSAGPpGppGLRGSpGSRGLPGADGRAG	Collagen alpha-2(I) chain	384	415	CO1A2_HUMAN	gi124056488
2942.3	22.23	0.7303136	3.52E-03	5153.84	3523.2328	1.4628	0.6511368	2.46E-04	5640.2208	4325.715	1.3039	ESGREGApGAEGSpGRDGSpGAKGDRGETGP	Collagen alpha-1(I) chain	1011	1041	CO1A1_HUMAN	gi124056487
2989.45	24.43	0.7271777	3.31E-03	132.1596	282.712	0.4675	0.5270972	5.18E-01	122.2865	183.7524	0.6655						
3011.39	29.75	0.7902439	2.63E-04	1581.21	2650.1932	0.5966	0.7678052	1.18E-11	1331.5092	2314.7498	0.5752	LTGSpGSpGpDGKTGPPGPAGQDGRPGPpGppG	Collagen alpha-1(I) chain	540	572	CO1A1_HUMAN	gi124056487
3048.39	29.65	0.7114983	4.37E-04	152.5876	2.0352	74.9743	0.6487749	7.16E-05	215.6676	101.9296	2.1158						
3092.44	36.3	0.7006969	1.01E-02	199.5188	452.5664	0.4409	0.6309697	1.13E-03	221.8104	348.6582	0.6362	TGEVGAVGPpGFAGEKGPSGEAGTAGPpGTpGPQG	Collagen alpha-2(I) chain	831	865	CO1A2_HUMAN	gi124056488
3130.39	36.29	0.6707317	1.31E-03	84.9796	0	∞	0.6703956	1.94E-07	108.568	22.197	4.8911						
3137.41	30.35	0.6919861	1.42E-02	367.1148	205.6131	1.7855	0.6386524	6.63E-04	351.491	191.048	1.8398						
3148.28	24.16	0.7052265	4.31E-03	146.7111	37.349	3.9281	0.7065511	4.54E-08	134.7942	67.3585	2.0011						
3166.27	22.06	0.7780488	3.10E-04	572.5902	123.268	4.6451	0.6282184	1.13E-03	349.14	401.6667	0.8692						
3177.42	30.22	0.6682927	5.69E-03	47.4288	7.0173	6.7588	0.6683711	1.31E-06	133.8345	20.9524	6.3875						
3202.43	30.6	0.7902439	4.39E-05	214.8564	17.4658	12.3015	0.593179	4.78E-03	64.992	46.7691	1.3896	SSQGGSLPSEEKGHPQEESEESNVSMASLGE	Secretogranin-1	293	323	SCG1_HUMAN	gi311033509
3255.49	30.78	0.7198606	1.71E-03	191.7779	50.5138	3.7965	0.5845619	2.60E-02	114.8752	67.025	1.7139	NTGApGSpGVSGPKGDAGQpGEKGSpGAQGPPGAPGP	Collagen alpha-1(III) chain	910	946	CO3A1_HUMAN	gi124056490
3258.46	22.92	0.7146341	6.22E-03	2647.1985	1407.8028	1.8804	0.5720775	1.05E-01	2004.4192	1323.3375	1.5147	ENGKPGEpGpKGDAGApGApGGKGDAGApGERGPpG	Collagen alpha-1(III) chain	652	687	CO3A1_HUMAN	gi124056490
3260.39	41.48	0.6498258	2.17E-02	735.4072	113.8269	6.4608	0.63886	2.83E-04	414.5064	192.6876	2.1512						
3264.56	25.75	0.656446	4.90E-02	809.28	1208.8076	0.6695	0.6508254	2.30E-04	697.755	928.879	0.7512	AAGEPGkAGERGVpGPpGAVGPAGKDGEAGAQGPPGP	Collagen alpha-1(I) chain	588	624	CO1A1_HUMAN	gi124056487
3290.5	24.14	0.7554007	8.09E-04	1918.704	352.1254	5.4489	0.6197051	1.52E-03	236.1138	699.1576	0.3377						
3295.53	25.45	0.8181185	5.57E-05	128.8848	533.9138	0.2414	0.6460237	1.60E-04	88.5504	261.5274	0.3386	DRGETGPAGPpGApGAPGAPGPVGpAGKSGDRGETGP	Collagen alpha-1(I) chain	1035	1071	CO1A1_HUMAN	gi124056487
3318.55	30.99	0.7400697	2.27E-03	172.9988	311.6386	0.5551	0.5854963	3.16E-02	86.3612	125.0323	0.6907	GTSLSPPPESSGSPQQPGLSAPHSRQIPAPQGAV	Metastasis-suppressor KiSS-1	68	101	KISS1_HUMAN	gi:292495092
3356.52	25.57	0.6362369	4.09E-02	211.222	46.998	4.4943	0.6257267	4.07E-04	129.3913	43.4299	2.9793						
3375.57	31.92	0.6780488	2.45E-02	1670.9265	2175.3705	0.7681	0.7264846	1.37E-08	825.2685	1499.2136	0.5505						
3385.55	25.49	0.7247387	4.32E-03	2726.2488	4241.4463	0.6428	0.6500467	2.32E-04	1945.895	2774.6712	0.7013						
3426.31	27.7	0.7756098	4.06E-04	224.368	57.1752	3.9242	0.7324024	1.76E-09	192.0834	80.2113	2.3947						
3462.35	19.37	0.6648084	2.14E-02	322.8512	81.6814	3.9526	0.6270505	2.94E-04	141.9306	79.1692	1.7928						
3495.56	31.97	0.6752613	4.12E-03	2196.6611	7.0479	311.6760	0.6245328	1.49E-04	3134.9484	575.5772	5.4466						
3572.6	30.67	0.7616725	5.28E-05	136.4904	0.6588	207.1803	0.6459458	1.42E-05	103.712	82.7216	1.2537						
3718.72	32.48	0.8383275	2.08E-05	926.4288	1719.09	0.5389	0.7763445	1.65E-12	491.9528	1252.9388	0.3926						
3774.81	28.23	0.7261324	6.10E-04	94.0764	612.8868	0.1535	0.5606312	7.53E-02	103.8289	353.7171	0.2935						
3870.81	33.49	0.712892	5.21E-03	70.0281	193.2549	0.3624	0.5894414	2.60E-02	73.7334	79.3555	0.9292						
4005.81	33.06	0.6857143	1.85E-02	634.059	1041.1848	0.6090	0.5622404	1.70E-01	557.0132	640.6875	0.8694						
4100.96	28.78	0.7372822	1.22E-03	45.072	107.5011	0.4193	0.5580876	1.21E-01	33.5823	59.6928	0.5626						

4229	29.17	0.7268293	2.45E-03	125.7149	353.3352	0.3558	0.6532392	6.47E-06	53.8056	248.3494	0.2167						
4251.98	28.76	0.7003484	9.91E-03	704.8812	1393.2996	0.5059	0.7121574	8.61E-08	469.9794	971.34	0.4838						
4671.82	23.28	0.8111498	8.32E-05	540.892	168.6498	3.2072	0.6523048	1.90E-04	402.0378	255.3544	1.5744						
4799.96	23.81	0.9073171	4.74E-07	5469.2428	1379.0854	3.9658	0.7047861	3.69E-07	4094.832	2171.3412	1.8859						
4999.99	24.34	0.7853659	3.36E-04	4240.4466	1400.1213	3.0286	0.5404641	4.02E-01	3188.0699	2582.9343	1.2343						
6169.57	24.77	0.7672474	3.79E-04	1743.5404	189.3845	9.2064	0.7494809	4.23E-11	894.9024	239.1532	3.7420						
9866.54	20.87	0.6731707	2.51E-02	3153.3096	673.692	4.6806	0.6463351	9.96E-07	247.8495	997.9424	0.2484						
801.38	23.08	0.6261324	4.11E-02	12.495	34.048	0.3670	0.5863787	3.44E-03	27.7025	8.2316	3.3654						
801.44	21.8	0.6355401	8.61E-03	2.2845	21.9449	0.1041	0.6233908	2.32E-04	14.4894	10.523	1.3769						
810.4	21.7	0.7285714	5.57E-05	0	36.524	0.0000	0.5282392	1.52E-01	0.662	10.7814	0.0614						
815.44	23.45	0.7571429	1.45E-05	0	439.9974	0.0000	0.5581395	2.86E-06	0	90.9852	0.0000	DSADIPKA	Peptidase inhibitor 15	49	56	PI15_HUMAN	gi74735410
816.38	21.07	0.702439	2.17E-03	11.6331	74.4447	0.1563	0.6476588	5.82E-05	23.5875	25.4562	0.9266						
829.39	23.02	0.6714286	6.10E-04	0	27.4958	0.0000	0.548017	6.35E-03	0.6528	19.292	0.0338						
840.4	25.43	0.6961672	7.95E-03	20.9836	108.216	0.1939	0.5055804	8.97E-01	13.857	41.3588	0.3350						
840.41	23.17	0.7320557	1.61E-03	22.2573	249.6186	0.0892	0.5284468	5.40E-01	38.1292	103.7205	0.3676	KGDTGPpGP	Collagen alpha-1(III) chain	629	637	CO3A1_HUMAN	gi124056490
840.43	19.63	0.6857143	3.60E-04	0	46.6681	0.0000	0.5275125	3.11E-01	6.902	14.254	0.4842						
853.42	22.61	0.6857143	3.60E-04	0	27.4503	0.0000	0.5199855	3.34E-01	0.5556	5.998	0.0926						
855.38	25.37	0.6418118	1.81E-02	11.4108	40.108	0.2845	0.5177274	5.08E-01	2.29	10.4958	0.2182						
858.39	23.24	0.6898955	1.58E-02	180.832	588.6816	0.3072	0.5353509	4.61E-01	196.2085	343.767	0.5708	SpGEAGRpG	Collagen alpha-1(I) chain	522	530	CO1A1_HUMAN	gi124056487
868.41	23.31	0.7254355	3.92E-03	87.4578	308.3542	0.2836	0.5226848	6.47E-01	83.67	140.292	0.5964						
873.4	21.1	0.6759582	2.25E-03	1.0416	24.86	0.0419	0.5973837	2.16E-03	10.434	9.2652	1.1261						
879.5	19.88	0.8233449	3.30E-06	4.725	131.3001	0.0360	0.5315096	4.35E-01	30.6252	40.8044	0.7505						
882.52	23.84	0.7745645	1.66E-05	0.9155	673.0617	0.0014	0.5677949	2.61E-05	0.8088	172.4649	0.0047	LNLGPITR	Uromodulin	599	606	UROM_HUMAN	gi137116
884.29	43.81	0.6195122	4.91E-02	34.0215	21.1354	1.6097	0.6103094	9.31E-04	98.3185	34.4128	2.8570						
897.44	22.3	0.7571429	1.45E-05	0	55.08	0.0000	0.560164	4.78E-03	1.9278	19.3819	0.0995						
922.41	21.94	0.6787456	5.21E-03	21.489	81.6928	0.2630	0.5958264	5.61E-04	8.6242	48.784	0.1768						
927.41	23.03	0.6425087	4.36E-03	1.2104	15.9588	0.0758	0.5095775	7.08E-01	3.036	5.256	0.5776						
935.45	23.68	0.7400697	1.07E-03	34.8432	173.8638	0.2004	0.5942951	6.13E-03	18.7218	97.1454	0.1927	GRpGPpGPpG	Collagen alpha-1(I) chain	563	572	CO1A1_HUMAN	gi124056487
937.46	34.1	0.7121951	2.92E-03	16.5184	79.704	0.2072	0.6050145	2.94E-04	3.5712	47.2668	0.0756						
947.44	24.69	0.6292683	3.23E-02	17.859	82.9096	0.2154	0.5353769	1.85E-01	22.3894	47.5216	0.4711						
949.22	34.33	0.669338	7.19E-03	448.1268	45.0582	9.9455	0.7366071	2.85E-10	627.0095	177.6936	3.5286						
953.47	23.42	0.6857143	3.60E-04	0	25.1489	0.0000	0.5108493	6.47E-01	1.744	4.652	0.3749						
965.18	34.32	0.641115	7.95E-03	248.7968	37.5345	6.6285	0.5150799	5.21E-01	10.2	34.1913	0.2983						
969.41	20.87	0.7930314	4.34E-06	0.906	108.468	0.0084	0.5626298	2.63E-03	1.9914	51.3891	0.0388						
978.47	19.76	0.7714286	6.78E-06	0	75.357	0.0000	0.5211534	5.18E-01	11.2727	66.3926	0.1698	LAADFVESK	Serum albumin	329	337	ALBU_HUMAN	gi113576
981.48	24.42	0.6783972	5.92E-03	46.3197	22.0913	2.0967	0.6363164	1.51E-05	23.409	22.6944	1.0315						
981.59	24.8	0.8811847	2.23E-06	275.6924	1793.0094	0.1538	0.8123702	0.00E+00	188.5964	1104.7632	0.1707	VLNLGPITR	Uromodulin	598	606	UROM_HUMAN	gi137116
984.47	23.06	0.7334495	2.51E-04	3.244	62.067	0.0523	0.5747508	4.65E-02	17.5602	20.2356	0.8678						
988.5	21.25	0.7135889	4.55E-03	174.5796	62.7062	2.7841	0.7025799	1.67E-07	238.9056	68.9588	3.4645	YQTNKAKH	Cystatin-B	85	92	CYTB_HUMAN	gi1235678
998.48	23.23	0.6571429	1.09E-03	0	13.6586	0.0000	0.5217245	2.18E-01	0.6612	4.73	0.1398						
1000.49	22.73	0.6714286	6.10E-04	0	35.6218	0.0000	0.5288881	2.65E-01	3.039	19.0701	0.1594						
1002.45	24.42	0.6543554	4.75E-03	48.3752	6.2226	7.7741	0.5669643	1.77E-03	29.4476	0	∞	DGGLLTGNEK	Keratin, type I cytoskeletal 13	96	105	K1C13_HUMAN	gi269849755
1008.51	22.69	0.6714286	6.10E-04	0	38.2058	0.0000	0.510434	7.32E-01	6.903	24.8292	0.2780						
1016.45	20.31	0.6773519	8.68E-04	0.6054	37.9546	0.0160	0.5066445	8.06E-01	4.467	27.383	0.1631						
1025.47	25	0.6630662	9.54E-03	25.5645	126.9876	0.2013	0.5598526	5.65E-02	28.4373	67.1804	0.4233	ATKTVGSDTF	Kininogen-1	62	71	KNG1_HUMAN	gi124056474
1025.46	25.59	0.6578397	3.98E-02	164.1927	346.2613	0.4742	0.5915698	2.98E-02	206.3256	144.946	1.4235	PpGSAGAPGKDG	Collagen alpha-1(I) chain	1143	1154	CO1A1_HUMAN	gi124056487
1032.5	21.21	0.754007	8.50E-04	860.6116	170.7831	5.0392	0.6724979	1.18E-05	606.7515	354.4044	1.7120						
1040.47	25.05	0.7052265	7.55E-03	171.1962	348.5502	0.4912	0.5529485	2.48E-01	241.095	242.8606	0.9927	SpGPDGKTGPp	Collagen alpha-1(I) chain	546	556	CO1A1_HUMAN	gi124056487
1041.44	20.82	0.6857143	3.60E-04	0	45.2473	0.0000	0.5639535	7.91E-07	0	25.2804	0.0000						
1046.51	25.35	0.6592334	2.51E-02	41.3472	289.764	0.1427	0.520816	6.32E-01	39.59	189.5237	0.2089	SGSVIDQSRV	Uromodulin	589	598	UROM_HUMAN	gi137116
1053.48	25.07	0.6891986	9.56E-03	43.6896	146.4435	0.2983	0.5388808	3.57E-01	73.2333	63.954	1.1451	GpGSDGKPGPpG	Collagen alpha-1(III) chain	544	555	CO3A1_HUMAN	gi124056490
1064.48	21.02	0.615331	3.18E-02	10.8332	17.1678	0.6310	0.564213	3.56E-02	17.1672	12.5796	1.3647						
1065.5	25.45	0.6926829	3.04E-03	8.0495	119.3836	0.0674	0.5676391	3.65E-02	47.2416	66.0314	0.7154	GPDGKTGPpGPA	Collagen alpha-1(I) chain	548	559	CO1A1_HUMAN	gi124056487
1067.49	20.51	0.7142857	1.08E-04	0	29.1755	0.0000	0.5030627	9.10E-01	5.8392	13.1404	0.4444						
1070.49	36.49	0.6529617	2.41E-02	20.3448	109.5199	0.1858	0.6704475	1.49E-10	5.2443	121.6454	0.0431	GPpGpPGPpGPP	Collagen alpha-2(VIII) chain	519	530	CO8A2_HUMAN	gi45644957
1080.48	27.77	0.7247387	3.57E-03	89.0622	242.4268	0.3674	0.6358233	8.11E-05	33.3342	116.6932	0.2857	DRGEpGPpGPA	Collagen alpha-1(I) chain	801	811	CO1A1_HUMAN	gi124056487
1084.51	36.84	0.6905923	1.12E-03	2.2946	44.9823	0.0510	0.591414	1.78E-06	0.8427	44.1153	0.0191	pPGPpGPPGPPS	Collagen alpha-1(I) chain	1182	1193	CO1A1_HUMAN	gi124056487
1085.42	20.77	0.671777	1.15E-02	137.533	499.5054	0.2753	0.6669695	3.33E-13	4.9616	515.856	0.0096						
1098.49	25.23	0.6139373	4.01E-02	4.615	20.4352	0.2258	0.5345203	2.24E-01	6.8471	50.148	0.1365						
1100.5	37.04	0.7393728	1.42E-03	75.2321	196.8659	0.3821	0.7027097	2.16E-09	15.5682	168.9212	0.0922	pGPPGpPGpPSP	Collagen alpha-6(IV) chain	432	443	CO4A6_HUMAN	gi116241307
1107.51	26.84	0.6522648	1.42E-02	11.151	42.828	0.2604	0.606468	2.99E-03	58.4742	19.4986	2.9989	SEPQEVLHIG	PDZ and LIM domain protein 1	108	117	PDLI1_HUMAN	gi:20178312
1113.51	22.26	0.6299652	3.14E-02	138.2727	25.5827	5.4049	0.6293605	4.53E-06	88.2198	0.8749	100.8342						
1116.53	20.85	0.6961672	4.36E-03	86.464	31.2239	2.7692	0.6963767	8.06E-09	82.1698	11.992	6.8521						
1117.51	36.96	0.7317073	1.75E-03	45.3324	168.8085	0.2685	0.6210029	2.66E-04	22.8425	105.524	0.2165	GpAGPpGPpGPpG	Collagen alpha-1(III) chain	174	186	CO3A1_HUMAN	gi124056490
1121.49	27.68	0.7494774	3.61E-04	25.9	171.9207	0.1507	0.5457849	2.15E-01	41.7452	90.4021	0.4618	DGRpGPpGPpGA	Collagen alpha-1(I) chain	562	573	CO1A1_HUMAN	gi124056487
1122.4	25	0.6627178	4.55E-03	104.2734	1.176	88.6679	0.5918293	9.92E-04	64.3586	15.2408	4.2228						
1126.51	25.52	0.7219512	1.54E-03	58.7048	184.6026	0.3180	0.5583991	1.45E-01	146.9776	97.1382	1.5131	GGpGSDGKpGPpG	Collagen alpha-1(III) chain	543	555	CO3A1_HUMAN	gi124056490
1128.39	35.58	0.666899	2.32E-03	7.703	410.0266	0.0188	0.5595671	4.51E-03	10.4826	134.3892	0.0780						
1128.49	25.65	0.6731707	2.73E-02	196.847	312.61	0.6297	0.7362957	5.11E-10	87.7584	262.7343	0.3340						
1132.57	24.04	0.6526132	4.21E-03	1.9155	26.8498	0.0713	0.5854963	7.27E-03	15.9964	7.0104	2.2818	PGKEGPpGPQGP	Collagen alpha-1(XXII) chain	1221	1232	COMA1_HUMAN	gi296434458
1134.58	23.66	0.8505226	5.96E-06	102.058	629.477	0.1621	0.6893688	2.42E-06	190.0224	514.4447	0.3694	PIGQEGAPGRPG	Collagen alpha-2(IV) chain	1463	1474	CO4A2_HUMAN	gi143811377
1135.24	35.22	0.6243902	4.41E-02	17.0119	154.8561	0.1099	0.5324958	1.71E-01	1.5696	110.373	0.0142						
1137.48	28.35	0.6585366	5.69E-03	21.3374	47.216	0.4519	0.544539	1.69E-01	60.1243	25.5208	2.3559	GpmGPRGPpGPA	Collagen alpha-1(II) chain	204	215	CO2A1_HUMAN	gi124056489

1137.51	26.43	0.6466899	4.20E-02	37.6192	84.1035	0.4473	0.5167151	6.66E-01	17.3964	44.322	0.3925	DTNADKQLSF	Protein S100-A9	67	76	S10A9_HUMAN	gi115444
1137.54	23.63	0.6571429	1.09E-03	0	64.1545	0.0000	0.5324958	3.54E-01	21.4082	63.789	0.3356	FQQDKFLGR	Prostaglandin-H2 D-isomerase	34	42	PTGDS_HUMAN	gi:730305
1139.49	20.97	0.661324	1.81E-02	482.262	1134.2862	0.4252	0.6259863	1.06E-03	546.2128	539.665	1.0121						
1140.47	21.07	0.6968641	1.14E-02	1541.6088	685.098	2.2502	0.521387	6.62E-01	1671.6192	1599.829	1.0449						
1154.51	25.65	0.8041812	1.25E-04	345.9568	641.5312	0.5393	0.5666528	1.33E-01	245.7432	346.7796	0.7086	PpGEAGKpGEQG	Collagen alpha-1(I) chain	651	662	CO1A1_HUMAN	gi124056487
1159.6	26.07	0.656446	4.35E-02	711.604	219.4759	3.2423	0.592011	2.94E-02	729.2817	571.3308	1.2765	SGSVIDQSRVL	Uromodulin	589	599	UROM_HUMAN	gi137116
1168.29	35.36	0.6829268	8.50E-04	134.6282	0	∞	0.5780212	4.83E-03	72.5256	13.428	5.4011						
1171.51	29.18	0.6491289	4.83E-02	40.1324	64.6695	0.6206	0.5647321	7.62E-02	20.5349	63.3908	0.3239						
1172.51	37.41	0.6466899	8.85E-03	18.4142	66.0413	0.2788	0.5263704	2.24E-01	4.1538	25.8818	0.1605						
1174.54	38.13	0.6836237	6.26E-03	780.4083	29.158	26.7648	0.5018428	9.49E-01	24.8171	47.12	0.5267	DFSFLPQPPQ	Collagen alpha-1(I) chain	1197	1206	CO1A1_HUMAN	gi124056487
1178.39	20.71	0.6641115	3.09E-02	207.842	103.5913	2.0064	0.7451723	1.17E-10	212.8651	134.5146	1.5825						
1180.52	35.7	0.6954704	1.09E-02	217.62	575.19	0.3783	0.7036441	1.64E-08	111.5748	623.8932	0.1788						
1180.54	25.62	0.6599303	1.51E-02	29.714	67.1937	0.4422	0.5395037	2.74E-01	16.6141	28.0224	0.5929	VAHVDDMPNAL	Hemoglobin subunit alpha	71	81	HBA_HUMAN	gi:57013850
1182.55	28.27	0.7627178	5.97E-04	46.0768	152.224	0.3027	0.6156302	1.57E-03	30.6816	106.1112	0.2891						
1184.56	26.4	0.6682927	2.22E-02	56.8704	87.2781	0.6516	0.5394778	3.61E-01	52.875	74.8414	0.7065	KpGEQGVpGDLG	Collagen alpha-1(I) chain	657	668	CO1A1_HUMAN	gi124056487
1185.56	23.79	0.6989547	5.21E-03	18.1056	76.836	0.2356	0.6184593	1.27E-03	66.861	25.6703	2.6046						
1191.52	36.18	0.7944251	1.50E-04	124.8946	487.3195	0.2563	0.7588248	2.21E-13	114.558	506.3256	0.2263	pPGSNGNpGPpGP	Collagen alpha-1(II) chain	907	919	CO2A1_HUMAN	gi124056489
1211.54	25.82	0.7254355	2.46E-03	85.9904	153.846	0.5589	0.5142753	7.62E-01	67.0036	93.392	0.7174	GPpGEAGKpGEQG	Collagen alpha-1(I) chain	650	662	CO1A1_HUMAN	gi124056487
1220.54	22.75	0.6418118	1.36E-02	6.796	36.9371	0.1840	0.5324439	3.45E-01	22.6488	26.59	0.8518						
1221.58	26.79	0.7878049	1.91E-05	4.601	87.2676	0.0527	0.5256956	5.12E-01	20.1057	29.5864	0.6796						
1235.37	36.11	0.6682927	1.04E-02	83.3085	118.0803	0.7055	0.5974616	5.02E-07	3.0669	79.4948	0.0386						
1238.58	22.46	0.6445993	3.13E-02	23.3354	48.5247	0.4809	0.7896076	2.68E-14	197.925	33.8951	5.8393	SDGLAHLDNLKG	Hemoglobin subunit delta	73	84	HBD_HUMAN	gi122713
1250.64	20.42	0.7456446	3.10E-04	137.04	141.9957	0.9651	0.5511057	2.19E-01	158.3665	104.8572	1.5103	AGVANALAHKYH	Hemoglobin subunit beta	136	147	HBB_HUMAN	gi:56749856
1253.58	25.03	0.6912892	4.98E-04	0.7486	29.296	0.0256	0.5105897	5.97E-01	6.3115	10.4	0.6069	SQGESGRpGPPGp	Collagen alpha-1(III) chain	556	568	CO3A1_HUMAN	gi124056490
1260.56	21.83	0.7519164	4.32E-04	435.7584	77.0338	5.6567	0.7659624	6.41E-12	565.584	174.7764	3.2360						
1262.46	38.23	0.6616725	2.93E-02	367.8237	64.852	5.6717	0.5268376	5.30E-01	148.6623	163.9883	0.9065						
1263.55	19.68	0.6351916	2.78E-02	13.98	56.448	0.2477	0.5326516	2.83E-01	25.6481	36.1074	0.7103						
1263.54	22.73	0.6878049	1.70E-02	714.3224	381.4965	1.8724	0.7105482	1.56E-07	869.3958	406.9334	2.1365						
1265.59	27.09	0.687108	1.77E-02	4809.944	8972.253	0.5361	0.8250363	0.00E+00	2607.3519	7830.2846	0.3330	SpGPDGKTGPpGPA	Collagen alpha-1(I) chain	546	559	CO1A1_HUMAN	gi124056487
1267.61	22.67	0.6571429	1.09E-03	0	26.66	0.0000	0.5096294	7.06E-01	6.576	12.4812	0.5269	DGVpGKDGPRGPT	Collagen alpha-1(III) chain	752	764	CO3A1_HUMAN	gi124056490
1272.56	21.04	0.6773519	8.68E-04	0.633	38.7834	0.0163	0.520245	4.54E-01	8.2621	29.718	0.2780						
1280.59	37.85	0.6710801	2.92E-03	2.1756	33.076	0.0658	0.5709095	3.57E-04	5.9932	22.5962	0.2652	LPGPpGPpGPpGPP	Collagen alpha-1(I) chain	140	153	CO1A1_HUMAN	gi124056487
1281.58	27.09	0.7557491	8.65E-04	177.7581	426.3259	0.4170	0.5820702	4.90E-02	188.904	227.6764	0.8297						
1295.36	34.16	0.6449477	4.66E-02	226.3626	42.4354	5.3343	0.5395297	2.89E-01	71.796	81.108	0.8852						
1296.59	19.36	0.6745645	1.07E-03	3.07	108.7282	0.0282	0.5202969	5.66E-01	33.3058	90.8488	0.3666						
1296.62	25.21	0.6571429	1.09E-03	0	22.3913	0.0000	0.5256956	3.35E-01	4.3593	10.4823	0.4159	IQNWPHYRSP	Peptidoglycan recognition protein 1	187	196	PGRP_HUMAN	gi18202143
1304.52	27.88	0.6682927	1.14E-02	882.1236	13.262	66.5151	0.5001557	9.97E-01	189.3684	182.6776	1.0366						
1304.6	28.16	0.6425087	4.36E-03	4.4708	45.9203	0.0974	0.5296148	4.24E-02	2.6478	23.0967	0.1146						
1310.58	27.01	0.8536585	2.98E-06	45.294	168.0142	0.2696	0.6890054	2.45E-13	23.2267	102.0949	0.2275	SpGGPGSDGKpGPpG	Collagen alpha-1(III) chain	541	555	CO3A1_HUMAN	gi124056490
1312.49	27.8	0.6209059	2.41E-02	76.3931	326.5075	0.2340	0.6021076	6.50E-10	0	198.7232	0.0000						
1321.59	30.02	0.6188153	2.67E-02	1285.305	4191.2837	0.3067	0.520245	4.48E-01	824.805	2512.2199	0.3283						
1321.6	38.58	0.6425087	1.31E-02	15.402	314.5	0.0490	0.6224824	5.16E-06	13.5784	174.8406	0.0777						
1324.58	24.73	0.6167247	4.52E-02	8.4756	33.388	0.2539	0.5649398	6.66E-02	36.7818	36.1582	1.0172						
1326.57	21.67	0.6578397	1.37E-02	95.6032	17.8682	5.3505	0.6442068	1.27E-05	106.2404	31.3191	3.3922						
1328.5	26.78	0.641115	4.72E-02	75.5616	28.7622	2.6271	0.6803104	5.00E-07	41.9526	31.6848	1.3241						
1332.42	36.13	0.6585366	2.22E-03	38.0096	0	#DIV/0!	0.5765158	8.03E-03	29.3457	18.032	1.6274						
1333.57	28.43	0.6808362	7.19E-03	64.2413	73.6725	0.8720	0.5218542	5.28E-01	16.742	38.1225	0.4392	VAMTPRSEGSSVN	Prothrombin	193	205	THRB_HUMAN	gi135807
1335.63	38.71	0.7310105	4.66E-04	9.48	64.1763	0.1477	0.6512666	3.52E-08	10.7723	70.6367	0.1525	PpGPpGPPGPPGPPS	Collagen alpha-1(I) chain	1179	1193	CO1A1_HUMAN	gi124056487
1337.62	38.2	0.695122	8.61E-03	63.4842	157.6581	0.4027	0.6728094	8.74E-08	34.3497	196.4352	0.1749	GLPGPpGPpGPpGPP	Collagen alpha-1(I) chain	139	153	CO1A1_HUMAN	gi124056487
1341.58	29.98	0.6707317	2.60E-02	313.7211	83.4537	3.7592	0.5210756	5.97E-01	49.6248	39.6956	1.2501	SpGEAGRpGEAGLp	Collagen alpha-1(I) chain	522	535	CO1A1_HUMAN	gi124056487
1351.64	38.76	0.7442509	1.60E-03	139.132	365.7063	0.3804	0.7398256	6.10E-11	79.2597	408.485	0.1940	PpGPpGPpGPPGPPS	Collagen alpha-1(I) chain	1179	1193	CO1A1_HUMAN	gi124056487
1353.66	25.63	0.7965157	1.79E-04	212.8456	538.1103	0.3955	0.7276267	1.18E-08	260.2257	559.917	0.4648	KGEAGLpGApGSPGQ	Collagen alpha-1(XIX) chain	291	305	COJA1_HUMAN	gi68840003
1357.58	30.02	0.7707317	5.12E-04	297.336	65.532	4.5373	0.5720775	6.88E-02	79.7364	51.528	1.5474	DGQpGAKGEpGDAGA	Collagen alpha-1(I) chain	820	834	CO1A1_HUMAN	gi124056487
1367.64	38.88	0.687108	1.76E-02	633.5588	1165.0864	0.5438	0.8590376	0.00E+00	278.8443	1294.6752	0.2154	PpGPpGPpGPPGTPV	Collagen alpha-1(XVIII) chain	1256	1270	COIA1_HUMAN	gi45644997
1368.58	21.9	0.8111498	4.70E-06	190.638	0.7887	241.7117	0.6812708	2.69E-07	220.5456	75.2648	2.9303						
1383.64	38.94	0.6982578	4.59E-03	48.2472	156.702	0.3079	0.6259863	8.62E-05	34.1796	113.3781	0.3015						
1396.62	26.67	0.7191638	4.32E-03	64.9689	171.5252	0.3788	0.5416321	3.11E-01	51.6039	80.946	0.6375						
1399.41	36.2	0.6390244	3.38E-02	26.0253	26.6854	0.9753	0.5546615	8.48E-03	1.9212	13.1716	0.1459						
1408.66	39.13	0.7721254	5.31E-04	289.2652	801	0.3611	0.7904641	1.75E-14	251.8431	988.7888	0.2547	GPPGppGPpGPPGPPS	Collagen alpha-1(I) chain	1178	1193	CO1A1_HUMAN	gi124056487
1410.66	22.33	0.6097561	4.83E-02	9.333	35.4702	0.2631	0.5158846	5.27E-01	14.5647	33.5188	0.4345						
1416.65	27.85	0.630662	1.73E-02	7.2009	31.5112	0.2285	0.5404381	5.57E-02	4.4436	23.894	0.1860	GDLSTPDAVmGNPK	Hemoglobin subunit beta	47	60	HBB_HUMAN	gi:56749856
1431.72	19.47	0.6526132	4.21E-03	3.14	61.9412	0.0507	0.5291476	3.55E-01	39.7499	342.6897	0.1160	HAHKLRVDPVNF	Hemoglobin subunit alpha	88	99	HBA_HUMAN	gi:57013850
1434.7	22.58	0.6714286	6.10E-04	0	35.9414	0.0000	0.5728821	2.61E-02	70.6266	25.5088	2.7687						
1436.67	38.92	0.6578397	2.29E-03	1.844	36.057	0.0511	0.5630191	8.46E-04	3.0948	26.4928	0.1168	PGDPGPpGPpGPpGPP	Collagen alpha-1(XV) chain	1092	1107	COFA1_HUMAN	gi68839886
1440.66	39.28	0.7672474	5.70E-04	200.481	547.6652	0.3661	0.7862074	0.00E+00	183.308	682.323	0.2687						
1442.63	27.63	0.6843206	7.30E-03	266.475	19.9272	13.3724	0.6444145	9.67E-06	150.3384	35.8704	4.1912	SpGENGApGQMGPRG	Collagen alpha-1(I) chain	291	305	CO1A1_HUMAN	gi124056487
1444.36	36.37	0.6815331	2.46E-03	1200.562	129.8202	9.2479	0.5074751	8.47E-01	266.8966	273.651	0.9753						
1444.67	20.02	0.6515679	2.87E-02	31.9923	97.6293	0.3277	0.6389379	2.62E-04	111.617	61.3248	1.8201	SpGRDGSpGAKGDRG	Collagen alpha-1(I) chain	1023	1037	CO1A1_HUMAN	gi124056487
1460.64	19.83	0.6226481	4.74E-02	19.3875	69.556	0.2787	0.5942172	9.33E-03	125.2796	88.5216	1.4152						
1463.68	19.47	0.6397213	3.84E-02	57.1714	160.6122	0.3560	0.5078904	8.29E-01	54.408	102.546	0.5306						
1466.65	28.52	0.7979094	1.18E-04	84.6604	431.8158	0.1961	0.5247872	6.16E-01	264.8376	308.113	0.8595						
1467.67	21.29	0.6902439	7.07E-03	53.4238	183.6654	0.2909	0.6204838	3.36E-03	273.7062	230.5162	1.1874						
1470.68	21.08	0.7212544	1.58E-03	20.8252	71.4735	0.2914	0.5605274	1.26E-01	69.212	126.6806	0.5464	DGLAHLDNLKGTFA	Hemoglobin subunit beta	74	87	HBB_HUMAN	gi:56749856

1474.64	28.94	0.6919861	4.74E-04	3.8058	162.12	0.0235	0.5693781	8.91E-06	2.0588	55.8096	0.0369					
1475.74	39.78	0.7146341	4.57E-04	2.8385	42.8505	0.0662	0.604755	2.04E-04	10.1738	69.6288	0.1461	VGPpGPPGPPGPPS	Collagen alpha-1(I) chain	1177	1193	CO1A1_HUMAN	gi124056487
1479.68	39.38	0.6404181	2.20E-02	21.1236	46.999	0.4494	0.5673796	1.71E-04	2.784	16.3416	0.1704	GpPGPpGpPGPPGPPSA	Collagen alpha-1(I) chain	1178	1194	CO1A1_HUMAN	gi124056487
1486.68	21.15	0.7763066	3.74E-04	123.5781	387.5934	0.3188	0.5654329	1.42E-01	203.2056	417.9824	0.4862						
1488.59	20.1	0.6466899	7.30E-03	3.5588	65.1338	0.0546	0.5748287	2.00E-02	70.168	63.074	1.1125						
1492.67	28.98	0.8400697	4.09E-06	29.558	238.168	0.1241	0.6066757	4.56E-04	36.1098	191.5683	0.1885	GSpGSPGPDGKTGPpGP	Collagen alpha-1(I) chain	542	558	CO1A1_HUMAN	gi124056487
1494.64	22.1	0.6174216	3.91E-02	29.28	6.8067	4.3016	0.5999014	2.31E-03	75.9561	32.9968	2.3019						
1495.68	39.41	0.6675958	2.45E-02	45.6358	89.1618	0.5118	0.559593	1.24E-01	68.7888	75.4923	0.9112						
1498.41	35.08	0.6773519	3.71E-03	57.4492	5.2812	10.8781	0.5141196	6.17E-01	22.3861	35.9832	0.6221						
1504.68	20.24	0.6675958	2.25E-03	2.676	36.1305	0.0741	0.5216985	4.94E-01	19.8558	46.1618	0.4301						
1507.74	40.02	0.7909408	2.51E-04	1745.5066	3633.7946	0.4804	0.7645349	2.10E-11	2245.6028	4862.8808	0.4618	VGPpGPpGPpGPPGPPS	Collagen alpha-1(I) chain	1177	1193	CO1A1_HUMAN	gi124056487
1509.67	30.04	0.6250871	2.69E-02	144.3872	12.9402	11.1580	0.5508721	7.88E-02	84.896	30.0114	2.8288	TGPGGDKGDTGPpGPQG	Collagen alpha-1(III) chain	623	639	CO3A1_HUMAN	gi124056490
1511.67	36.27	0.6578397	1.09E-02	8.1705	71.595	0.1141	0.5960341	3.73E-05	5.5307	38.8447	0.1424						
1512.69	26.62	0.7790941	5.10E-05	11.2416	104.214	0.1079	0.556686	8.11E-02	21.042	36.582	0.5752						
1518.68	22.52	0.645993	3.58E-03	1.4812	138.0771	0.0107	0.5371159	2.18E-01	38.0496	160.5101	0.2371						
1519.71	32.56	0.6550523	4.55E-03	17.9225	71.558	0.2505	0.5073194	7.75E-01	8.76	44.7129	0.1959						
1523.84	29.75	0.6752613	2.31E-02	1126.3452	4283.9797	0.2629	0.6546927	1.62E-05	273.0717	1493.76	0.1828	VIDQSRVLNLGPIT	Uromodulin	592	605	UROM_HUMAN	gi137116
1525.48	37.17	0.6961672	1.26E-02	3071.781	1622.07	1.8937	0.5077865	8.86E-01	3795.59	2896.9677	1.3102						
1536.72	19.88	0.6452962	9.48E-03	12.0841	45.3731	0.2663	0.5386991	2.92E-01	34.947	50.5764	0.6910						
1538.69	29.77	0.7254355	3.56E-03	114.7449	242.3766	0.4734	0.5172861	7.26E-01	353.562	257.978	1.3705	PpGEAGKpGEQGVpGD	Collagen alpha-1(I) chain	651	666	CO1A1_HUMAN	gi124056487
1539.73	40.31	0.8299652	2.95E-05	965.5114	3188.3093	0.3028	0.6715116	2.60E-05	1725.4915	3314.0178	0.5207	GpEGPpGEPGpPGPPGP	Collagen alpha-2(V) chain	1209	1225	CO5A2_HUMAN	gi143811378
1552.5	37.22	0.6815331	2.17E-02	3432.8883	1577.66	2.1759	0.5604495	1.86E-01	4835.6343	2962.2362	1.6324						
1552.56	39.51	0.6202091	2.49E-02	34.8831	60.1338	0.5801	0.5031665	9.39E-01	179.5108	129.0708	1.3908						
1560.7	29.78	0.6592334	7.77E-03	37.791	4.2075	8.9818	0.6067276	5.04E-03	98.4704	101.6036	0.9692						
1563.7	29.46	0.656446	4.86E-02	197.3516	312.5116	0.6315	0.6064421	1.13E-02	379.9436	661.2228	0.5746	SpGSPGPDGKTGPpGPAG	Collagen alpha-1(I) chain	543	560	CO1A1_HUMAN	gi124056487
1565.69	26.3	0.6794425	1.81E-02	62.215	124.5766	0.4994	0.5760226	8.29E-02	177.1575	139.209	1.2726						
1573.72	29.86	0.6425087	1.10E-02	42.1239	42.9496	0.9808	0.551495	1.05E-01	24.3378	40.6588	0.5986	GPpGPpGPPGpKGDQGD	Collagen alpha-1(XVII) chain	906	922	COHA1_HUMAN	gi:146345399
1575.75	30.2	0.6278746	3.86E-02	81.8366	7.819	10.4664	0.5708835	2.15E-02	21.8075	86.4066	0.2524	IFPPSDEQLKSGTAS	Ig kappa chain C region	9	23	IGKC_HUMAN	gi125145
1576.6	26.37	0.7240418	4.38E-03	1071.826	573.5051	1.8689	0.5408794	3.97E-01	1161.4225	1343.9443	0.8642						
1578.75	40.23	0.6912892	5.39E-03	188.9516	331.1091	0.5707	0.5882475	6.39E-06	12.1076	110.6924	0.1094	VGPpGPpGPpGPPGPPSA	Collagen alpha-1(I) chain	1177	1194	CO1A1_HUMAN	gi124056487
1579.68	23	0.7369338	2.46E-03	771.8656	293.013	2.6342	0.634837	1.15E-03	1074.4674	430.7244	2.4946						
1585.66	39.84	0.6439024	2.11E-02	27.369	103.9869	0.2632	0.5196221	6.28E-01	78.147	83.3929	0.9371						
1591.71	38.28	0.7940767	6.78E-06	3.137	52.2648	0.0600	0.6116591	1.04E-03	26.2002	58.056	0.4513						
1593.75	39.57	0.7135889	2.40E-04	13.6402	213.67	0.0638	0.5673277	2.87E-02	24.14	84.81	0.2846						
1609.75	30.2	0.6885017	1.56E-02	419.4716	914.6753	0.4586	0.6031458	1.10E-02	525.694	933.6294	0.5631	TGSpGSpGPDGKTGPpGP	Collagen alpha-1(I) chain	541	558	CO1A1_HUMAN	gi124056487
1610.76	40.15	0.6278746	3.07E-02	13.0896	69.4638	0.1884	0.593776	1.07E-03	13.8795	76.854	0.1806	DGPTGpPGPpGpPGPPGL	Collagen alpha-2(I) chain	56	73	CO1A2_HUMAN	gi124056488
1610.87	30.29	0.6986063	1.09E-03	1121.56	72.603	15.4478	0.5496782	6.51E-02	230.5659	384.7872	0.5992	SVIDQSRVLNLGPIT	Uromodulin	591	605	UROM_HUMAN	gi137116
1611.84	23.13	0.6121951	4.89E-02	497.588	309.927	1.6055	0.5002596	9.93E-01	279.9882	694.7616	0.4030	GKVNVDEVGGEALGRL	Hemoglobin subunit beta	17	32	HBB_HUMAN	gi56749856
1620.67	29.49	0.6815331	2.17E-02	284.544	445.5788	0.6386	0.6339026	1.24E-03	268.2682	505.7976	0.5304	GSpGSPGPDGKTGPpGPAG	Collagen alpha-1(I) chain	542	560	CO1A1_HUMAN	gi124056487
1622.72	26.79	0.6735192	2.45E-02	85.0836	153.208	0.5553	0.6813746	6.34E-06	255.3517	126.504	2.0185	DNGASTDDSAAEKKGGT	Plexin domain-containing protein 2	435	451	PXDC2_HUMAN	gi:74749416
1626.69	23.06	0.6480836	1.15E-02	92.9514	9.5427	9.7406	0.6191601	7.18E-05	86.2054	10.1248	8.5143						
1626.7	39.12	0.6675958	2.25E-03	1.6805	52.9692	0.0317	0.6168501	3.73E-09	1.3176	46.1425	0.0286						
1636.74	30.25	0.7707317	4.57E-04	1184.914	2436.896	0.4862	0.6278032	1.08E-03	1215.3683	1803.6396	0.6738	GSpGSpGPDGKTGPpGPAG	Collagen alpha-1(I) chain	542	560	CO1A1_HUMAN	gi124056487
1651.74	30.2	0.6529617	5.19E-03	50.8912	5.6607	8.9903	0.5520141	8.86E-02	20.2048	13.8754	1.4562	pGLQGppGpPGIPGmkG	Collagen alpha-5(IV) chain	140	156	CO4A5_HUMAN	gi461675
1653.88	30.38	0.6686411	3.23E-02	1709.8747	1155.9018	1.4793	0.5018947	9.73E-01	2093.756	2143.114	0.9770	SGSVIDQSRVLNLGPI	Uromodulin	589	604	UROM_HUMAN	gi137116
1658.59	21.46	0.6397213	4.74E-02	88.3305	197.2728	0.4478	0.6026786	3.78E-03	244.4715	195.7473	1.2489						
1658.69	31.69	0.6271777	2.44E-02	480.9024	15.8247	30.3894	0.508669	6.47E-01	1.522	25.8822	0.0588						
1666.78	30.66	0.7111498	6.07E-03	126.5425	281.7962	0.4491	0.6143065	5.66E-03	193.2541	284.2499	0.6799	KpGEQGVpGDLGApGPSG	Collagen alpha-1(I) chain	657	674	CO1A1_HUMAN	gi124056487
1667.9	30.47	0.6376307	1.18E-02	667.3248	13.3734	49.8994	0.5458887	5.67E-02	78.0489	181.9597	0.4289	GSVIDQSRVLNLGPIT	Uromodulin	590	605	UROM_HUMAN	gi137116
1669.69	21.47	0.6533101	4.74E-02	558.5454	111.1443	5.0254	0.6451672	3.44E-04	296.8425	189.6	1.5656	DEAGSEADHEGTHSTK	Fibrinogen alpha chain	605	620	FIBA_HUMAN	gi1706799
1674.73	30.15	0.6463415	8.98E-03	105.1926	11.9214	8.8238	0.639483	1.21E-04	321.7949	140.9408	2.2832						
1678.71	23.82	0.6536585	8.72E-03	84.1898	4.5468	18.5163	0.6150332	8.17E-04	149.1804	24.6312	6.0566						
1684.78	40.3	0.6641115	1.25E-02	29.971	73.083	0.4101	0.6138912	1.71E-08	3.3612	54.447	0.0617						
1686.74	39.15	0.6585366	3.86E-03	15.812	61.124	0.2587	0.6235984	7.57E-10	1.6044	99.657	0.0161						
1689.8	27.55	0.6658537	8.46E-03	14.2065	57.9692	0.2451	0.5614618	8.92E-02	37.4444	76.16	0.4917						
1693.76	20.51	0.6759582	1.64E-02	73.7856	107.7435	0.6848	0.5334821	4.84E-01	147.065	156.2964	0.9409						
1697.74	30.88	0.7365854	2.22E-03	1445.402	897.03	1.6113	0.5270972	5.91E-01	1552.2054	1319.4816	1.1764	NGApGNDGAKGDAGApGApG	Collagen alpha-1(I) chain	700	719	CO1A1_HUMAN	gi124056487
1713.78	39.61	0.7205575	1.42E-04	0.5648	112.7	0.0050	0.6418189	1.67E-13	0.496	75.2232	0.0066						
1716.66	20.18	0.6731707	2.67E-02	458.496	242.649	1.8895	0.6093231	9.02E-03	384.4374	364.959	1.0534						
1716.77	28	0.712892	5.97E-03	278.0113	481.844	0.5770	0.6026267	1.58E-02	404.0823	651.3805	0.6203						
1727.8	39.56	0.7470383	1.98E-04	14.083	83.352	0.1690	0.6607662	1.75E-14	5.1322	96.5248	0.0532						
1739.78	37.96	0.6348432	3.75E-02	23.6759	53.774	0.4403	0.5392182	1.53E-01	15.186	51.9984	0.2920						
1743.8	39.83	0.7456446	5.10E-05	3.6118	149.2821	0.0242	0.6887978	0.00E+00	0.3052	205.1506	0.0015						
1746.81	22.42	0.6571429	1.09E-03	0	18.3179	0.0000	0.5355586	1.87E-01	10.698	4.02	2.6612						
1754.9	31.26	0.6923345	1.44E-02	20176.8522	4025.632	5.0121	0.5007527	9.90E-01	6505.4985	6181.0866	1.0525						
1762.57	38.34	0.6219512	3.13E-02	5.796	20.9168	0.2771	0.511005	7.76E-01	83.1703	67.7236	1.2281						
1764.79	29.83	0.6557491	1.97E-02	44.4466	92.512	0.4804	0.6077658	4.26E-03	180.1416	68.768	2.6196						
1764.81	26.56	0.6533101	4.39E-02	364.5558	154.9786	2.3523	0.8252699	6.02E-17	748.1385	166.668	4.4888						
1769.81	39.5	0.7930314	2.08E-05	12.7308	324.9642	0.0392	0.7108077	4.80E-14	12.868	398.9188	0.0323						
1782.84	25.91	0.702439	9.30E-03	375.808	193.4196	1.9430	0.8265158	5.66E-17	1059.5213	293.4085	3.6111						
1784.81	39.92	0.7362369	4.20E-04	28.1445	295.3512	0.0953	0.7036441	4.05E-12	9.3086	446.9615	0.0208						
1788.84	29.87	0.6543554	2.87E-02	167.2111	411.2802	0.4066	0.5338455	4.17E-01	314.3751	334.828	0.9389						
1791.8	30.81	0.6292683	2.52E-02	25.186	33.2704	0.7570	0.50693	8.81E-01	78.2223	52.576	1.4878						

1793.75	20.72	0.7313589	1.65E-04	6.729	61.6998	0.1091	0.6484635	3.33E-05	63.9676	32.0733	1.9944						
1795.79	25	0.6749129	1.00E-02	22.6944	104.0196	0.2182	0.545162	1.77E-01	51.5655	89.52	0.5760						
1798.72	36.95	0.6891986	7.34E-03	41.1102	172.278	0.2386	0.6405471	1.74E-05	61.2854	160.4826	0.3819						
1798.82	40.04	0.643554	1.44E-02	13.532	145.54	0.0930	0.5675353	1.64E-04	1.7709	58.8756	0.0301	VGPpGPpGPpGPpGPPSAGF	Collagen alpha-1(I) chain	1177	1196	CO1A1_HUMAN	gi124056487
1808.04	21.23	0.7045296	2.44E-03	53.016	930.3606	0.0570	0.5262147	5.04E-01	106.1844	241.437	0.4398	VIDQSRVLNLGPITRK	Uromodulin	592	607	UROM_HUMAN	gi137116
1808.79	23.72	0.6982578	7.95E-03	180.5166	49.16	3.6720	0.6262978	1.41E-03	254.748	162.932	1.5635	FAEEKAVADTRDQADGS	Polymeric immunoglobulin receptor	605	621	PIGR_HUMAN	gi150421625
1812.83	39.98	0.7080139	8.32E-04	10.72	160.9895	0.0666	0.5435008	1.12E-01	8.6294	191.6256	0.0450						
1813.71	31.69	0.8320557	3.00E-05	1651.527	579.5858	2.8495	0.6289452	2.01E-03	2319.0465	1716.7406	1.3508						
1814.72	37.17	0.6292683	4.00E-02	22.6134	99.056	0.2283	0.6150332	2.82E-07	10.2285	46.7306	0.2189						
1817.69	20.23	0.6634146	4.06E-02	3611.85	2835.7269	1.2737	0.5913621	3.55E-02	4051.1592	3981.504	1.0175						
1818.83	30.95	0.6839721	1.89E-02	1256.2797	348.3258	3.6066	0.5933088	2.81E-02	1682.0344	246.196	6.8321	GLpGTGGPpGENGKPGEPGp	Collagen alpha-1(III) chain	642	661	CO3A1_HUMAN	gi124056490
1819.77	19.72	0.6222997	2.23E-02	8.6457	32.3082	0.2676	0.508098	7.56E-01	14.752	27.984	0.5272						
1825.92	19.66	0.6296167	2.85E-02	736.2938	28.6209	25.7257	0.5152876	7.65E-02	0	71.865	0.0000	FRYNSKDRKSQPMGL	Zinc-alpha-2-glycoprotein	59	73	ZA2G_HUMAN	gi292495049
1826.85	39.94	0.7790941	5.10E-05	30.4956	317.097	0.0962	0.6983752	2.70E-13	8.8623	284.6526	0.0311						
1832.84	31.94	0.7818815	5.10E-05	19.0884	108.744	0.1755	0.6690199	2.46E-09	10.4796	42.2172	0.2482	IGPpGPAGApGDKGESGPSGP	Collagen alpha-1(I) chain	769	789	CO1A1_HUMAN	gi124056487
1834.82	31.09	0.6780488	2.45E-02	3513.2821	1118.0266	3.1424	0.6057153	1.34E-02	4485.6812	1231.5672	3.6423	GLpGTGGPpGENGKpGEPGp	Collagen alpha-1(III) chain	642	661	CO3A1_HUMAN	gi124056490
1840.84	41.18	0.8097561	3.20E-05	30.7044	280.3262	0.1095	0.6451931	3.69E-06	57.4693	181.236	0.3171						
1841.84	40.07	0.656446	1.77E-02	52.6711	105.5649	0.4989	0.5860413	7.07E-05	6.3444	69.741	0.0910						
1841.85	24.23	0.6759582	9.75E-04	0.7456	41.218	0.0181	0.5598266	1.17E-02	4.3904	61.9866	0.0708	VGEPGpAGSKGESGNKGEpG	Collagen alpha-2(I) chain	345	364	CO1A2_HUMAN	gi124056488
1844.48	34.26	0.6710801	7.42E-03	109.956	12.3872	8.8766	0.5631748	1.33E-01	152.3909	205.175	0.7427						
1858.84	24.26	0.7066202	4.48E-03	135.392	546.4998	0.2477	0.5099668	8.22E-01	174.6844	503.762	0.3468						
1874.83	30.82	0.6557491	2.73E-02	39.2823	92.5224	0.4246	0.5684956	7.53E-02	97.7691	124.4268	0.7858						
1878.59	30.78	0.6888502	1.10E-02	275.7434	493.025	0.5593	0.7856883	0.00E+00	36.6095	1033.1266	0.0354						
1878.85	32.18	0.6271777	4.69E-02	11.9238	40.4931	0.2945	0.50924	8.17E-01	32.604	25.8192	1.2628						
1888.85	33.13	0.6557491	4.40E-03	10.1205	43.7451	0.2314	0.5252803	5.10E-01	42.85	30.5474	1.4027						
1894.89	32.1	0.7010453	3.74E-04	1.5862	59.1422	0.0268	0.5527928	5.40E-02	11.0006	27.6696	0.3976						
1897.87	40.21	0.6759582	4.53E-03	19.0272	149.0078	0.1277	0.6623754	2.05E-11	8.1552	106.6342	0.0765						
1900.87	32	0.6550523	4.08E-02	112.2069	135.6029	0.8275	0.5423588	3.24E-01	85.323	100.4134	0.8497	ETGPAGRpGEVGPpGPpGPAG	Collagen alpha-1(I) chain	912	932	CO1A1_HUMAN	gi124056487
1915	33.63	0.6313589	4.28E-02	22.1374	44.3992	0.4986	0.5249689	4.49E-01	14.49	21.1106	0.6864						
1936.73	20.24	0.6954704	2.29E-03	426.9076	7.7759	54.9014	0.5554142	2.08E-02	69.387	17.196	4.0351						
1936.87	34.75	0.7860627	1.11E-04	22.5794	100.9194	0.2237	0.5494705	2.75E-01	74.5107	58.8756	1.2656						
1936.88	32.24	0.7484321	1.58E-03	249.6472	499.41	0.4999	0.5791632	7.18E-02	342.3086	384.831	0.8895	GEKGPSGEAGTAGPpGTpGPQG	Collagen alpha-2(I) chain	844	865	CO1A2_HUMAN	gi124056488
1942.67	36.39	0.6334495	9.60E-03	3.111	20.2306	0.1538	0.5752699	3.06E-04	3.3775	25.0866	0.1346						
1942.84	30.96	0.6592334	4.01E-02	70.8998	125.336	0.5657	0.5067483	8.97E-01	90.077	103.014	0.8744						
1946.87	31.69	0.6376307	4.38E-02	179.585	84.804	2.1176	0.579864	4.02E-02	202.5334	148.011	1.3684	SVTEQDSKDSTYSLSSTL	Ig kappa chain C region	54	71	IGKC_HUMAN	gi125145
1947.88	31.61	0.7195122	4.84E-03	179.9154	410.6242	0.4382	0.6189784	3.92E-03	303.3387	436.4013	0.6951						
1948.91	25.03	0.7094077	1.09E-03	494.284	207.7758	2.3789	0.563097	1.66E-03	139.085	64.4924	2.1566	EEGKRGPNGEAGSAGPpGPpG	Collagen alpha-2(I) chain	377	397	CO1A2_HUMAN	gi124056488
1949.81	19.74	0.6216028	2.32E-02	7.2583	25.9687	0.2795	0.5064109	8.24E-01	18.7056	25.3376	0.7383						
1951.86	32.1	0.68223	4.95E-03	10.9803	56.791	0.1933	0.5337417	3.95E-01	48.861	61.1092	0.7996	VNGApGEAGRDGNpGNDGpPG	Collagen alpha-2(I) chain	917	937	CO1A2_HUMAN	gi124056488
1954.01	32.41	0.6494774	9.13E-03	13.267	128.131	0.1035	0.564836	5.12E-02	61.0126	90.3582	0.6752						
1954.91	40.36	0.6494774	2.00E-02	24.6058	110.5702	0.2225	0.6554713	4.01E-11	3.0985	88.8998	0.0349						
1956.06	32.99	0.6578397	4.01E-03	8.508	51.4078	0.1655	0.5534936	7.81E-04	1.7928	60.9331	0.0294						
1964.99	24.79	0.6202091	3.38E-02	630.5728	73.5354	8.5751	0.5461742	2.00E-01	217.4278	276.0437	0.7877						
1967.91	25.78	0.6578397	5.93E-03	53.0839	3.1614	16.7913	0.5044643	8.84E-01	11.5416	17.2422	0.6694						
1969.84	25.23	0.661324	3.23E-02	210.0987	81.2227	2.5867	0.5015314	9.75E-01	289.953	448.06	0.6471						
1969.89	32.29	0.6477352	5.49E-03	2.5475	26.6662	0.0955	0.5346501	3.62E-01	26.964	24.8024	1.0872	FAGEKGPSGEAGTAGPpGTpGP	Collagen alpha-2(I) chain	842	863	CO1A2_HUMAN	gi124056488
1974.95	21.39	0.7644599	1.54E-04	16.6855	128.3964	0.1300	0.5031406	9.39E-01	70.0784	62.7825	1.1162	IkGHRGFPGNPGApGSpGPAG	Collagen alpha-1(III) chain	1105	1125	CO3A1_HUMAN	gi124056490
1983.02	39.84	0.6571429	1.09E-03	0	170.6922	0.0000	0.6130347	2.24E-07	28.2195	742.2597	0.0380						
1990.95	21.42	0.6989547	2.48E-03	14.3685	77.1228	0.1863	0.5006229	9.90E-01	50.065	46.9248	1.0669	IkGHRGFpGNPGApGSpGPAG	Collagen alpha-1(III) chain	1105	1125	CO3A1_HUMAN	gi124056490
1997.89	31.76	0.6825784	2.77E-03	5.866	25.024	0.2344	0.5374273	3.00E-01	15.2856	18.35	0.8330	GApGVkGEpGApGENGTPGQTG	Collagen alpha-2(I) chain	193	214	CO1A2_HUMAN	gi124056488
2003.94	24.62	0.7160279	4.36E-03	67.5168	137.56	0.4908	0.5866383	2.70E-02	71.692	92.846	0.7722	GLpGTGGPpGENGKPGEpGPKG	Collagen alpha-1(III) chain	642	663	CO3A1_HUMAN	gi124056490
2007.92	32.57	0.8076655	2.66E-06	1.1066	77.5719	0.0143	0.5622664	4.98E-02	13.4368	25.272	0.5317						
2008.9	32.29	0.6452962	4.90E-02	353.7088	505.0188	0.7004	0.6526163	1.90E-07	69.183	422.7538	0.1636						
2013.89	31.76	0.7602787	5.24E-04	37.792	119.08	0.3174	0.5701827	1.02E-01	68.8344	92.8929	0.7410	AGpPGPPGppGTSGHpGSpGSpG	Collagen alpha-1(III) chain	176	198	CO3A1_HUMAN	gi124056490
2043.08	27.33	0.6585366	3.86E-03	6.52	42.2429	0.1543	0.5070338	8.08E-01	4.8642	7.919	0.6142						
2046.94	26.47	0.661324	8.04E-03	11.5884	39.6503	0.2923	0.5318729	3.22E-01	16.5459	16.038	1.0317	DAGAPGApGGKGDAGAPGERGpPG	Collagen alpha-1(III) chain	664	687	CO3A1_HUMAN	gi124056490
2058.94	23.15	0.7470383	1.23E-03	122.13	379.8744	0.3215	0.5960341	1.98E-02	185.2138	401.5377	0.4613						
2063.92	19.92	0.6264808	1.44E-02	13.4715	34.3945	0.3917	0.6184593	2.25E-04	74.316	22.1985	3.3478						
2064.92	24.46	0.6937282	1.25E-02	197.8928	313.6334	0.6310	0.5395556	4.10E-01	257.7375	303.2784	0.8498						
2071.97	32.75	0.6898955	5.39E-04	0.583	33.236	0.0175	0.5424626	8.86E-02	7.559	17.847	0.4235						
2073.09	27.44	0.71777	2.45E-03	27.7506	104.8608	0.2646	0.5852367	1.87E-03	10.5586	34.1184	0.3095						
2101.96	27.65	0.6919861	8.50E-04	3.353	33.6088	0.0998	0.5409313	3.43E-02	2.5015	10.7668	0.2323	DGQPGAKGEPGDAGAKGDAGPPGP	Collagen alpha-1(I) chain	820	843	CO1A1_HUMAN	gi124056487
2109.92	24.07	0.8606272	6.62E-07	10.9368	209.3168	0.0522	0.7155575	1.01E-11	15.8821	180.8514	0.0878						
2109.96	21.09	0.6808362	1.10E-02	39.2526	74.2266	0.5288	0.5542722	1.51E-01	46.6308	50.2575	0.9278						
2116.96	33.26	0.6648084	4.95E-03	22.2831	57.6974	0.3862	0.531821	3.70E-01	45.5225	34.0404	1.3373						
2117.93	32.97	0.7226481	4.02E-03	88.2876	211.0416	0.4183	0.583316	4.47E-02	87.9954	108.8314	0.8085	SNGNpGpPGPSGSPGKDGPpGpAG	Collagen alpha-1(III) chain	886	909	CO3A1_HUMAN	gi124056490
2132.91	25.83	0.6602787	3.64E-02	359.8749	111.5319	3.2267	0.6013549	1.44E-02	261.1928	349.968	0.7463						
2133.95	33.1	0.6355401	8.61E-03	3.1305	46.3915	0.0675	0.6066757	1.40E-03	45.8815	16.2282	2.8273						
2148.01	25.29	0.6965157	5.39E-03	72.3	102.0222	0.7087	0.6127492	3.72E-03	86.6603	73.3824	1.1809	QGLpGTGGPpGENGKpGEPGpKG	Collagen alpha-1(III) chain	641	663	CO3A1_HUMAN	gi124056490
2149.97	32.42	0.6428571	1.50E-02	10.467	27.092	0.3864	0.5487697	2.12E-01	24.7836	27.2844	0.9083						
2169.98	31.59	0.6209059	4.20E-02	51.804	121.7448	0.4255	0.6322934	5.32E-08	61.2934	114.6215	0.5347						
2174.99	38.74	0.6522648	6.56E-03	7.7126	178.8025	0.0431	0.5685216	8.70E-05	4.4901	99.526	0.0451						
2187.93	42.5	0.610453	4.68E-02	2.649	14.5638	0.1819	0.5064888	8.14E-01	23.736	20.174	1.1766						

2189	26.89	0.671777	3.03E-02	926.736	1334.8752	0.6942	0.8292411	0.00E+00	367.5561	1107.649	0.3318	ADGQPGAKGEPGDAGAKGDAGPpGP	Collagen alpha-1(I) chain	819	843	CO1A1_HUMAN	gi124056487
2192.02	20.5	0.6466899	1.43E-02	19.8852	79.372	0.2505	0.5542463	9.10E-02	44.445	41.0229	1.0834						
2203.64	35.26	0.7076655	4.32E-04	2.992	54.073	0.0553	0.5846657	1.18E-04	2.2056	39.0156	0.0565						
2210.87	37.9	0.6752613	1.08E-02	113.4384	272.4246	0.4164	0.6311254	7.05E-05	78.0022	262.6913	0.2969						
2210.92	40.43	0.6317073	1.93E-02	12.195	277.5896	0.0439	0.5782807	2.67E-04	13.1436	111.8943	0.1175						
2211	26.09	0.6857143	3.60E-04	0	76.9008	0.0000	0.5091362	6.47E-01	7.2535	29.1819	0.2486	GNSGEPGApGSKGDTGAKGEpGPVG	Collagen alpha-1(I) chain	431	455	CO1A1_HUMAN	gi124056487
2211.98	33.23	0.6271777	3.98E-02	180.8116	27.9426	6.4708	0.5171823	6.99E-01	322.0829	285.7296	1.1272						
2212.15	24.78	0.7142857	1.08E-04	0	56.3085	0.0000	0.6123858	1.97E-10	0.8918	84.4825	0.0106						
2219.01	19.65	0.6229965	2.16E-02	6.5121	54.8545	0.1187	0.5733233	2.30E-02	44.7832	25.8012	1.7357	DEAGSEADHEGTHSTKRGHAK	Fibrinogen alpha chain	605	625	FIBA_HUMAN	gi1706799
2219.04	34.12	0.6344948	2.87E-02	9.3405	50.812	0.1838	0.5808243	1.55E-03	14.76	66.8305	0.2209	GRTGDAGPVGpPGPpGPpGPPGPPS	Collagen alpha-1(I) chain	1169	1193	CO1A1_HUMAN	gi124056487
2219.99	33.13	0.6292683	1.53E-02	4.5808	84.2425	0.0544	0.5274086	3.97E-01	27.7712	29.3208	0.9472						
2231.99	25.62	0.671777	8.02E-03	25.7346	131.8149	0.1952	0.5507164	3.87E-02	12.9636	42.1498	0.3076						
2234.01	33.96	0.6703833	2.45E-03	210.5448	2.3499	89.5973	0.6738995	3.89E-07	173.0655	57.2319	3.0239	YIEFNKEIPAWVPFDPAAQ	Zinc-alpha-2-glycoprotein	144	162	ZA2G_HUMAN	gi292495049
2235.04	34.17	0.7491289	1.52E-03	602.5812	1098.5929	0.5485	0.7660403	2.62E-12	364.7952	1109.3116	0.3288	GRTGDAGPVGPPGPpGppGpPGPPS	Collagen alpha-1(I) chain	1169	1193	CO1A1_HUMAN	gi124056487
2246.02	26.93	0.8324042	4.57E-06	18.372	354.2084	0.0519	0.6484894	1.02E-12	2.2644	123.0816	0.0184	GADGQPGAKGEPGDAGAKGDAGPpGP	Collagen alpha-1(I) chain	818	843	CO1A1_HUMAN	gi124056487
2250.02	34.2	0.6466899	3.46E-02	38.0889	117.2847	0.3248	0.5678727	6.84E-02	68.603	127.4502	0.5383	GEpGDDGPSGAEGpPGpQGLAGQR	Collagen alpha-1(II) chain	957	980	CO2A1_HUMAN	gi124056489
2256.88	36.21	0.7428571	3.00E-05	0	269.4118	0.0000	0.590843	4.20E-05	4.1748	108.3551	0.0385						
2280.94	36.22	0.6425087	4.28E-02	65.6829	215.325	0.3050	0.6938331	1.42E-09	159.2599	325.782	0.4889						
2280.97	26.16	0.6989547	7.23E-04	460.9958	0.8244	559.1895	0.5800457	1.16E-02	76.622	96.264	0.7960						
2281.94	36.35	0.6320557	1.05E-02	3.892	513.7878	0.0076	0.5979028	1.23E-05	13.1358	271.7925	0.0483						
2281.98	33.93	0.7031359	9.62E-03	11828.71	7077.9744	1.6712	0.5841206	5.56E-02	13222.79	10079.1432	1.3119	ANGApGNDGAKGDAGApGApGSQGApG	Collagen alpha-1(I) chain	699	725	CO1A1_HUMAN	gi124056487
2282.02	22.24	0.7275261	2.77E-03	289.525	593.6494	0.4877	0.7587988	5.62E-11	989.712	464.6961	2.1298	ApGHpGPpGPVGPAGKSGDRGESGP	Collagen alpha-1(III) chain	1045	1069	CO3A1_HUMAN	gi124056490
2290.06	33.96	0.7735192	1.53E-04	19.5162	187.6885	0.1040	0.6475291	1.07E-08	13.6456	74.235	0.1838						
2294.02	28.43	0.6808362	7.19E-03	18.508	56.9376	0.3251	0.5005451	9.90E-01	19.6175	16.596	1.1821						
2298	26.39	0.6857143	7.95E-03	23.2152	84.06	0.2762	0.5801235	1.80E-02	36.636	49.875	0.7346						
2308.02	27.34	0.697561	1.09E-02	160.9119	303.8547	0.5296	0.6383669	3.62E-04	107.5848	222.8622	0.4827	ADGQpGAKGEpGDAGAKGDAGppGPA	Collagen alpha-1(I) chain	819	844	CO1A1_HUMAN	gi124056487
2314.01	33.66	0.6634146	4.05E-02	747.2435	1186.7406	0.6297	0.6795058	8.68E-06	609.926	817.6794	0.7459						
2320.02	23.32	0.6867596	4.07E-03	10.212	42.3504	0.2411	0.5011161	9.78E-01	17.2431	25.809	0.6681						
2327.08	35.31	0.7156794	1.07E-03	11.952	78.894	0.1515	0.6132683	2.61E-03	95.175	17.2662	5.5122						
2333.04	27.43	0.7066202	2.74E-04	0.3938	55.3668	0.0071	0.5496262	9.10E-04	0.6054	7.7088	0.0785	GADGQPGAKGEpGDAGAKGDAGPpGPA	Collagen alpha-1(I) chain	818	844	CO1A1_HUMAN	gi124056487
2336.04	26.66	0.6439024	3.91E-02	17.9253	50.0667	0.3580	0.5403343	2.45E-01	32.3488	38.8154	0.8334	ERGSEGSPGHpGQPGpPGPpGApGP	Collagen alpha-1(III) chain	1171	1195	CO3A1_HUMAN	gi124056490
2343.91	21.03	0.645993	9.14E-03	8.05	43.919	0.1833	0.5119394	7.43E-01	39.6066	32.481	1.2194						
2344.07	34.33	0.7512195	4.09E-04	19.1994	92.6706	0.2072	0.6447	1.08E-04	215.345	37.175	5.7927						
2352.05	26.75	0.7139373	5.97E-03	155.2284	258.7087	0.6000	0.6486971	9.51E-06	85.3752	165.08	0.5172						
2363.95	30.88	0.7512195	3.32E-05	0.8382	105.9525	0.0079	0.6400021	1.32E-10	5.8232	38.6331	0.1507						
2368.04	26.75	0.6850174	1.81E-02	94.7835	190.1841	0.4984	0.5570235	1.63E-01	97.896	137.38	0.7126						
2370.12	30.74	0.7574913	3.00E-05	2.091	60.0534	0.0348	0.5566341	8.14E-02	19.7236	38.4522	0.5129						
2372.08	21.83	0.6554007	8.00E-03	8.69	36.532	0.2379	0.6055596	3.24E-07	1.476	30.2064	0.0489						
2406.04	21.67	0.7062718	4.65E-04	2.9505	29.1226	0.1013	0.6381593	3.24E-05	26.6646	12.1065	2.2025						
2412.1	27.08	0.7184669	1.85E-03	23.6544	107.2386	0.2206	0.5484842	2.13E-01	38.2239	49.8094	0.7674	RGGAGPpGPEGGKGAAGPpGppGAAGTPG	Collagen alpha-1(III) chain	695	723	CO3A1_HUMAN	gi124056490
2413.74	35.59	0.6940767	7.60E-04	3.4235	65.5664	0.0522	0.6686047	0.00E+00	0	69.4008	0.0000						
2414.1	28.33	0.6264808	1.44E-02	3.751	14.4863	0.2589	0.5178571	4.98E-01	4.059	5.4252	0.7482	ADGQPGAKGEPGDAGAKGDAGPpGPAGP	Collagen alpha-1(I) chain	819	846	CO1A1_HUMAN	gi124056487
2430.08	25.7	0.7923345	3.76E-05	16.626	98.2853	0.1692	0.6753789	9.13E-12	6.7448	94.4599	0.0714						
2430.59	35.54	0.6418118	2.53E-02	152.4913	42.4564	3.5917	0.5905316	7.74E-03	272.9418	183.5784	1.4868						
2432.17	34.41	0.6222997	2.23E-02	3.1395	22.1743	0.1416	0.5734531	1.89E-02	27.703	13.1994	2.0988						
2446.09	28.37	0.7637631	4.25E-04	106.7067	283.4616	0.3764	0.7381125	1.37E-10	69.3378	208.1649	0.3331	ADGQpGAKGEpGDAGAKGDAGpPGPAGP	Collagen alpha-1(I) chain	819	846	CO1A1_HUMAN	gi124056487
2458.08	34.2	0.7317073	7.88E-04	21.1871	104.0634	0.2036	0.5290179	4.51E-01	32.8524	37.237	0.8823						
2459.13	20.85	0.6508711	7.17E-03	6.0242	52.5437	0.1147	0.5432932	9.84E-02	35.4	27.3828	1.2928	PpGKNGDDGEAGKPGRpGERGppGP	Collagen alpha-1(I) chain	225	249	CO1A1_HUMAN	gi124056487
2472.12	27.99	0.6620209	1.50E-02	19.844	85.4119	0.2323	0.5800457	1.62E-02	23.552	64.4105	0.3657	pPGADGQPGAKGEPGDAGAKGDAGPpGp	Collagen alpha-1(I) chain	816	843	CO1A1_HUMAN	gi124056487
2472.68	35.72	0.7857143	4.06E-06	0	200.2353	0.0000	0.5909468	7.84E-09	0	59.248	0.0000						
2474.09	19.8	0.625784	1.85E-02	4.5339	50.8059	0.0892	0.572311	1.58E-02	62.9664	53.802	1.1703	GRDGNpGNDGPpGRDGQpGHKGER	Collagen alpha-2(I) chain	925	948	CO1A2_HUMAN	gi124056488
2474.07	34.27	0.6724739	4.02E-03	4.717	34.83	0.1354	0.5018169	9.62E-01	14.992	10.6624	1.4061						
2483.12	27.57	0.6954704	1.29E-02	388.808	655.72	0.5929	0.7220203	1.20E-08	244.6308	543.3097	0.4503	AGQDGRpGPpGppGARGQAGVmGFpG	Collagen alpha-1(I) chain	559	584	CO1A1_HUMAN	gi124056487
2487.09	25.91	0.7355401	1.33E-04	4.023	53.8101	0.0748	0.619186	9.98E-09	3.468	95.9424	0.0361						
2487.13	28.27	0.7167247	5.63E-03	202.6672	437.0184	0.4637	0.6079215	9.55E-03	252.0486	334.737	0.7530	GADGQPGAKGEpGDAGAKGDAGPpGPAGP	Collagen alpha-1(I) chain	818	846	CO1A1_HUMAN	gi124056487
2501.12	34.39	0.6480836	2.51E-02	36.01	59.1675	0.6086	0.5997975	1.03E-02	140.2926	399.619	0.3511						
2503.13	28.4	0.6487805	4.08E-02	36.3104	82.908	0.4380	0.5346761	3.92E-01	27.6538	27.54	1.0041	GADGQpGAKGEpGDAGAKGDAGpPGPAGP	Collagen alpha-1(I) chain	818	846	CO1A1_HUMAN	gi124056487
2527.11	25.74	0.6571429	1.09E-03	0	20.9312	0.0000	0.5420473	9.55E-04	0.403	3.567	0.1130	DAPGQYGAYFHDDGFLAFPGHVF	Basement membrane-specific heparan sulfate proteoglycan core protein	4197	4219	PGBM_HUMAN	gi218512120
2529.14	28.25	0.7222997	8.54E-04	13.6035	72.7491	0.1870	0.5374273	3.00E-01	35.076	78.884	0.4447	GPPGADGQpGAKGEpGDAGAKGDAGpPGP	Collagen alpha-1(I) chain	815	843	CO1A1_HUMAN	gi124056487
2544.11	26.06	0.7857143	4.06E-06	0	62.8311	0.0000	0.668994	4.80E-14	1.2333	100.648	0.0123						
2544.13	28.26	0.7010453	7.34E-03	51.7543	166.3388	0.3111	0.7008929	1.19E-09	33.0234	160.2536	0.2061						
2545.12	28.2	0.7142857	1.09E-03	171.8496	11.4191	15.0493	0.6490345	7.80E-05	174.581	101.052	1.7276	GPpGADGQpGAKGEpGDAGAKGDAGpPGP	Collagen alpha-1(I) chain	815	843	CO1A1_HUMAN	gi124056487
2557.17	28.25	0.7052265	7.86E-03	118.0854	231.1336	0.5109	0.6088819	5.16E-03	78.8835	124.6707	0.6327	KNGETGPQGPpGPTGPGGDKGDTGPPGp	Collagen alpha-1(III) chain	610	637	CO3A1_HUMAN	gi124056490
2566.37	19.52	0.6829268	8.50E-04	2089.0792	0	∞	0.5223214	1.02E-01	37.678	0.8452	44.5788						
2576.12	34.26	0.7456446	1.42E-04	4.56	125.415	0.0364	0.6545889	3.37E-05	46.0278	99.968	0.4604						
2578.14	27.35	0.6195122	2.57E-02	7.6279	28.0705	0.2717	0.5555959	1.69E-02	7.7224	23.6523	0.3265	VNGApGEAGRDGNpGNDGppGRDGQPG	Collagen alpha-2(I) chain	917	943	CO1A2_HUMAN	gi124056488
2596.23	34.9	0.6533101	3.43E-02	22.9262	64.1706	0.3573	0.55326	2.00E-01	47.8422	55.4399	0.8630	GLPGpAGppGEAGKPGEQGVPGDLGApGP	Collagen alpha-1(I) chain	644	672	CO1A1_HUMAN	gi124056487
2599.19	28.27	0.6731707	2.18E-02	147.1326	392.6939	0.3747	0.6499169	2.20E-04	196.0335	320.309	0.6120	AGPpGApGApGApGPVGPAGKSGDRGETGP	Collagen alpha-1(I) chain	1042	1071	CO1A1_HUMAN	gi124056487
2601.1	34.38	0.641115	1.87E-02	64.6686	4.7212	13.6975	0.5159365	6.47E-01	20.8215	23.8512	0.8730						
2626.21	27.84	0.6714286	6.10E-04	0	36.5194	0.0000	0.5668086	4.03E-02	29.0332	10.6032	2.7382						
2627.21	28.03	0.6738676	2.48E-03	2.4773	48.22	0.0514	0.5139639	6.47E-01	10.1322	21.5951	0.4692	VRGEpGPpGPAGAAGPAGNpGADGQpGAKG	Collagen alpha-1(I) chain	369	398	CO1A1_HUMAN	gi124056487
2647.2	23.47	0.6554007	4.11E-02	87.6213	155.4	0.5638	0.5063071	9.00E-01	92.155	91.3146	1.0092	NRGERGSEGSPGHPGQPGPpGPpGApGP	Collagen alpha-1(III) chain	1168	1195	CO3A1_HUMAN	gi124056490

2658.27	19.48	0.7219512	2.89E-03	2655.4902	226.0626	11.7467	0.5388289	3.61E-01	1301.5288	1127.6125	1.1542						
2687.22	28.99	0.7512195	1.09E-03	56.8395	203.736	0.2790	0.704293	1.37E-08	35.3906	109.7146	0.3226	KDGEAGAQGPpGPAGPAGERGEQGPAGSpG	Collagen alpha-1(I) chain	612	641	CO1A1_HUMAN	gi124056487
2697.23	29.24	0.8062718	4.32E-06	3.5395	85.0938	0.0416	0.5710133	3.06E-02	16.7508	54.3375	0.3083	PpGADGQPGAKGEpGDAGAKGDAGpPGPAGP	Collagen alpha-1(I) chain	816	846	CO1A1_HUMAN	gi124056487
2715.23	34.78	0.6501742	7.36E-03	4.0663	50.7566	0.0801	0.5077346	8.36E-01	22.8228	25.3745	0.8994						
2723.28	23.87	0.6620209	8.89E-03	12.7428	36.5562	0.3486	0.5255399	4.05E-01	12.6718	12.6825	0.9992	IQGPIGPpGEEGKRGPRGDpGTVGPpGP	Collagen alpha-2(V) chain	490	517	CO5A2_HUMAN	gi143811378
2733.78	34.16	0.6592334	2.25E-02	43.5834	160.1748	0.2721	0.6294643	4.16E-05	33.1854	74.4702	0.4456						
2748.79	36.38	0.6780488	9.48E-03	40.5152	70.1043	0.5779	0.5267857	5.58E-01	55.2015	60.2399	0.9164						
2753.83	36.25	0.7264808	6.86E-04	27.501	164.2911	0.1674	0.6275436	7.22E-06	16.0511	56.6649	0.2833						
2758.24	42.17	0.7944251	5.09E-06	23.943	193.0383	0.1240	0.5988113	8.97E-04	27.4584	69.759	0.3936						
2758.25	28.99	0.6944251	5.87E-03	38.4144	78.2397	0.4910	0.5924003	1.00E-03	15.7989	33.0036	0.4787	KNGETGPQGPpGPTGpGGDKGDTGPpGPQG	Collagen alpha-1(III) chain	610	639	CO3A1_HUMAN	gi124056490
2763.19	34.92	0.6278746	1.34E-02	14.0315	31.0713	0.4516	0.5168708	6.47E-01	18.2322	16.0854	1.1335						
2789.31	41.21	0.671777	3.52E-03	10.3936	40.7081	0.2553	0.5504568	1.03E-01	9.6866	34.7323	0.2789						
2802.82	36.34	0.6191638	4.53E-02	93.8332	14.0679	6.6700	0.5441757	3.11E-01	318.6647	143.9081	2.2144						
2807.29	29.29	0.6571429	8.60E-03	12.709	39.4224	0.3224	0.5037895	9.14E-01	15.3884	24.3522	0.6319						
2828.86	36.5	0.7344948	2.94E-04	9.799	158.2038	0.0619	0.5994601	7.41E-07	5.9344	32.9647	0.1800						
2853.38	36.86	0.6212544	3.22E-02	31.765	91.0214	0.3490	0.5494705	8.28E-02	32.2012	46.225	0.6966						
2887.27	24.07	0.6466899	3.46E-03	1.1946	132.1313	0.0090	0.6023671	1.99E-07	4.2972	34.6712	0.1239						
2888.35	24.1	0.6620209	3.12E-03	6.0345	74.0333	0.0815	0.5454734	5.29E-02	11.964	32.3986	0.3693						
2898.31	29.25	0.6745645	2.32E-02	68.992	129.7604	0.5317	0.5450062	3.00E-01	66.0603	56.8269	1.1625						
2924.35	35.95	0.6222997	3.07E-02	63.504	11.565	5.4911	0.5318729	1.94E-01	10.1647	8.514	1.1939						
2936.54	20.1	0.7414634	1.42E-04	5356.0455	61.6524	86.8749	0.5505347	1.41E-02	235.4825	1.3742	171.3597	DAHKSEVAHRFKDLGEENFKALVLIA	Serum albumin	25	50	ALBU_HUMAN	gi113576
2952.31	25.17	0.7114983	6.67E-04	11.864	321.5233	0.0369	0.5255399	3.38E-01	14.2824	107.3804	0.1330	AVADTRDQADGSRASVDSGSSEEQGGSSRA	Polymeric immunoglobulin receptor	610	639	PIGR_HUMAN	gi150421625
2959.26	35.14	0.6787456	8.60E-03	672.9348	138.52	4.8580	0.5772425	2.99E-02	283.9239	361.2798	0.7859						
2981.3	22.22	0.6585366	2.22E-03	67.7504	0	∞	0.5617213	1.56E-02	50.7518	19.5096	2.6014						
2983.31	22.25	0.712892	1.58E-03	51.6525	187.104	0.2761	0.5772425	4.88E-04	48.6222	61.0759	0.7961	GESGREGAPGAEGSpGRDGSpGAKGDRGETGP	Collagen alpha-1(I) chain	1010	1041	CO1A1_HUMAN	gi124056487
2993.38	29.43	0.6351916	1.62E-02	5.127	91.4838	0.0560	0.5961379	2.71E-03	29.4987	121.017	0.2438						
2994.38	29.6	0.7344948	5.24E-04	25.0733	240.0612	0.1044	0.5189732	6.68E-01	117.5895	98.7939	1.1903						
3001.43	35.4	0.7547038	1.09E-03	14339.115	5021.7285	2.8554	0.5360777	4.58E-01	11178.7925	12532.0536	0.8920						
3023.41	20.95	0.6878049	1.18E-02	127.8091	227.7202	0.5613	0.5157288	7.11E-01	122.1552	113.3685	1.0775						
3038.38	22.32	0.7142857	1.08E-04	0	79.4898	0.0000	0.5193625	2.25E-01	26.7585	46.5878	0.5744	RGERGEAGIpGVpGAKGEDGKDGSpGEpGANG	Collagen alpha-1(III) chain	446	477	CO3A1_HUMAN	gi124056490
3039.39	24.61	0.6341463	4.71E-02	32.637	75.4208	0.4327	0.5770349	2.12E-02	28.4735	36.4371	0.7814						
3047.44	29.37	0.7770035	2.51E-04	87.4665	149.0294	0.5869	0.6172135	4.64E-06	24.0642	72.0533	0.3340						
3055.36	35.11	0.6456446	9.32E-03	272.8432	19.101	14.2842	0.5049834	8.08E-01	13.5396	27.1191	0.4993						
3058.38	24.82	0.7003484	6.09E-03	134.572	368.5242	0.3652	0.5376869	4.25E-01	252.917	247.2318	1.0230	QNGEPGGKGERGApGEKGEGGppGVAGPpGGSGP	Collagen alpha-1(III) chain	817	850	CO3A1_HUMAN	gi124056490
3076.23	19.58	0.6794425	1.97E-02	620.824	225.8172	2.7492	0.633098	9.60E-04	463.6512	329.8095	1.4058						
3080.41	29.98	0.6721254	3.47E-03	156.4758	13.8402	11.3059	0.5190511	6.16E-01	81.57	97.9128	0.8331						
3081.42	29.83	0.7839721	2.51E-04	136.873	383.0699	0.3573	0.6400021	8.85E-05	57.1395	179.7752	0.3178						
3108.38	36.06	0.6592334	2.40E-02	90.3816	83.994	1.0760	0.5818885	3.56E-02	64.8036	36.822	1.7599						
3110.43	29.98	0.6592334	1.14E-02	8.2008	63.554	0.1290	0.5053987	8.88E-01	11.6823	22.2201	0.5258						
3114.43	30.29	0.7094077	5.94E-03	108.4588	239.8781	0.4521	0.6184593	4.08E-04	50.7125	123.8774	0.4094						
3122.36	24.62	0.6724739	1.14E-03	1.9326	35.9307	0.0538	0.5837832	5.05E-05	1.639	20.2654	0.0809						
3132.46	31.18	0.7101045	3.52E-03	174.5149	38.688	4.5108	0.5141715	6.68E-01	63.1047	59.2746	1.0646						
3133.47	31.2	0.6634146	2.46E-02	95.761	191.526	0.5000	0.6143584	1.39E-05	35.769	93.28	0.3835	GADGQPGAKGEPGDAGAKGDAGPPGPAGpAGpPGPIG	Collagen alpha-1(I) chain	818	854	CO1A1_HUMAN	gi124056487
3139.49	29.48	0.7038328	8.72E-03	1950.472	803.5209	2.4274	0.515625	7.59E-01	911.729	1078.8096	0.8451						
3158.44	29.71	0.6689895	3.19E-02	558.6516	249.0254	2.2434	0.6237282	2.54E-03	654.9774	334.0025	1.9610	GERGSpGGpGAAGFpGARGLpGpPGSNGNPGPpGp	Collagen alpha-1(III) chain	861	895	CO3A1_HUMAN	gi124056490
3182.29	22.22	0.6278746	1.34E-02	12.832	81.3161	0.1578	0.5569456	7.97E-02	75.747	115.1566	0.6578						
3183.43	25.03	0.6731707	1.11E-03	3.2932	109.9418	0.0300	0.5333005	1.79E-01	6.3756	35.5355	0.1794						
3200.42	35.77	0.6766551	1.00E-02	757.8141	110.0895	6.8836	0.5610984	8.07E-02	210.3325	305.0146	0.6896						
3205.27	19.66	0.7038328	9.13E-03	988.176	450.424	2.1939	0.6132164	6.97E-03	699.168	582.825	1.1996						
3205.35	23.59	0.6299652	4.85E-02	69.126	15.63	4.4226	0.5626038	1.73E-02	18.495	24.0534	0.7689						
3218.94	35.01	0.6522648	1.09E-02	8.9412	84.14	0.1063	0.5082537	8.37E-01	76.245	46.035	1.6562						
3223.39	24.76	0.7184669	7.94E-04	10.5432	72.8406	0.1447	0.5713767	4.65E-02	35.5572	20.5504	1.7302						
3225.43	22.75	0.6445993	8.11E-03	3.0338	56.882	0.0533	0.51155	7.34E-01	15.3387	17.2578	0.8888	pPGESGREGApGAEGSpGRDGSpGAkGDRGETGP	Collagen alpha-1(I) chain	1008	1041	CO1A1_HUMAN	gi124056487
3238.5	30.73	0.6477352	5.49E-03	3.6745	27.591	0.1332	0.500571	9.85E-01	5.2299	10.591	0.4938						
3248.39	22.63	0.6369338	1.23E-02	347.5648	18.1128	19.1889	0.5415542	1.51E-01	211.149	128.4163	1.6443						
3249.55	20.9	0.6703833	3.06E-03	4.9539	66.628	0.0744	0.5415023	1.52E-01	29.8422	17.2596	1.7290						
3256.53	33.03	0.7181185	5.46E-03	2965.9168	1340.696	2.2122	0.5405939	3.96E-01	801.3675	1354.1056	0.5918						
3265.43	36.09	0.6808362	2.03E-02	232.0032	649.9642	0.3569	0.6577554	5.00E-05	324.3996	554.8419	0.5847						
3287.51	36.44	0.6167247	3.46E-02	5.554	34.8285	0.1595	0.5436306	1.37E-01	33.5711	8.526	3.9375						
3333.72	23.83	0.6445993	2.25E-02	213.5116	48.9818	4.3590	0.5516507	1.03E-01	50.8128	48.327	1.0514						
3350.55	31.02	0.7390244	1.58E-03	70.3516	212.0544	0.3318	0.6704215	2.35E-07	28.8949	96.0526	0.3008						
3363.54	30.22	0.6891986	9.56E-03	187.0565	43.8226	4.2685	0.6222228	1.60E-03	182.4405	86.5232	2.1086						
3365.55	30.99	0.6484321	8.04E-03	90.7358	2.0268	44.7680	0.5797861	5.10E-03	30.8476	11.251	2.7418						
3366.45	25.36	0.6620209	6.70E-03	11.874	39.001	0.3045	0.516767	6.27E-01	13.754	7.248	1.8976						
3366.56	31	0.6648084	1.56E-02	31.108	78.516	0.3962	0.5473681	1.20E-01	14.9362	35.711	0.4183						
3368.49	36.42	0.6271777	1.39E-02	4.8685	16.1758	0.3010	0.5536752	6.26E-02	16.29	4.4856	3.6316						
3386.57	26.05	0.6313589	1.66E-02	9.8959	46.7636	0.2116	0.5288621	3.46E-01	14.4954	21.3304	0.6796						
3391.58	31.96	0.7355401	1.07E-03	44.016	155.181	0.2836	0.6023152	6.03E-04	18.0608	52.6575	0.3430						
3401.6	25.47	0.7191638	5.34E-03	832.5	1378.8672	0.6038	0.6785195	9.09E-06	479.7196	952.2952	0.5038						
3404.62	25.94	0.6592334	2.87E-02	2044.3357	999.9693	2.0444	0.6293345	6.71E-04	930.5172	745.2162	1.2487						
3416.6	36.85	0.7108014	4.38E-03	39.3024	83.1464	0.4727	0.5545058	1.74E-01	44.1595	54.1918	0.8149						
3433.6	36.72	0.6344948	3.46E-02	113.5602	10.8103	10.5048	0.5198297	2.14E-01	0.9447	4.3799	0.2157						

3441.61	35.04	0.6188153	4.61E-02	39.9732	219.7652	0.1819	0.5750363	5.52E-03	57.2604	103.2696	0.5545					
3442.59	32.68	0.6390244	3.67E-02	54.538	276.2718	0.1974	0.6263497	3.98E-07	10.0752	52.3276	0.1925					
3448.59	32.11	0.7142857	2.27E-03	32.5344	161.544	0.2014	0.6111919	1.48E-05	8.591	57.1525	0.1503					
3457.6	35.88	0.6135889	4.61E-02	151.667	418.4244	0.3625	0.6277512	6.01E-06	125.5215	454.8921	0.2759					
3465.63	27.07	0.6606272	1.92E-03	6.712	561.1156	0.0120	0.5383617	6.74E-03	0.6622	207.215	0.0032					
3473.6	33.01	0.6456446	6.11E-03	3.749	25.3266	0.1480	0.5720775	2.41E-02	42.6735	65.1392	0.6551					
3474.65	23.99	0.6571429	1.09E-03	0	102.6596	0.0000	0.5420473	9.55E-04	10.314	45.1232	0.2286					
3501.6	31.93	0.7184669	1.13E-03	21.9165	69.276	0.3164	0.5239826	5.57E-01	121.215	47.7939	2.5362					
3546.67	26.22	0.7679443	1.35E-04	19.8764	293.5218	0.0677	0.5746989	6.42E-02	198.0176	316.0444	0.6265					
3582.7	19.47	0.6536585	4.06E-02	584.9046	252.6138	2.3154	0.6084147	3.91E-03	423.1316	384.0672	1.1017					
3590.7	29	0.6874564	3.95E-03	25.227	176.1693	0.1432	0.635434	8.09E-05	33.0008	114.5864	0.2880					
3609.47	20.2	0.6648084	2.62E-03	3.8085	73.8483	0.0516	0.5484323	5.27E-02	13.636	145.8744	0.0935					
3617.74	27.01	0.6557491	2.15E-02	24.5784	123.6417	0.1988	0.5290438	4.11E-01	23.0208	47.0492	0.4893					
3630.44	21.78	0.7519164	8.53E-04	346.0784	95.1714	3.6364	0.6555233	4.10E-05	218.9096	193.8015	1.1296					
3650.69	32.24	0.6299652	1.18E-02	4.5135	21.6132	0.2088	0.5145349	5.47E-01	3.5216	14.5788	0.2416					
3681.72	32.02	0.6682927	2.73E-02	73.5632	124.7115	0.5899	0.5990968	6.47E-03	36.2736	68.1264	0.5324					
3682.7	27.51	0.7017422	1.34E-03	4.9176	47.7456	0.1030	0.5092919	7.79E-01	11.4928	12.281	0.9358					
3685.83	22.2	0.6682927	3.25E-02	2326.2244	1164.1542	1.9982	0.5361815	4.46E-01	1451.7048	1372.168	1.0580	GRPEAQPPPLSSEHKEPVAGDAVPGPKDGSAPEVRGA	Neurosecretory protein VGF	26	62	VGF_HUMAN	gi20140360
3696.76	26.94	0.6933798	1.25E-02	197.8928	328.2235	0.6029	0.6711742	1.35E-07	44.066	153.8502	0.2864	ARGNDGATGAAGPpGPTGPAGppGFpGAVGAKGEAGPQGPRG	Collagen alpha-1(I) chain	321	362	CO1A1_HUMAN	gi124056487
3705.9	22.1	0.6714286	6.10E-04	0	210.9802	0.0000	0.513289	6.56E-01	31.5735	108.2614	0.2916					
3734.72	32.5	0.7581882	1.07E-03	465.1455	995.3944	0.4673	0.7854288	1.57E-13	223.5805	714.429	0.3129					
3735.71	27.17	0.6714286	6.10E-04	0	125.5722	0.0000	0.50462	8.88E-01	9.4766	17.4752	0.5423					
3737.66	37.21	0.6794425	1.97E-02	109.6144	246.9522	0.4439	0.5563227	1.84E-01	160.3606	131.238	1.2219					
3738.22	24.75	0.6264808	2.88E-02	126.378	30.5784	4.1329	0.5752959	6.24E-03	83.7732	37.2564	2.2486					
3774.79	33.1	0.6515679	1.81E-02	15.3425	76.0334	0.2018	0.5791632	8.82E-03	12.75	48.272	0.2641					
3775.72	31.36	0.6550523	5.68E-03	5.3865	73.5449	0.0732	0.5319248	1.17E-01	1.0308	16.6595	0.0619					
3775.75	25.58	0.7560976	1.09E-03	2671.0995	595.4907	4.4855	0.6317224	1.38E-03	476.8289	1104.9499	0.4315					
3788.82	25.19	0.6439024	4.08E-02	92.043	185.2956	0.4967	0.6115033	3.64E-03	44.4921	209.8271	0.2120					
3802.71	32.44	0.6599303	1.38E-02	120.9294	24.906	4.8554	0.5314057	3.39E-01	54.9045	68.1822	0.8053	ANGApGNDGAKGDAGApGApGSQGApGLQGMpGERGAAGLPGp	Collagen alpha-1(I) chain	699	741	CO1A1_HUMAN	gi124056487
3831.81	28.48	0.7080139	6.39E-03	334.662	1519.3236	0.2203	0.6072986	7.74E-03	406.625	827.1585	0.4916					
3839.81	19.7	0.6958188	1.25E-02	2145.5016	884.9512	2.4244	0.5602679	1.84E-01	1411.6333	1417.5792	0.9958					
3872.57	20.39	0.6174216	2.87E-02	8.6926	26.2725	0.3309	0.5363891	1.19E-01	11.4984	11.924	0.9643					
3890.78	24.39	0.6864111	1.13E-03	198.4827	0.6429	308.7303	0.6668916	2.08E-07	218.2606	40.7384	5.3576					
3891.75	24.53	0.7149826	4.48E-03	351.6406	579.9486	0.6063	0.6043137	1.06E-02	192.7638	353.0619	0.5460					
3913.69	30.83	0.6592334	2.15E-03	3.824	71.638	0.0534	0.6129309	3.77E-09	1.222	42.0274	0.0291					
3927.82	33.6	0.6707317	2.93E-02	138.8268	253.688	0.5472	0.659157	1.65E-05	111.7044	200.7905	0.5563					
3975.7	19.93	0.6836237	8.27E-04	6.961	97.424	0.0715	0.5236711	2.94E-01	23.9253	146.1404	0.1637					
3984.65	21.26	0.6940767	3.63E-03	73.7868	348.4512	0.2118	0.5905835	1.85E-02	198.6754	346.2497	0.5738					
3986.65	20.6	0.8013937	1.42E-04	6212.2115	2375.9032	2.6147	0.554973	2.30E-01	2915.4309	3819.8356	0.7632					
3996.66	20.92	0.7135889	4.55E-03	863.9928	207.9568	4.1547	0.5432932	3.11E-01	311.9061	559.0012	0.5580					
4059.65	20.45	0.6766551	7.19E-03	108.8442	307.4382	0.3540	0.6004464	4.75E-03	179.1968	462.9556	0.3871					
4062.8	33.14	0.6968641	5.97E-03	81.0783	219.9318	0.3687	0.5016611	9.73E-01	206.409	115.2756	1.7906					
4080.89	24.58	0.6205575	4.27E-02	130.9714	25.8236	5.0718	0.581032	2.73E-03	45.9354	16.998	2.7024					
4115.97	28.76	0.6641115	2.74E-03	2.644	26.2848	0.1006	0.5007267	9.60E-01	0.936	4.2156	0.2220					
4190.72	20.53	0.6738676	1.39E-02	168.6384	55.6296	3.0315	0.5280575	3.92E-01	54.756	117.858	0.4646					
4196.75	20.84	0.7327526	2.55E-03	558.48	203.2503	2.7477	0.588559	2.62E-02	204.2352	323.3856	0.6316					
4212.76	20.91	0.6163763	4.08E-02	25.53	32.6808	0.7812	0.5071896	8.20E-01	22.9785	26.4082	0.8701					
4275.94	24.94	0.6351916	1.62E-02	3.04	25.0104	0.1215	0.5359219	3.63E-02	1.6076	9.2601	0.1736					
4299.07	33.1	0.7073171	3.59E-04	761.1404	0	∞	0.5211275	2.51E-02	0	29.0235	0.0000					
4339.95	25.71	0.7498258	8.82E-05	5.1072	110.7648	0.0461	0.5727263	2.97E-02	20.7115	44.148	0.4691					
4345.89	33.59	0.7365854	6.70E-05	0.4308	45.9669	0.0094	0.5505606	9.96E-02	9.6509	12.1839	0.7921					
4352.86	20.17	0.7602787	9.31E-04	7874.892	2417.617	3.2573	0.5005191	9.93E-01	3036.4886	5914.8722	0.5134					
4396	25.81	0.6571429	1.09E-03	0	24.2327	0.0000	0.5413206	2.83E-02	2.8184	4.9385	0.5707					
4404.84	20.67	0.8017422	1.30E-04	1485.2024	345.0609	4.3042	0.5248391	6.07E-01	480.843	814.6206	0.5903					
4409.89	20	0.738676	1.96E-03	1669.232	483.0644	3.4555	0.5534676	2.00E-01	566.2848	1183.4006	0.4785					
4418.99	25.75	0.7254355	3.28E-04	14.1155	103.2393	0.1367	0.6814784	4.80E-14	6.5932	52.4	0.1258					
4507.08	26.25	0.7261324	1.37E-04	5.0836	57.1634	0.0889	0.5408534	1.64E-01	6.4335	13.4666	0.4777					
4522.11	26.26	0.6554007	3.59E-03	2.873	84.9422	0.0338	0.5069819	8.78E-01	25.248	27.3126	0.9244					
4538.09	26.27	0.6766551	5.69E-03	15.2252	152.881	0.0996	0.5447986	2.91E-01	68.5104	66.8304	1.0251					
4579.07	25.92	0.6571429	1.09E-03	0	20.4476	0.0000	0.5248131	2.96E-01	3.3488	4.666	0.7177					
4670.15	25.86	0.6571429	3.80E-02	245.7212	302.7582	0.8116	0.5509759	2.04E-01	107.9086	174.0648	0.6199					
4771.07	20.2	0.7930314	1.42E-04	1627.352	308.972	5.2670	0.5157288	7.15E-01	365.0432	954.772	0.3823					
4805.33	26.54	0.6132404	4.11E-02	18.751	99.6774	0.1881	0.5221138	3.40E-01	9.5752	40.8786	0.2342					
4847.12	26.74	0.6372822	4.18E-02	85.106	175.7103	0.4844	0.5164556	6.29E-01	41.3355	78.3993	0.5272					
4891.11	26.58	0.6731707	3.92E-03	26.105	215.6364	0.1211	0.5654589	7.87E-06	2.6768	63.2112	0.0423					
5026.57	26.53	0.6480836	3.11E-03	3.0614	140.8299	0.0217	0.5314576	2.24E-01	35.0392	54.5216	0.6427					
5043.13	26.6	0.7219512	2.22E-03	100.6965	317.745	0.3169	0.5177533	6.77E-01	115.7545	125.1477	0.9249					
5228.26	27	0.6445993	4.17E-02	184.7735	449.3988	0.4112	0.6072207	2.32E-03	88.011	301.651	0.2918					
5276.41	20.04	0.671777	2.26E-03	106.1493	3.6306	29.2374	0.5178312	4.45E-01	17.2602	44.16	0.3909					
5526.4	27.3	0.6592334	3.70E-03	10.7865	81.585	0.1322	0.5038414	9.32E-01	74.3648	70.9884	1.0476					
5574.25	23.2	0.7648084	7.83E-04	967.0936	365.6	2.6452	0.5116798	8.21E-01	569.0685	519.3405	1.0958					
5800.46	19.6	0.6794425	3.35E-03	1351.9176	35.019	38.6053	0.5141715	6.16E-01	49.8563	73.3376	0.6798					
6055.58	21.05	0.766899	3.37E-04	1985.702	148.8773	13.3378	0.519077	5.38E-01	33.27	118.3938	0.2810					

6211.74	20.28	0.6881533	1.14E-02	917.1351	276.044	3.3224	0.5430596	2.96E-01	183.768	488.2108	0.3764
6236.91	21.07	0.7491289	1.14E-03	1032.5968	241.3575	4.2783	0.583316	4.85E-02	543.9483	356.5552	1.5256
6237.14	31.39	0.6919861	4.36E-03	79.747	226.2786	0.3524	0.603561	1.01E-05	14.8939	103.048	0.1445
8837.41	21.06	0.9108014	3.75E-07	5375.3442	463.6527	11.5935	0.5292255	4.59E-01	583.306	841.1273	0.6935
8917.25	22.55	0.7811847	2.79E-04	691.504	164.1	4.2139	0.5129257	7.56E-01	95.73	156.0882	0.6133
10199.7	21.11	0.6926829	7.59E-03	699.7695	67.8776	10.3093	0.5410091	1.85E-01	56.904	156.1308	0.3645
10341.97	22.98	0.7052265	4.02E-03	1918.9746	477.8434	4.0159	0.5282392	2.90E-01	167.0603	414.3772	0.4032
10640.09	19.68	0.6696864	5.39E-03	378.5038	125.7984	3.0088	0.5044643	8.55E-01	20.888	86.5824	0.2412
10753.32	19.65	0.7087108	2.67E-03	3040.7552	501.976	6.0576	0.5160922	5.78E-01	357.1692	621.8567	0.5744
10946.01	20.81	0.6473868	5.63E-03	1766.256	20.8761	84.6066	0.5094477	2.26E-01	0	9.3513	0.0000
11721.44	19.58	0.6491289	7.83E-03	1162.1132	21.4152	54.2658	0.5142753	4.57E-01	45.7506	10.1101	4.5252
11967.55	20.47	0.7979094	6.86E-05	801.6681	63.945	12.5368	0.5236711	5.48E-01	133.335	143.4188	0.9297
14110.92	21.96	0.6783972	1.67E-02	1033.0015	196.9168	5.2459	0.6008098	9.48E-05	57.621	168.9888	0.3410
