# Supplementary material for: Urinary Proteomic Biomarkers for Diagnosis and Risk Stratification of Autosomal Dominant Polycystic Kidney Disease: A Multicentric Study
Source: PLoS One. 2013 Jan 10;8(1):e53016. doi: 10.1371/journal.pone.0053016 (PMC3542378; doi:10.1371/journal.pone.0053016)
Supplement: Table S2 — Characteristics of the 99 biomarkers correlated with height adjusted TKV. Shown are the peptide identification number in the dataset (Peptid ID), molecular mass (in Da) and normalized migration time (in min). Given are the Sperman's coefficient of rank correlation and the significance level (p-values). In addition, amino acid sequence (modified amino acids: p = hydroxyproline; k = hydroxylysine; m = oxidized methionine), parent protein name with the position of the first (start) and last (stop) amino acid, the SwissProt/TrEMBLEentry numbers and accession numbers are given. (PDF) [file pone.0053016.s002.pdf]

Supplementary Table 2:

Peptid ID	Mass (Da)	CE-Time (Min)	Spearman's rho	p-values	Sequence	Protein name	Start AA	Stop AA	UniProt entry name	Accession number
1577	840.4071	23.16555	-0.25814	1.758E-06	KGDTGPpGP	Collagen alpha-1(III) chain	629	637	CO3A1_HUMAN	gi124056490
2505	858.3934	23.2367	-0.27301	9.083E-06	SpGEAGRpG	Collagen alpha-1(I) chain	522	530	CO1A1_HUMAN	gi124056487
2510	858.4304	20.02662	0.28367	1.006E-05						
6543	924.438	33.6151	-0.26252	1.743E-05						
7408	935.4465	23.68105	-0.25310	2.826E-05	GRpGPpGPpG	Collagen alpha-1(I) chain	563	572	CO1A1_HUMAN	gi124056487
8800	950.4351	26.51207	-0.25396	4.324E-05						
10581	971.4839	19.37551	0.27123	4.442E-05						
11483	982.5169	24.37967	0.27376	5.519E-05						
14478	1040.475	25.05015	-0.31171	7.917E-05	SpGPDGKTGPp	Collagen alpha-1(I) chain	546	556	CO1A1_HUMAN	gi124056487
14512	1041.413	37.27865	-0.25225	9.055E-05						
14906	1050.477	26.92478	-0.27958	1.336E-04	MGPRGPpGPpG	Collagen alpha-1(I) chain	217	227	CO1A1_HUMAN	gi124056487
15216	1058.476	24.89302	0.32353	1.455E-04	TISRLEPED	Ig kappa chain V-III region NG9	79	87	KV303_HUMAN	gi125799
16910	1083.457	26.78053	-0.28913	1.642E-04						
17694	1096.483	26.07573	-0.32524	1.658E-04	ApGDRGEpGpP	Collagen alpha-1(I) chain	798	808	CO1A1_HUMAN	gi124056487
17829	1097.495	21.00157	0.30096	1.668E-04	AHVDDmPNAL	Hemoglobin subunit alpha	72	81	HBA_HUMAN	gi:57013850
18943	1114.492	25.55449	-0.28786	2.088E-04	SpGERGETGPp	Collagen alpha-1(III) chain	796	806	CO3A1_HUMAN	gi124056490
19214	1120.499	27.75569	0.29277	2.473E-04						
21294	1153.399	36.63236	-0.27058	2.482E-04						
22636	1169.661	19.97743	0.40071	2.553E-04						
23870	1189.597	21.17739	0.27147	2.598E-04	YGRAPQLRET	Alpha-1-microglobulin	151	160	AMBP_HUMAN	gi122801
25225	1213.653	19.94632	0.35454	2.599E-04						
25893	1223.571	19.39261	0.28652	2.658E-04	DHEGTHSTKRG	Fibrinogen alpha chain	612	622	FIBA_HUMAN	gi:1706799
26878	1238.531	26.59558	0.28932	2.948E-04						
27742	1251.62	22.52534	-0.26744	3.102E-04	DGVPGKDGPRGPT	Collagen alpha-1(III) chain	752	764	CO3A1_HUMAN	gi124056490
28132	1257.639	19.91857	0.34631	3.153E-04	TISEKTSDQIH	Antithrombin-III	142	152	ANT3_HUMAN	gi:113936
28561	1265.589	27.08673	-0.28069	3.329E-04	SpGPDGKTGPpGPA	Collagen alpha-1(I) chain	546	559	CO1A1_HUMAN	gi124056487
28747	1268.569	27.24816	0.32111	3.661E-04	SpGERGETGPpGP	Collagen alpha-1(III) chain	796	808	CO3A1_HUMAN	gi124056490
29411	1279.562	19.71609	-0.28062	4.315E-04						
30699	1299.583	22.38183	-0.25816	5.253E-04						
31517	1312.586	19.35954	-0.26985	5.654E-04						
32823	1332.537	21.73754	0.31266	5.836E-04						
33047	1336.605	27.13567	0.31124	5.954E-04						
34212	1358.587	26.07703	-0.26419	6.267E-04						
35199	1375.54	28.16391	-0.25030	6.814E-04						
35339	1378.613	28.822	-0.37254	7.312E-04	ApGEDGRpGPpGPQ	Collagen alpha-1(II) chain	580	593	CO2A1_HUMAN	gi124056489
36672	1403.568	21.75433	0.26278	7.374E-04						
37127	1410.655	22.32483	-0.33544	7.797E-04						
37340	1415.637	23.55322	0.30800	8.274E-04						
37461	1418.604	22.91072	-0.25518	8.631E-04						
38007	1426.609	19.82891	-0.28467	8.969E-04						
38266	1430.65	29.2403	0.26700	9.270E-04	DSEETRAAAPQAW	Drebrin	385	397	DREB_HUMAN	gi215274247
38991	1441.602	19.84575	-0.30908	9.371E-04						
39607	1447.696	19.47239	-0.25447	9.493E-04	DTDRFSSHVGGTLG	Inter-alpha-trypsin inhibitor heavy chain H4	863	876	ITIH4_HUMAN	gi:229463048
41485	1467.659	29.07289	0.27505	9.965E-04	SpGSpGPDGKTGPpGp	Collagen alpha-1(I) chain	543	558	CO1A1_HUMAN	gi124056487
42064	1480.656	29.87579	-0.27110	1.012E-03						
42662	1492.367	36.68159	-0.26154	1.023E-03						
44750	1525.669	30.3927	-0.31623	1.043E-03	YKTTPPVLDSDGSF	Ig gamma-1 chain C region	274	287	IGHG1_HUMAN	gi121039
48699	1591.74	30.38794	0.30651	1.066E-03	IGPpGPAGApGDKGESGP	Collagen alpha-1(I) chain	769	786	CO1A1_HUMAN	gi124056487
49122	1592.733	19.51922	-0.26765	1.069E-03						
49901	1607.608	19.881	-0.28555	1.118E-03						
51175	1630.739	20.64725	-0.26606	1.247E-03	EGSpGRDGSpGAKGDRG	Collagen alpha-1(I) chain	1021	1037	CO1A1_HUMAN	gi124056487
51948	1636.856	23.17649	0.31274	1.355E-03	LSALEEYTKKLNTQ	Apolipoprotein A-I	254	267	APOA1_HUMAN	gi113992
52730	1649.714	19.57861	-0.28225	1.416E-03						
53589	1663.627	19.8553	-0.28165	1.430E-03						
54424	1679.757	29.09789	0.29668	1.454E-03						
54525	1680.752	30.02747	-0.25659	1.458E-03	TGSpGSpGPDGKTGPpGPA	Collagen alpha-1(I) chain	541	559	CO1A1_HUMAN	gi124056487
55143	1692.798	30.88753	-0.27702	1.476E-03	PpGEAGKpGEQGVPGDLG	Collagen alpha-1(I) chain	651	668	CO1A1_HUMAN	gi124056487
55144	1692.799	27.77069	0.31171	1.487E-03						
57360	1734.664	19.89763	-0.25052	1.551E-03						
57378	1734.792	23.58	-0.28188	1.573E-03	GppGPPGKNGDDGEAGKPG	Collagen alpha-1(I) chain	221	239	CO1A1_HUMAN	gi124056487
59022	1766.998	24.1138	0.32091	1.589E-03	SVIDQSRVLNLGPITR	Uromodulin	591	606	UROM_HUMAN	gi137116
60242	1796.751	29.4527	-0.27182	1.598E-03	GEpGApGSKGDTGAKGEpGP	Collagen alpha-1(I) chain	434	453	CO1A1_HUMAN	gi124056487
61576	1825.796	31.93096	-0.25816	1.611E-03						

62080	1837.8	30.55694	-0.27400	1.633E-03	AVAHVDDMPNALSALSDL	Hemoglobin subunit alpha	70	87	HBA_HUMAN	gi:57013850
62547	1847.885	43.66552	-0.25342	1.651E-03	DAGPVGPpGPpGPpGPPGPPS	Collagen alpha-1(I) chain	1173	1193	CO1A1_HUMAN	gi124056487
63143	1859.828	24.41139	-0.26638	1.683E-03	NSGEpGApGSKGDTGAKGEp	Collagen alpha-1(I) chain	432	451	CO1A1_HUMAN	gi124056487
63209	1860.826	21.40014	-0.28392	1.842E-03	EGSpGRDGSpGAKGDRGET	Collagen alpha-1(I) chain	1021	1039	CO1A1_HUMAN	gi124056487
63812	1874.831	30.82379	-0.30766	1.858E-03						
65397	1902.817	24.58097	-0.34227	1.892E-03						
66185	1916.849	24.62585	-0.25769	1.940E-03	GNSGEPGApGSkGDTGAKGEp	Collagen alpha-1(I) chain	431	451	CO1A1_HUMAN	gi124056487
67012	1929.867	41.63768	0.32079	1.965E-03						
67263	1934.786	19.94225	-0.25588	1.990E-03						
67723	1945.881	41.90131	0.37427	2.120E-03						
68117	1954.966	25.35847	0.33285	2.245E-03	SHTSDSDVPSGVTEVVVKL	Clusterin	391	409	CLUS_HUMAN	gi116533
72343	2042.071	25.1431	0.29506	2.268E-03	EAIPMSIPPEVKFNKPFV	Alpha-1-antitrypsin	378	395	A1AT_HUMAN	gi1703025
73015	2059.005	33.08042	0.28118	2.307E-03	ELTETGVEAAAASAISVARTL	Plasma protease C1 inhibitor	448	468	IC1_HUMAN	gi124096
74187	2080.941	20.20103	-0.26576	2.359E-03	DAHKSEVAHRFKDLGEEN	Serum albumin	25	42	ALBU_HUMAN	gi113576
79135	2175.006	35.65454	0.27090	2.362E-03						
84216	2258.968	28.03755	-0.27031	2.493E-03						
86879	2312.013	33.48197	-0.26151	2.548E-03						
90840	2389.241	22.39921	0.26016	2.631E-03	MIEQNTKSPLFMGKVVNPTQK	Alpha-1-antitrypsin	398	418	A1AT_HUMAN	gi1703025
90924	2391.199	22.62451	0.26209	2.699E-03	AAHLPAEFTPAVHASLDKFLASV	Hemoglobin subunit alpha	111	133	HBA_HUMAN	gi:57013850
91421	2405.222	22.47371	0.27330	2.699E-03	MIEQNTKSPLFmGKVVNPTQK	Alpha-1-antitrypsin	398	418	A1AT_HUMAN	gi1703025
114825	2926.305	29.21586	-0.25603	2.701E-03						
115050	2932.321	34.14716	0.27337	2.749E-03						
118694	3023.356	24.55931	-0.28337	2.870E-03						
121775	3092.464	31.24934	-0.36311	2.934E-03	ADGQPGAkGEPGDAGAKGDAGPPGPAGpAGpPGPIG	Collagen alpha-1(I) chain	819	854	CO1A1_HUMAN	gi124056487
122400	3108.454	31.28399	-0.31208	2.951E-03	ADGQpGAKGEpGDAGAKGDAGpPGPAGPAGPPGpIG	Collagen alpha-1(I) chain	819	854	CO1A1_HUMAN	gi124056487
123671	3149.46	31.24549	-0.25880	3.002E-03	GADGQPGAKGEpGDAGAKGDAGPpGPAGpAGPPGPIG	Collagen alpha-1(I) chain	818	854	CO1A1_HUMAN	gi124056487
125811	3223.481	30.30034	-0.34680	3.033E-03						
136432	3547.638	29.88238	-0.25960	3.065E-03						
139975	3651.658	31.87444	-0.25492	3.119E-03						
145768	3885.854	33.59788	-0.25544	3.181E-03						
148717	4008.81	23.42187	0.29435	3.248E-03						
152341	4143.978	26.74992	-0.25267	3.289E-03						
156175	4292.969	26.24762	-0.30449	3.344E-03						
163274	4563.973	33.72311	-0.29391	3.399E-03						
179692	6930.93	19.71085	-0.29090	3.633E-03						
191081	15815.39	19.42882	0.27283	3.663E-03						
