# Supplementary material for: Correction: Urinary Proteomic Biomarkers for Diagnosis and Risk Stratification of Autosomal Dominant Polycystic Kidney Disease: A Multicentric Study
Source: PLoS One. 2013 Aug 6;8(8):10.1371/annotation/9281c713-d253-4a1a-8255-92e691e77a24. doi: 10.1371/annotation/9281c713-d253-4a1a-8255-92e691e77a24 (PMC3735642; doi:10.1371/annotation/9281c713-d253-4a1a-8255-92e691e77a24)
Supplement: Supplementary file 1 [file pone.9281c713-d253-4a1a-8255-92e691e77a24.s001.pdf]

| Peptide ID | Mass (Da) | CE-Time (Min) | Spearman's rho | p-value | Sequence | Protein name | Start AA | Stop AA | UniProt entry name | Accession number |
|---|---|---|---|---|---|---|---|---|---|---|
| 1577 | 840.4071 | 23.16555 | -0.25814 | 2.701E-03 | KGDTGPpGP | Collagen alpha-1(III) chain | 629 | 637 | CO3A1_HUMAN | gi124056490 |
| 2505 | 858.3934 | 23.2367 | -0.27301 | 1.476E-03 | SpGEAGRpG | Collagen alpha-1(I) chain | 522 | 530 | CO1A1_HUMAN | gi124056487 |
| 2510 | 858.4304 | 20.02662 | 0.28367 | 9.371E-04 | | | | | | |
| 6543 | 924.438 | 33.6151 | -0.26252 | 2.268E-03 | | | | | | |
| 7408 | 935.4465 | 23.68105 | -0.25310 | 3.289E-03 | GRpGPpGPpG | Collagen alpha-1(I) chain | 563 | 572 | CO1A1_HUMAN | gi124056487 |
| 8800 | 950.4351 | 26.51207 | -0.25396 | 3.181E-03 | | | | | | |
| 10581 | 971.4839 | 19.37551 | 0.27123 | 1.589E-03 | | | | | | |
| 11483 | 982.5169 | 24.37967 | 0.27376 | 1.430E-03 | | | | | | |
| 14478 | 1040.475 | 25.05015 | -0.31171 | 2.598E-04 | SpGPDGKTGPp | Collagen alpha-1(I) chain | 546 | 556 | CO1A1_HUMAN | gi124056487 |
| 14512 | 1041.413 | 37.27865 | -0.25225 | 3.399E-03 | | | | | | |
| 14906 | 1050.477 | 26.92478 | -0.27958 | 1.118E-03 | MGPRGPpGPpG | Collagen alpha-1(I) chain | 217 | 227 | CO1A1_HUMAN | gi124056487 |
| 15216 | 1058.476 | 24.89302 | 0.32353 | 1.455E-04 | TISRLEPED | Ig kappa chain V-III region NG9 | 79 | 87 | KV303_HUMAN | gi125799 |
| 16910 | 1083.457 | 26.78053 | -0.28913 | 7.374E-04 | | | | | | |
| 17694 | 1096.483 | 26.07573 | -0.32524 | 1.336E-04 | ApGDRGEpGpP | Collagen alpha-1(I) chain | 798 | 808 | CO1A1_HUMAN | gi124056487 |
| 17829 | 1097.495 | 21.00157 | 0.30096 | 4.315E-04 | AHVDDmPNAL | Hemoglobin subunit alpha | 72 | 81 | HBA_HUMAN | gi:57013850 |
| 18943 | 1114.492 | 25.55449 | -0.28786 | 7.797E-04 | SpGERGETGPp | Collagen alpha-1(III) chain | 796 | 806 | CO3A1_HUMAN | gi124056490 |
| 19214 | 1120.499 | 27.75569 | 0.29277 | 6.267E-04 | | | | | | |
| 21294 | 1153.399 | 36.63236 | -0.27058 | 1.633E-03 | | | | | | |
| 22636 | 1169.661 | 19.97743 | 0.40071 | 1.758E-06 | | | | | | |
| 23870 | 1189.597 | 21.17739 | 0.27147 | 1.573E-03 | YGRAPQLRET | Alpha-1-microglobulin | 151 | 160 | AMBP_HUMAN | gi122801 |
| 25225 | 1213.653 | 19.94632 | 0.35454 | 2.826E-05 | | | | | | |
| 25893 | 1223.571 | 19.39261 | 0.28652 | 8.274E-04 | DHEGTHSTKRG | Fibrinogen alpha chain | 612 | 622 | FIBA_HUMAN | gi:1706799 |
| 26878 | 1238.531 | 26.59558 | 0.28932 | 7.312E-04 | | | | | | |
| 27742 | 1251.62 | 22.52534 | -0.26744 | 1.858E-03 | DGVPGKDGPRGPT | Collagen alpha-1(III) chain | 752 | 764 | CO3A1_HUMAN | gi124056490 |
| 28132 | 1257.639 | 19.91857 | 0.34631 | 4.442E-05 | TISEKTSDQIH | Antithrombin-III | 142 | 152 | ANT3_HUMAN | gi:113936 |
| 28561 | 1265.589 | 27.08673 | -0.28069 | 1.066E-03 | SpGPDGKTGPpGPA | Collagen alpha-1(I) chain | 546 | 559 | CO1A1_HUMAN | gi124056487 |
| 28747 | 1268.569 | 27.24816 | 0.32111 | 1.642E-04 | SpGERGETGPpGP | Collagen alpha-1(III) chain | 796 | 808 | CO3A1_HUMAN | gi124056490 |
| 29411 | 1279.562 | 19.71609 | -0.28062 | 1.069E-03 | | | | | | |
| 30699 | 1299.583 | 22.38183 | -0.25816 | 2.699E-03 | | | | | | |
| 31517 | 1312.586 | 19.35954 | -0.26985 | 1.683E-03 | | | | | | |
| 32823 | 1332.537 | 21.73754 | 0.31266 | 2.482E-04 | | | | | | |
| 33047 | 1336.605 | 27.13567 | 0.31124 | 2.658E-04 | | | | | | |
| 34212 | 1358.587 | 26.07703 | -0.26419 | 2.120E-03 | | | | | | |
| 35199 | 1375.54 | 28.16391 | -0.25030 | 3.663E-03 | | | | | | |
| 35339 | 1378.613 | 28.822 | -0.37254 | 1.006E-05 | ApGEDGRpGPpGPQ | Collagen alpha-1(II) chain | 580 | 593 | CO2A1_HUMAN | gi124056489 |
| 36672 | 1403.568 | 21.75433 | 0.26278 | 2.245E-03 | | | | | | |
| 37127 | 1410.655 | 22.32483 | -0.33544 | 7.917E-05 | | | | | | |
| 37340 | 1415.637 | 23.55322 | 0.30800 | 3.102E-04 | | | | | | |
| 37461 | 1418.604 | 22.91072 | -0.25518 | 3.033E-03 | | | | | | |
| 38007 | 1426.609 | 19.82891 | -0.28467 | 8.969E-04 | | | | | | |
| 38266 | 1430.65 | 29.2403 | 0.26700 | 1.892E-03 | DSEETRAAAPQAW | Drebrin | 385 | 397 | DREB_HUMAN | gi215274247 |
| 38991 | 1441.602 | 19.84575 | -0.30908 | 2.948E-04 | | | | | | |
| 39607 | 1447.696 | 19.47239 | -0.25447 | 3.119E-03 | DTDRFSSHVGGTLG | Inter-alpha-trypsin inhibitor heavy chain H4 | 863 | 876 | ITIH4_HUMAN | gi:229463048 |
| 41485 | 1467.659 | 29.07289 | 0.27505 | 1.355E-03 | SpGSpGPDGKTGPpGp | Collagen alpha-1(I) chain | 543 | 558 | CO1A1_HUMAN | gi124056487 |
| 42064 | 1480.656 | 29.87579 | -0.27110 | 1.598E-03 | | | | | | |
| 42662 | 1492.367 | 36.68159 | -0.26154 | 2.359E-03 | | | | | | |
| 44750 | 1525.669 | 30.3927 | -0.31623 | 2.088E-04 | YKTTPPVLDSDGSF | Ig gamma-1 chain C region | 274 | 287 | IGHG1_HUMAN | gi121039 |
| 48699 | 1591.74 | 30.38794 | 0.30651 | 3.329E-04 | IGPpGPAGApGDKGESGP | Collagen alpha-1(I) chain | 769 | 786 | CO1A1_HUMAN | gi124056487 |
| 49122 | 1592.733 | 19.51922 | -0.26765 | 1.842E-03 | | | | | | |
| 49901 | 1607.608 | 19.881 | -0.28555 | 8.631E-04 | | | | | | |
| 51175 | 1630.739 | 20.64725 | -0.26606 | 1.965E-03 | EGSpGRDGSpGAKGDRG | Collagen alpha-1(I) chain | 1021 | 1037 | CO1A1_HUMAN | gi124056487 |

| | | | | | | | | | | |
|---|---|---|---|---|---|---|---|---|---|---|
| 51948 | 1636.856 | 23.17649 | 0.31274 | 2.473E-04 | LSALEEYTKKLNTQ | Apolipoprotein A-I | 254 | 267 | APOA1_HUMAN | gi113992 |
| 52730 | 1649.714 | 19.57861 | -0.28225 | 9.965E-04 | | | | | | |
| 53589 | 1663.627 | 19.8553 | -0.28165 | 1.023E-03 | | | | | | |
| 54424 | 1679.757 | 29.09789 | 0.29668 | 5.253E-04 | | | | | | |
| 54525 | 1680.752 | 30.02747 | -0.25659 | 2.870E-03 | TGSpGSpGPDGKTGPpGPA | Collagen alpha-1(I) chain | 541 | 559 | CO1A1_HUMAN | gi124056487 |
| 55143 | 1692.798 | 30.88753 | -0.27702 | 1.247E-03 | PpGEAGKpGEQGVPGDLG | Collagen alpha-1(I) chain | 651 | 668 | CO1A1_HUMAN | gi124056487 |
| 55144 | 1692.799 | 27.77069 | 0.31171 | 2.599E-04 | | | | | | |
| 57360 | 1734.664 | 19.89763 | -0.25052 | 3.633E-03 | | | | | | |
| 57378 | 1734.792 | 23.58 | -0.28188 | 1.012E-03 | GppGPPGKNGDDGEAGKPG | Collagen alpha-1(I) chain | 221 | 239 | CO1A1_HUMAN | gi124056487 |
| 59022 | 1766.998 | 24.1138 | 0.32091 | 1.658E-04 | SVIDQSRVLNLGPITR | Uromodulin | 591 | 606 | UROM_HUMAN | gi137116 |
| 60242 | 1796.751 | 29.4527 | -0.27182 | 1.551E-03 | GEpGApGSKGDTGAKGEpGP | Collagen alpha-1(I) chain | 434 | 453 | CO1A1_HUMAN | gi124056487 |
| 61576 | 1825.796 | 31.93096 | -0.25816 | 2.699E-03 | | | | | | |
| 62080 | 1837.8 | 30.55694 | -0.27400 | 1.416E-03 | AVAHVDDMPNALSALSDL | Hemoglobin subunit alpha | 70 | 87 | HBA_HUMAN | gi:57013850 |
| 62547 | 1847.885 | 43.66552 | -0.25342 | 3.248E-03 | DAGPVGPpGPpGPpGPPGPPS | Collagen alpha-1(I) chain | 1173 | 1193 | CO1A1_HUMAN | gi124056487 |
| 63143 | 1859.828 | 24.41139 | -0.26638 | 1.940E-03 | NSGEpGApGSKGDTGAKGEp | Collagen alpha-1(I) chain | 432 | 451 | CO1A1_HUMAN | gi124056487 |
| 63209 | 1860.826 | 21.40014 | -0.28392 | 9.270E-04 | EGSpGRDGSpGAKGDRGET | Collagen alpha-1(I) chain | 1021 | 1039 | CO1A1_HUMAN | gi124056487 |
| 63812 | 1874.831 | 30.82379 | -0.30766 | 3.153E-04 | | | | | | |
| 65397 | 1902.817 | 24.58097 | -0.34227 | 5.519E-05 | | | | | | |
| 66185 | 1916.849 | 24.62585 | -0.25769 | 2.749E-03 | GNSGEPGApGSkGDTGAKGEp | Collagen alpha-1(I) chain | 431 | 451 | CO1A1_HUMAN | gi124056487 |
| 67012 | 1929.867 | 41.63768 | 0.32079 | 1.668E-04 | | | | | | |
| 67263 | 1934.786 | 19.94225 | -0.25588 | 2.951E-03 | | | | | | |
| 67723 | 1945.881 | 41.90131 | 0.37427 | 9.083E-06 | | | | | | |
| 68117 | 1954.966 | 25.35847 | 0.33285 | 9.055E-05 | SHTSDSDVPSGVTEVVVKL | Clusterin | 391 | 409 | CLUS_HUMAN | gi116533 |
| 72343 | 2042.071 | 25.1431 | 0.29506 | 5.654E-04 | EAIPMSIPPEVKFNKPFV | Alpha-1-antitrypsin | 378 | 395 | A1AT_HUMAN | gi1703025 |
| 73015 | 2059.005 | 33.08042 | 0.28118 | 1.043E-03 | ELTETGVEAAAASAISVARTL | Plasma protease C1 inhibitor | 448 | 468 | IC1_HUMAN | gi124096 |
| 74187 | 2080.941 | 20.20103 | -0.26576 | 1.990E-03 | DAHKSEVAHRFKDLGEEN | Serum albumin | 25 | 42 | ALBU_HUMAN | gi113576 |
| 79135 | 2175.006 | 35.65454 | 0.27090 | 1.611E-03 | | | | | | |
| 84216 | 2258.968 | 28.03755 | -0.27031 | 1.651E-03 | | | | | | |
| 86879 | 2312.013 | 33.48197 | -0.26151 | 2.362E-03 | | | | | | |
| 90840 | 2389.241 | 22.39921 | 0.26016 | 2.493E-03 | MIEQNTKSPLFMGKVVNPTQK | Alpha-1-antitrypsin | 398 | 418 | A1AT_HUMAN | gi1703025 |
| 90924 | 2391.199 | 22.62451 | 0.26209 | 2.307E-03 | AAHLPAEFTPAVHASLDKFLASV | Hemoglobin subunit alpha | 111 | 133 | HBA_HUMAN | gi:57013850 |
| 91421 | 2405.222 | 22.47371 | 0.27330 | 1.458E-03 | MIEQNTKSPLFmGKVVNPTQK | Alpha-1-antitrypsin | 398 | 418 | A1AT_HUMAN | gi1703025 |
| 114825 | 2926.305 | 29.21586 | -0.25603 | 2.934E-03 | | | | | | |
| 115050 | 2932.321 | 34.14716 | 0.27337 | 1.454E-03 | | | | | | |
| 118694 | 3023.356 | 24.55931 | -0.28337 | 9.493E-04 | | | | | | |
| 121775 | 3092.464 | 31.24934 | -0.36311 | 1.743E-05 | ADGQPGAkGEPGDAGAKGDAGPPGPAGpAGpPGPIG | Collagen alpha-1(I) chain | 819 | 854 | CO1A1_HUMAN | gi124056487 |
| 122400 | 3108.454 | 31.28399 | -0.31208 | 2.553E-04 | ADGQpGAKGEpGDAGAKGDAGpPGPAGPAGPPGpIG | Collagen alpha-1(I) chain | 819 | 854 | CO1A1_HUMAN | gi124056487 |
| 123671 | 3149.46 | 31.24549 | -0.25880 | 2.631E-03 | GADGQPGAKGEpGDAGAKGDAGPpGPAGpAGPPGPIG | Collagen alpha-1(I) chain | 818 | 854 | CO1A1_HUMAN | gi124056487 |
| 125811 | 3223.481 | 30.30034 | -0.34680 | 4.324E-05 | | | | | | |
| 136432 | 3547.638 | 29.88238 | -0.25960 | 2.548E-03 | | | | | | |
| 139975 | 3651.658 | 31.87444 | -0.25492 | 3.065E-03 | | | | | | |
| 145768 | 3885.854 | 33.59788 | -0.25544 | 3.002E-03 | | | | | | |
| 148717 | 4008.81 | 23.42187 | 0.29435 | 5.836E-04 | | | | | | |
| 152341 | 4143.978 | 26.74992 | -0.25267 | 3.344E-03 | | | | | | |
| 156175 | 4292.969 | 26.24762 | -0.30449 | 3.661E-04 | | | | | | |
| 163274 | 4563.973 | 33.72311 | -0.29391 | 5.954E-04 | | | | | | |
| 179692 | 6930.93 | 19.71085 | -0.29090 | 6.814E-04 | | | | | | |
| 191081 | 15815.39 | 19.42882 | 0.27283 | 1.487E-03 | | | | | | |
